# Investigation of Genetic Determinants of Glioma Immune Phenotype by Integrative Immunogenomic Scale Analysis

**DOI:** 10.3389/fimmu.2021.557994

**Published:** 2021-06-16

**Authors:** Binghao Zhao, Yuekun Wang, Yaning Wang, Congxin Dai, Yu Wang, Wenbin Ma

**Affiliations:** Departments of Neurosurgery, Peking Union Medical College Hospital, Chinese Academy of Medical Sciences and Peking Union Medical College, Beijing, China

**Keywords:** immunogenomic analysis, microenvironment, immune phenotype, glioma, biometrics

## Abstract

The immunosuppressive mechanisms of the surrounding microenvironment and distinct immunogenomic features in glioblastoma (GBM) have not been elucidated to date. To fill this gap, useful data were extracted from The Cancer Genome Atlas (TCGA), the Chinese Glioma Genome Atlas (CGGA), GSE16011, GSE43378, GSE23806, and GSE12907. With the ssGSEA method and the ESTIMATE and CIBERSORT algorithms, four microenvironmental signatures were used to identify glioma microenvironment genes, and the samples were reasonably classified into three immune phenotypes. The molecular and clinical features of these phenotypes were characterized *via* key gene set expression, tumor mutation burden, fraction of immune cell infiltration, and functional enrichment. Exhausted CD8+ T cell (GET) signature construction with the predictive response to commonly used antitumor drugs and peritumoral edema assisted in further characterizing the immune phenotype features. A total of 2,466 glioma samples with gene expression profiles were enrolled. Tumor purity, ESTIMATE, and immune and stromal scores served as the 4 microenvironment signatures used to classify gliomas into immune-high, immune-middle and immune-low groups, which had distinct immune heterogeneity and clinicopathological characteristics. The immune-H phenotype had higher expression of four immune signatures; however, most checkpoint molecules exhibited poor survival. Enriched pathways among the subtypes were related to immunity. The GET score was similar among the three phenotypes, while immune-L was more sensitive to bortezomib, cisplatin, docetaxel, lapatinib, and rapamycin prescriptions and displayed mild peritumor edema. The three novel immune phenotypes with distinct immunogenetic features could have utility for understanding glioma microenvironment regulation and determining prognosis. These results contribute to classifying glioma subtypes, remodeling the immunosuppressive microenvironment and informing novel cancer immunotherapy in the era of precision immuno-oncology.

## Introduction

Gliomas are the most common and malignant primary tumors in the central nervous system (CNS) and have a highly invasive nature (1, 2). The discovery of the lymphatic system in the CNS has aroused inspiration to provide a novel theoretical foundation and new prospects for immunotherapy in brain tumors, and previous literature has demonstrated the mutual interactions between gliomas and the immune system (3, 4). Multiple related biological processes influencing CNS immune surveillance, such as the PI3K/Akt pathway, FAK, the IGF pathway, the STAT3 pathway, chemokines, HIF-1α, IL-6, TGF-β, PD-1/PD-L1, and CTLA-4, could individually impact immunosurveillance (5–8). Since entering the era of precision oncology, the molecular profiles of gliomas have been well studied. Mutations in the isocitrate dehydrogenase 1 (IDH1) gene, 1p/19q codeletion, methylguanine methyltransferase (MGMT) promoter methylation, tumor protein 53 (TP53), and telomerase reverse transcriptase (TERT) promoters are becoming treatment targets or prognostic biomarkers (9–11). Monoclonal antibodies (mAbs) against PD-1/PD-L1 show satisfying overall survival (OS) in melanoma and non-small cell lung cancer (NSCLC), but there is limited survival benefit in glioma (12). The unique immune-privileged microenvironment due to the inherent expression of immunosuppressive cytokines, such as PD-1, TGF-β, and IL-10, and the lack of antigen-presenting cells (APCs) in the CNS present obstacles for the efficacy of immunotherapy in glioblastoma (GBM) (13). The development of more novel and effective therapies will require a deep understanding of the tumor immunosuppressive microenvironment.

Direct interactions between tumor and immune cells can result in suppression of natural killer (NK) cell activity mediated by HLA molecules (including HLA-E and HLA-G) (14), immune cell apoptosis *via* tumor necrosis factor receptor superfamily member 6 (TNFRSF6, known as FAS) and the FAS-ligand interaction (15), or triggering of inhibitory T cell checkpoints by PD-L1 (16). The hypofunctional state of T cells known as T-cell exhaustion was identified by the accumulation of coinhibitory checkpoints (17). Of note, the paucity of T cells in the glioma microenvironment is striking in contrast to the levels in other “hot tumors”, and some studies have suggested that glioma-associated myeloid cells are immunosuppressive with an M2-like phenotype and require peripheral dendritic cells (DCs) to elicit an immune response (18). Indeed, the exact mechanism of immune suppression is still obscure. In this study, we employed 2,466 samples to properly classify glioma into immune phenotypes according to distinct immunogenomic features based on microenvironment-related genes. Then, we validated and identified microenvironmental processes, explored immune alterations, and characterized immunosuppressive mechanisms. The immune landscape may inspire glioma subtype classification, remodeling of the immunosuppressive microenvironment and development of new therapies.

## Methods

### Data Acquisition and Filtration

Data from glioma patients from six mRNA databases were extracted from TCGA database (RNA-sequencing (RNA-seq) for GBM, n = 169, microarray, n = 539) (http://cancergenome.nih.gov/), the CGGA database (RNA-seq, n = 1018, microarray, n = 301) (http://www.cgga.org.cn), the GSE16011 database (microarray, n = 276), the GSE43378 database (microarray, n = 50), the GSE23806 database (microarray, n = 92) and the GSE12907 database (microarray, n = 21). Complete clinical information was obtained from TCGA (http://cancergenome.nih.gov/, n = 708) and GCCA (http://www.cgga.org.cn, n = 1319). Somatic mutations and single nucleotide polymorphisms (SNPs) of gliomas were obtained from the TCGA database (http://cancergenome.nih.gov/, n=901, gene number n = 13,389). RNA-seq data downloaded in FPKM values from TCGA were normalized and transformed into transcripts per kilobase million values. RNA expression of gliomas was assessed with the Affymetrix microarray platform in the Gene Expression Omnibus (GEO) database (GSE16011, GSE43378, GSE23806, and GSE12907). Data were filtered to exclude samples without mRNA expression or clear histology, and the genomic data were normalized and analyzed within lanes, between lanes, and per quantile using the “limma” and “DESeq2” R packages. In this study, TCGA and CGGA were mainly treated as the training sets, and GEO databases were regarded as the validation sets.

### Immune Phenotype Classification

In the glioma microenvironment, immune and stromal cells are two key types of nontumor components and have been indicated to be significant for the diagnosis and prognosis of tumors. Yoshihara et al (19) designed the ESTIMATE algorithm to compute immune and stromal cell scores to predict the infiltration of these nontumor cells. The authors used ESTIMATE to evaluate immune scores, ESTIMATE scores, stromal scores and tumor purity scores in each tumor sample with the aim of determining the immune infiltration level.

Single-sample gene set enrichment analysis (ssGSEA), which assisted in quantifying the enrichment level of an immune cell/signature, pathway or biological process in a tumor sample, was used to assess the gene score of every gene set for every sample (20). The enrichment-related score represented the level at which the genes in the gene set were synchronously up- or downregulated in the sample. The infiltration of immune cells in the microenvironment was determined by 29 immune cell types: NK cells, effector memory CD4+ T cells, activated B cells, monocytes, memory B cells, activated CD4+ T cells, type 2 T helper cells, dendritic cells, neutrophils, macrophages, effector memory CD8+ T cells, myeloid-derived suppressor cells (MDSCs), immature B cells, mast cells, and regulatory T cells, and glioma samples were hierarchically clustered into “immune-high (immune-H)”, “immune-middle (immune-M)” and “immune-low (immune-L)” groups. Separation of gene expression patterns between immune phenotypes was evaluated by the principal component analysis (PCA) algorithm with the PCA1, PCA2, and PCA3 top three dimensions (21). Visualization was performed with the “GSVA”, “GSEABase”, “ComplexHeatmap”, “estimate”, and “ggplot” public packages.

### Quantification of Molecular and Genomic Features

Tumor mutation burden (TMB) was defined as the total count of somatic mutations per megabase in each tumor sample. We used the MATH algorithm (22), which assessed the width of the allele frequency distribution, to evaluate the intratumor heterogeneity level of tumor samples. Further intratumor heterogeneity scores were quantified using the function “math. Score” in the “maftools” package with downloaded “maf” files based on the hg19 sequencing platform. Comparisons of the somatic mutations and SNP sites among immune phenotypes in distinct populations (low-grade glioma (LGG) and GBM samples) were displayed to investigate the discrepancies by the “maftools” and “GenVisR” packages.

### Survival Analysis

Available clinicopathological factors (e.g., sex, age, treatment options, histological subtype, and classic mutations) were collected from the TCGA and CGGA datasets to estimate the association between these factors as well as the immune phenotypes and prognosis with univariable and multivariable Cox analysis (uniCox, multiCox) and proportional hazard models. We compared survival differences among immune-specific phenotypes of glioma in distinct groups using Kaplan-Meier curves and the log-rank test with normalized clinical data.

### Estimation of the Proportions of Immune Cell Types

CIBERSORT is an algorithm designed to characterize the cell composition of complex tissues based on their gene expression profiles, and it is highly consistent with real-life estimations in many cancers. A leukocyte gene signature matrix employing 547 genes, which was defined as LM22, was used to quantify 22 immune cell types (23). These 22 types of immune cells mainly include myeloid subtypes, NK cells, plasma cells, naive and memory B cells and T cells. We used the CIBERSORT method to investigate the fraction of the 22 immune cell types in each derived phenotype and identify the characteristics of infiltrating cells in the glioma microenvironment.

### Identification of a Gene Signature for Exhausted CD8+ T Cells

CD8+ T lymphocytes are regarded as a critical component of antitumor immunity, while immune invasion often occurs during the development of T cell exhaustion, characterized by the progressive accumulation of coinhibitory checkpoints, including PD-1, PD-L1, CTLA-4, TIM-3, and LAG-3 (17). We defined a gene expression signature of exhausted CD8+ T cells with integrative bioinformatics through publicly available NSCLC data considering the data quality and availability. We obtained an RNA-seq dataset of intratumoral CD8+ T cells showing high or no PD-1 (PDCD1) expression in a published study (24), and we generated an upregulated PD-1-positive gene list from another previous study (25). Pearson correlation analysis was conducted using the upregulated PD-1-positive gene list in the TCGA (microarray+ RNA-seq cohort) and CGGA (microarray+ RNA-seq cohort) datasets with an adjusted P-value < 0.05 and |correlation efficiency| > 0.25 as the eligibility criteria. In total, a 5-gene signature was identified in the glioma database, and an exhausted CD8+ T cell (GET) score was quantified in a tumor by conducting ssGSEA to obtain the ssGSEA score. In combination with clinical and molecular profiles, the prognostic and predictive values of the GET score were determined through different immune phenotypes.

### Correlation and Functional Analysis

Gene Ontology (GO) and Kyoto Encyclopedia of Genes and Genomes (KEGG) pathway analyses were performed on genes differentially expressed between the immune-high and immune-low groups. Gene set enrichment analysis (GSEA) was carried out to identify the group of genes enriched either in the immune-high or immune-low group with cutoffs of a P-value < 0.1 and a false discovery rate (FDR) < 0.05 (26). Gene set variation analysis (GSVA) is a nonparametric and unsupervised method estimating the variations of samples in analyzed datasets in pathways and biological process (27). The gene sets of “c2.cp.kegg.v6.2.-symbols” used were captured from the MSigDB website for GSVA, with an adjusted P-value < 0.05 considered statistically significant. Correlation plots were constructed that primarily focused on the interactions between IDH1 and other key immune-related genes identified from the GSEA with a P filter = 0.001. A Sankey diagram was constructed to show the correlations between checkpoints and the GSEA-identified genes. Visualization of the unction analyses was realized *via* the “circlize” (28), “circus” (29), “clusterProfiler”, and “ggalluvial” (30) packages.

### Prediction of the Chemo/TargetedTherapy Response

Intended chemotherapeutic and targeted responses of glioma samples were evaluated by the largest publicly available pharmacogenomics database (Pharmaceutical Sensitivity Genomics in Cancer (GDSC) https://www.cancerrxgene.org/) (31). GDSC contains drug sensitivity information from nearly 75000 experiments and responses to 138 anticancer drugs across almost 700 cell lines. The database provides a unique source relevant to mainstream drug sensitivity and genomic datasets to inspire new discoveries on cancer therapeutic biomarkers. GDSC is also utilized due to its visualization capability. The evaluation procedure was conducted *via* the R software package “pRRophetic”, half-maximal inhibitor concentration (IC50), and the evaluation accuracy was determined by ridge regression and 10-fold cross-validation using the GDSC dataset (32, 33). Different chemotherapeutic and targeted responses among the three phenotypes were analyzed by one-way analysis of variance (ANOVA) or the Kruskal-Wallis test (K-W test) based on the results of the normal distribution criteria test. The response to commonly used chemotherapy or targeted therapies was compared according to immune phenotype, although some drugs were not formally approved for utility in brain tumors.

### Peri-Tumoral Edema Characteristics

To detect the variations in some radiomics features of classified immune phenotypes, MR images (MRIs) of patients from the TCGA dataset were obtained from the Cancer Imaging Archive. TCGA-GBM and TCGA-LGG cohorts in the Cancer Imaging Archive (http://www.cancerimagingarchive.net) were specifically selected and matched with existing results. Eligible tumor contrast enhancement images were determined after a discussion with three skilled neurosurgeons (Zhao B, Xing H, Wang Y) on the author list. Radiomics features of tumors included tumor size, enhancement, noncontrast-enhancing tumor (nCET), necrosis, edema, cysts, multifocality, contact with ventricles or neocortex and location based on a previous study (34). Features such as multifocality, enhancement, location and edema were revealed to have molecular signature correlations with glioma, such as IDH mutation or MGMT promoter methylation; edema and necrosis were regarded as poor survival imaging markers (34, 35). Edema associated with both molecular phenotypes and prognosis was the focus of investigation to facilitate identification of noninvasive acquired markers and features of the classified glioma phenotypes. A mild (or no) region of edema (-) was regarded as edema extending ≤ 1 cm from the margin of the tumor; otherwise, it was treated as moderate to severe (+) (36). The evaluations were all based on eligible T2-weighted images.

### Statistical and Bioinformatics Analyses

Statistical analyses were conducted using R software (version 3.5.3), and other statistical methods are mentioned throughout the article. Bioinformatics analysis was conducted mainly following the methods of Thorsson et al (37). A two-sided P < 0.05 was considered to be significant unless otherwise specified. The public packages used are mentioned throughout this paper.

## Results

### ssGSEA and Independent Immune Phenotype Classification

**Figure 1:**
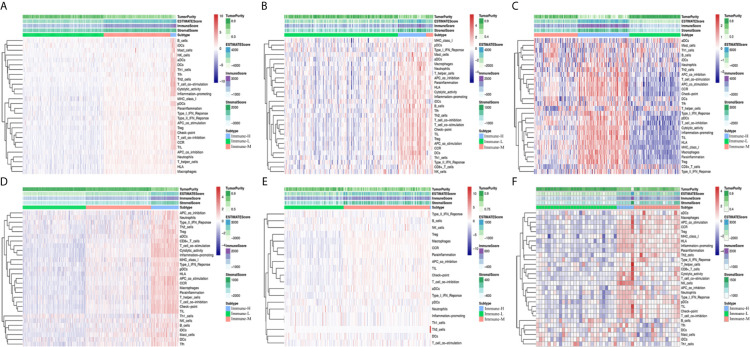
Immune phenotype classification and four glioma immune microenvironment signatures identification. **(A–F)** Heatmaps showing three immune phenotypes, tumor purity, ESTIMATE, immune and stromal scores in the glioma microenvironment of samples from the TCGA microarray, TCGA GBM RNA-seq, CGGA microarray, CGGA RNA-seq, GSE16011, and GSE43378 cohorts.

After excluding the normal tissues (5 normal samples in the TCGA RNA-seq database), tumor samples with distinct extension of inflammatory cell infiltration were classified into “immune-L”, “immune-M” and “immune-H” phenotypes with ssGSEA incorporating 29 types of immune cell lineages, such as helper T cells, cytotoxic T cells, myeloid cells, monocytes, NK cells, dendritic cells, and T cells. The numbers of samples falling into the immune-L, immune-M, and immune-H phenotypes were 283, 234 and 21 in the TCGA microarray data; 129, 8 and 32 in the TCGA-GBM RNA-seq data; 105, 90 and 106 in the CGGA microarray cohort; 413, 425 and 180 in the CGGA RNA-seq cohort; 112, 162 and 2 in GSE16011; 28, 16 and 6 in GSE43378; 87, 2 and 3 in GSE23806; and 9, 10 and 2 in GSE12907, respectively (**Figure 1**).

### Each Phenotype Has Distinct Immunogenetic Features

**Figure 2:**
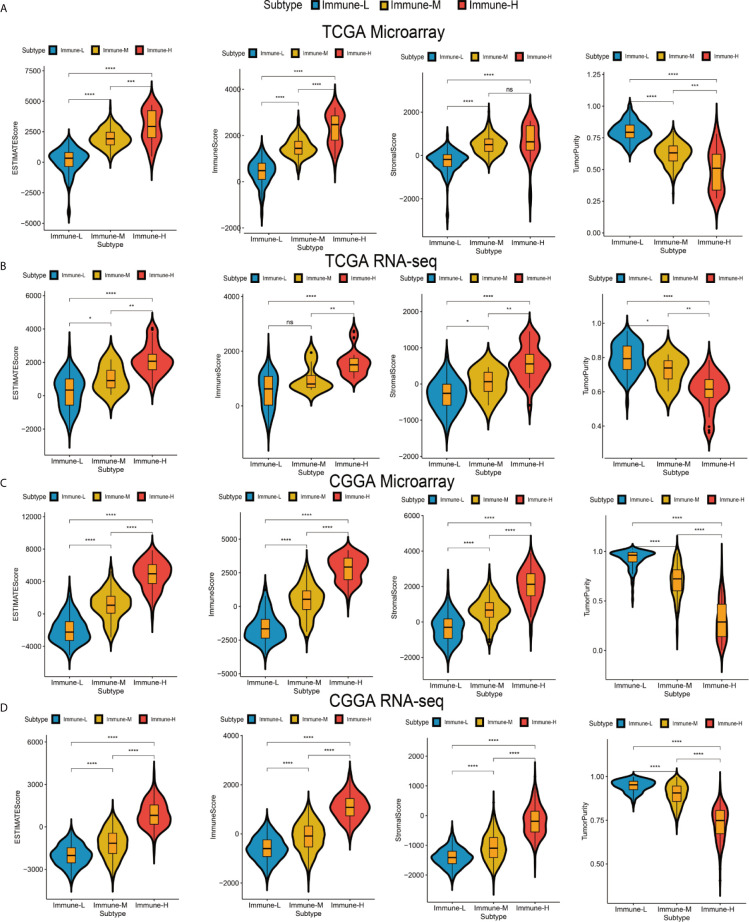
Differences among immune phenotypes in terms of four glioma immune microenvironment signatures. **(A–D)** Violin plots comparing the ESTIMATE, immune and stromal scores and tumor purity among immune phenotypes in the TCGA microarray, TCGA GBM RNA-seq, CGGA microarray, and CGGA RNA-seq cohorts respectively. P values for Wilcoxon test were shown on the top of each violin plot. *P < 0.05; **P < 0.01; ***P < 0.001; ****P < 0.0001. ns, not significant.

Four immune scores were employed. From the ESTIMATE algorithm, the immune-H phenotype was revealed to have a higher ESTIMATE score, immune score and stromal score and a lower tumor purity score than the immune-M and immune-L phenotypes. Statistical comparisons showed that there were significant differences between the immune-H and immune-L phenotypes (Wilcoxon P-value < 0.001) related to these immune signatures (**Figure 2**).

**Figure 3:**
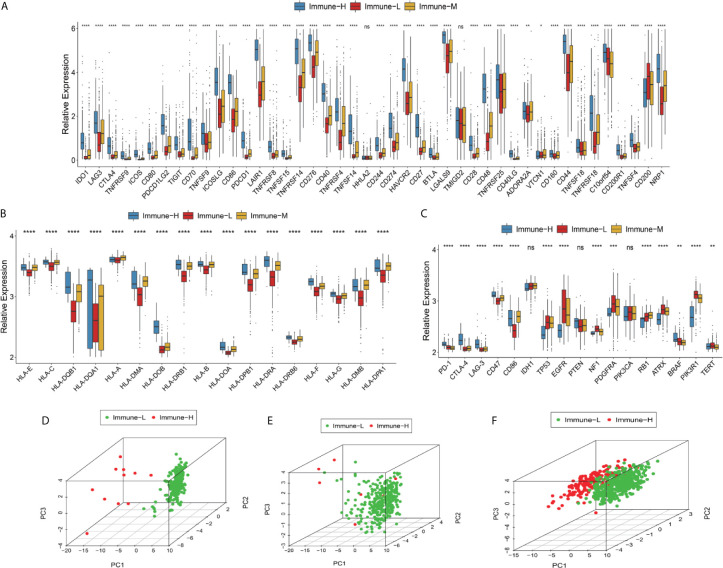
Differences in checkpoints, HLA family and other key biomarkers between the immune phenotypes. **(A)** Expression of checkpoint family biomarkers of each phenotype in the CGGA RNA-seq cohort. **(B)** Expression of HLA family genes of each phenotype in the TCGA microarray cohort. **(C)** Expression of part T cells co-inhibitors checkpoints and key biomarkers relating to glioma biological behavior and pathways in the TCGA microarray cohort. The upper and lower ends of the boxes represented interquartile range of values. The lines in the boxes represented median value, and black dots showed outliers. The asterisks represented the statistical P value (*P < 0.05; **P < 0.01; ***P < 0.001; ***P < 0.0001, ns, not significant). **(D–F)** There was separation between the immune-H and immune-L phenotypes in the TCGA microarray **(D)**, TCGA GBM RNA-seq **(E)** and CGGA RNA-seq cohorts **(F)** according to PCA. PC1, PC2, PC3 represented three dimensions showing differential expression of markers related to immune cell lineage.

Checkpoint biomarkers are involved in tumor subtype classification, prognosis prediction and immunotherapy therapy response evaluation. We observed that most checkpoints were differentially expressed. Such biomarkers were more highly expressed in the immune-H phenotype than in the immune-M and immune-L groups. CD200 was highly expressed in the immune-L phenotype (K-W test P value < 0.001) (**Figure 3A**). HLA genes took important roles in innate immunity and tumor immune microenvironment regulation, these family genes had significantly different expression among phenotypes, with the immune-H group exhibiting significantly higher expression than the other two groups (**Figure 3B**). Furthermore, the immune-L showed higher expression of TP53, EGFR, NF1, PDGFRA, and RB1than immune-H phenotype, which suggested the converged axes of P53, tumor suppressive Rb and MAPK/PI3K were potentially activated in immune-L phenotype. IDH-mutant glioma with ATRX and TERT mutations was always associated with favorable survival (**Figure 3C**). Good separation between the immune-H and immune-L phenotypes was confirmed by PCA (**Figures 3D–F** **)**. Based on the above results, the immune-H phenotype may be more sensitive to classic checkpoint immunotherapy than the others, while the immune-L phenotype was associated with longer survival and better prognosis.

### The Immune-H Phenotype Is Associated With a Poor Prognosis

**Figure 4:**
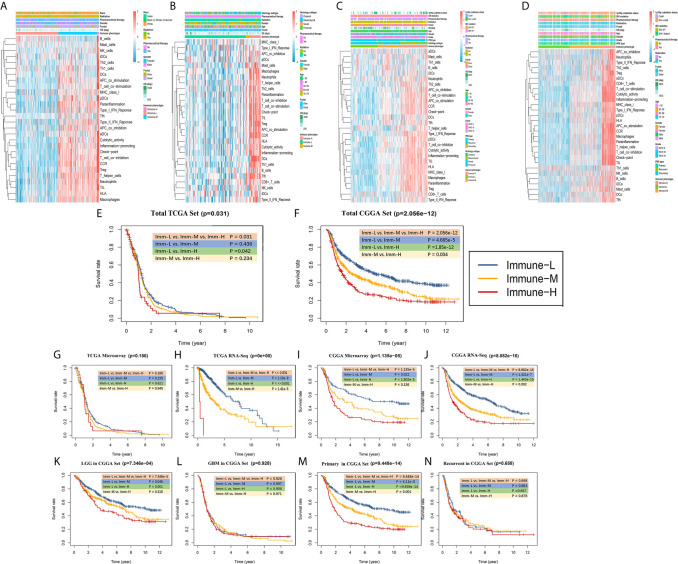
Survival data showing that the immune-H phenotype is associated with a poor prognosis. **(A–D)** Complex heatmaps including ssGSEA results and clinical information from involved TCGA microarray, TCGA GBM RNA-seq, CGGA microarray, and CGGA RNA-seq cohorts. **(E, F)** Survival plots showed immune-H phenotype had poorer survival in all three immune phenotypes in total TCGA (P = 0.031) and CGGA (P = 2.056e-12) datasets. **(G–J)** Survival plots showing prognosis discrepancies among three immune phenotypes in TCGA microarray, TCGA RNA-Seq, CGGA microarray, CGGA RNA-Seq cohorts. **(K–N)** Survival plots for the LGG, GBM, primary glioma and recurrent glioma subpopulations in the total CGGA dataset. The log-rank test P value among three phenotypes and every two phenotypes are marked and shown.

Clinical and molecular features of the immune-specific phenotypes of glioma are displayed in complex heatmaps (**Figures 4A–D**, **Supplementary Online Files 1–4**). Treatment options and histological characteristics seemed to have more prognostic influence, and patients who had received corresponding chemotherapy (including adjuvant temozolomide (TMZ) therapy) or radiotherapy or who had a lower tumor grade and malignancy were observed to have favorable survival. The results are summarized in standardized **Table 1**. In the TCGA (n = 701) (log-rank P-value = 0.031) and CGGA cohorts (n = 1281) (log-rank P-value = 2.056e-12), the immune-H phenotype exhibited unfavorable survival compared with the immune-L phenotype (**Figures 4E, F**). Similar findings were consistent and confirmed in the TCGA RNA-seq (P-value < 0.001), CGGA microarray (P-value = 1.135e-5) and CGGA RNA-seq cohorts (P-value = 8.882e-16), but the results were not significant in the TCGA microarray cohort due to limitations derived from the sample number (P-value = 0.186) (**Figures 4G–J** **).** For subgroup analyses conducted in the CGGA cohort, in the LGG and primary glioma patients, there were significant survival differences between the immune-H, -M and -L phenotypes (log-rank P-value = 7.346e-4; P-value = 9.448e-14, respectively). The prognostic value was not obvious in the GBM (P-value = 0.928) or recurrent subpopulations (P-value = 0.658) (**Figures 4K–N**). These results were contrary to those of previous studies on other cancer types, including breast cancer (38), gastric cancer (39) and head and neck squamous cell cancer (40), which indicated the specificity of the association between tumor immunity and clinical outcomes in glioma, the microenvironment of which is regarded as rather immunosuppressive and refractory. Additionally, intrinsic limitations associated with sample size and variation of ethnicity among the used databases or cohorts should be acknowledged.

**Table 1:** Results of univariable and multivariable analyses on overall survival of glioma patients from multiple cohorts.

	Univariable Cox		Multivariable Cox	
	HR (95% CI)	P-value	HR (95% CI)	P-value
***TCGA microarray* cohort**				
Gender (male vs. female)	1.09 (0.87-1.35)	0.457	1.22 (0.98-1.53)	0.080
Radiation (yes vs. no)	0.13 (0.09-0.18)	< 0.001^*^	0.15 (0.10-0.21)	< 0.001^*^
Chemotherapy (yes vs. no)	0.43 (0.33-0.54)	< 0.001^*^	0.56 (0.43-0.72)	< 0.001^*^
Ethnicity (not Hispanic or Latino vs. Hispanic or Latino)	0.90 (0.46-1.75)	0.750	0.87 (9,44-1.72)	0.685
Race				
White	NA	NA	NA	NA
Asian	0.97 (0.48-1.96)	0.935	1.05 (0.52-2.16)	0.885
Black or African American	0.82 (0.54-1.24)	0.350	0.96 (0.63-1.46)	0.845
Phenotype				
Immune-L	NA	NA	NA	NA
Immune-M	0.82 (0.43-1.56)	0.550	0.68 (0.35-1.30)	0.246
Immune-H	0.94 (0.49-1.79)	0.849	0.81 (0.42-1.55)	0.525
***TCGA GBM RNA-seq* cohort**				
Age (y)				
< 50	NA	NA	NA	NA
50-59	1.26 (0.69-2.31)	0.445	1.37 (0.70-2.68)	0.358
60-69	0.98 (0.56-1.72)	0.944	0.93 (0.48-1.80)	0.831
70-79	1.97 (1.03-3.79)	0.042^*^	2.20 (1.01-4.79)	0.048^*^
Gender (male vs. female)	0.89 (0.57-1.38)	0.599	1.19 (0.72-1.98)	0.497
Radiation (yes vs. no)	0.31 (0.15-0.65)	0.002^*^	0.31 (0.10-0.94)	0.039^*^
Chemotherapy (yes vs. no)	0.34 (0.18-0.66)	0.002^*^	0.76 (0.25-2.28)	0.620
Adjuvant TMZ chemotherapy (yes vs. no)	0.64 (0.41-0.99)	0.050^*^	0.91 (0.53-1.58)	0.746
Histology type				
Proneural	NA	NA	NA	NA
Neural	0.94 (0.49-1.84)	0.866	0.96 (0.45-2.03)	0.907
Classical	0.88 (0.44-1.52)	0.534	1.10 (0.54-2.24)	0.794
Mesenchymal	0.99 (0.56-1.75)	0.964	0.92 (0.45-1.87)	0.814
Phenotype				
Immune-L	NA	NA	NA	NA
Immune-M	0.77 (0.28-2.13)	0.619	0.88 (0.30-2.59)	0.817
Immune-H	1.68 (0.96-2.92)	0.067	2.00 (1.04-3.86)	0.038^*^
***CGGA microarray* cohort**				
Age (y)				
< 50	NA	NA	NA	NA
50-59	2.80 (1.96-4.01)	< 0.001^*^	1.70 (1.13-2.55)	0.011^*^
60-69	2.61 (1.67-4.08)	< 0.001^*^	1.60 (0.99-2.59)	0.055
70-79	16.69 (2.24-)	0.006^*^	6.42 (0.77-53.42)	0.085
Gender (male vs. female)	1.27 (0.94-1.72)	0.125	1.08 (0.78-1.49)	0.640
PRS type				
Primary	NA	NA	NA	NA
Recurrent	1.89 (1.11-3.22)	0.020^*^	2.19 (1.17-4.10)	0.014^*^
Secondary	4.44 (2.25-8.77)	< 0.001^*^	2.83 (1.31-6.14)	0.008^*^
Histology (GBM vs. LGG)	4.44 (3.24-6.09)	< 0.001^*^	4.69 (2.81-7.85)	< 0.001^*^
Grade				
WHO II	NA	NA	NA	NA
WHO III	3.08 (1.94-4.90)	< 0.001^*^	2.77 (1.62-4.71)	< 0.001^*^
WHO IV	6.83 (4.60-10.12)	< 0.001^*^	NA	NA
Radiation (yes vs. no)	0.49 (0.31-0.78)	0.003^*^	0.48 (0.28-0.81)	0.006^*^
Chemotherapy (yes vs. no)	1.57 (1.16-2.14)	0.004^*^	0.83 (0.57-1.20)	0.314
IDH1 status (IDH1 MT vs IDH1 WT)	0.42 (0.31-0.58)	< 0.001^*^	0.88 (0.59-1.31)	0.533
Histology type				
Proneural	NA	NA	NA	NA
Neural	0.80 (0.51-1.27)	0.343	0.95 (0.58-1.56)	0.845
Classical	2.67 (1.50-4.74)	< 0.001^*^	1.15 (0.59-2.25)	0.673
Mesenchymal	2.61 (1.81-3.77)	< 0.001^*^	1.75 (1.05-2.91)	0.031^*^
Phenotype				
Immune-L	NA	NA	NA	NA
Immune-M	1.77 (1.20-2.61)	0.004^*^	1.14 (0.71-1.82)	0.584
Immune-H	2.31 (1.59-3.36)	< 0.001^*^	0.83 (0.48-1.44)	0.512
***CGGA RNA-seq* cohort**				
Age (y)				
< 50	NA	NA	NA	NA
50-59	1.65 (1.33-2.05)	< 0.001^*^	1.11 (0.88-1.39)	0.376
60-69	2.40 (1.85-3.11)	< 0.001^*^	1.26 (0.96-1.67)	0.099
70-79	4.19 (2.53-6.95)	< 0.001^*^	2.35 (1.38-3.98)	0.002^*^
Gender (male vs. female)	1.01 (0.85-1.20)	0.922	1.12 (0.94-1.33)	0.217
PRS type				
Primary	NA	NA	NA	NA
Recurrent	2.23 (1.86-2.67)	< 0.001^*^	2.30 (1.90-2.79)	< 0.001^*^
Secondary	4.37 (2.92-6.54)	< 0.001^*^	3.11 (2.00-4.83)	< 0.001^*^
Histology (GBM vs. LGG)	4.38 (3.66-5.25)	< 0.001^*^	5.85 (4.25-8.06)	< 0.001^*^
Grade				
WHO II	NA	NA	NA	NA
WHO III	2.04 (2.24-3.87)	< 0.001^*^	2.68 (2.00-3.59)	< 0.001^*^
WHO IV	8.33 (6.39-10.85)	< 0.001^*^	NA	NA
Radiation (yes vs. no)	0.97 (0.77-1.23)	0.817	0.83 (0.64-1.06)	0.130
Chemotherapy (yes vs. no)	1.59 (1.30-1.94)	< 0.001^*^	0.72 (0.57-0.89)	0.003^*^
IDH1 status (IDH1 MT vs IDH1 WT)	0.32 (0.27-0.38)	< 0.001^*^	0.50 (0.40-0.62)	< 0.001^*^
Phenotype				
Immune-L	NA	NA	NA	NA
Immune-M	1.44 (1.18-1.74)	< 0.001^*^	1.04 (0.86-1.27)	0.685
Immune-H	1.94 (1.54-2.44)	< 0.001^*^	0.94 (0.73-1.20)	0.607

^*^represents the statistical test is significant (P < 0.05).

HR, hazard ratio; TMZ, temozolomide; LGG, low grade glioma; GBM, glioblastoma; IDH1 MT, IDH1 mutant type; IDH1 WT, IDH1 wild type; NA, not available.

### Infiltrating Immune Cell Fractions and Correlations

**Figure 5:**
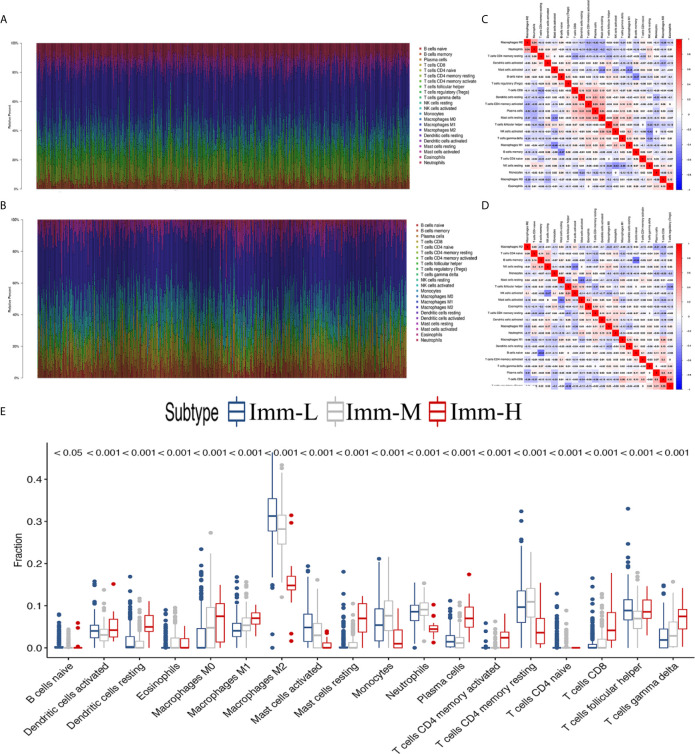
The landscape of immune cell infiltration in the glioma microenvironment. **(A, B)** The proportions of 22 infiltrating immune cells in the glioma microenvironment in the TCGA microarray and CGGA RNA-seq cohorts respectively. **(C, D)** Correlation heatmaps of the TCGA microarray and CGGA RNA-seq cohorts respectively. **(E)** Immune cell infiltration level of glioma microenvironment among immune phenotypes in the TCGA microarray cohort based on the CIBERSORT algorithm.

Through the CIBERSORT algorithm, M2, M1, and M0 macrophages, monocytes, DCs, and subsets of B and T cells (CD4+ and CD8+) were distinguished in the glioma microenvironment (**Figures 5A, B**). The results derived from ESTIMATE and CIBERSORT classified the glioma samples into three immune phenotypes, which had similar characteristics to those of the previously identified phenotypes. Correlations between each type of immune cell illustrated that the most negative correlations were found among M0 macrophages, monocytes, M2 macrophages, DCs (activated and resting) and helper T cells. These results suggested that myeloid cells highly participated in the immunosuppressive glioma microenvironment (**Figures 5C, D** **)**. Comparing the proportion of infiltrating immune cells, the immune-H phenotype was revealed to have higher proportions of all analyzed immune cells, except M2 macrophages, activated mast cells, monocytes, neutrophils and resting memory CD4+ T cells (**Figure 5E**).

### Construction of the Exhausted CD8+ T Cell Signature

**Figure 6:**
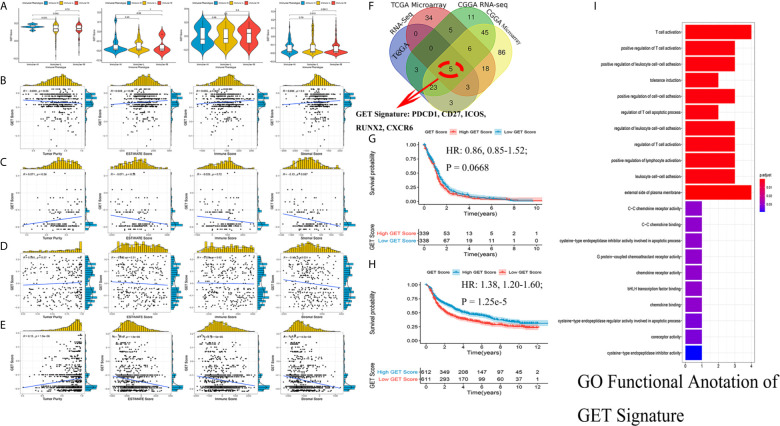
Detection of a gene expression signature of exhausted CD8+ T cells in glioma. **(A)** Comparisons of GET Score among classified immune phenotypes in TCGA microarray, TCGA RNA-Seq, CGGA microarray, CGGA RNA-Seq four cohorts. **(B–E)** The correlation between GET Score and Tumor Purity, ESTIMATE Score, Immune Score and Stromal Score in above four cohorts respectively. **(F)** Venn diagram exhibited the five selected genes termed as GET Signature (PDCD1, CD27, ICOS, RUNX2, CXCR6). **(G, H)** Comparison of the prognosis of high GET Score and low GET Score group in total TCGA and CGGA datasets. The cut-off value was defined as the median GET Score of all involved samples. **(I)** Functional enrichment of GO terms relating to the GET Signature.

Exhausted CD8+ T cell levels were recognized to be uniquely regulated by distinct PD-1 upregulation. With transcriptional profiles of CD8+ T lymphocytes and upregulated PD-1-positive genes captured from previous studies (24, 25), correlation analyses were carried out in the involved datasets, in which five genes meeting the selection criteria were selected and termed GET signature. The GET signature included PDCD1, CD27, ICOS, RUNX2, and CXCR6, which are closely linked to T cell dysfunction and coregulation (**Figure 6F**). The GET score of each tumor sample was established with the ssGSEA method. To quantitatively illustrate the status of exhausted CD8+ T cells in each immune phenotype, we compared the distribution of the GET score in different phenotypes. We did not observe significant differences in the GET score between the immune-L, -M and -H phenotypes (**Figure 6A**, **Supplementary Online File 5**). Correlations between the defined GET score and immune score, ESTIMATE score, stromal score and tumor purity were assessed, and no tight correlation was found among these signatures (**Figures 6B–E** **)**. The results from the TCGA microarray dataset seemed to vary slightly from the results in other datasets, and the lack of CD8+ T cells in the glioma microenvironment and the failure of immune surveillance against tumor cells were likely causes of these effects (41). Patients with a higher GET score in the total CGGA cohort had a more favorable prognosis than those with a lower GET score (HR: 1.38, 1.20-1.60; P-value = 1.25e-5), and the results were not significant in the total TCGA cohort (**Figures 6G, H** **) (** **Supplementary Online File 6** **)**. Confirmatively, nearly all of the constructed GET signatures were mainly related to inflammatory components, lymphocyte functions and immune cell signaling (**Figure 6I** **)**. To date, crosstalk between the GET signature and other molecular and clinicopathological factors is being warmly discussed in glioma, and more evidence is needed in the future.

### Functional Enrichment Analysis of Phenotype-Associated Genes

**Figure 7:**
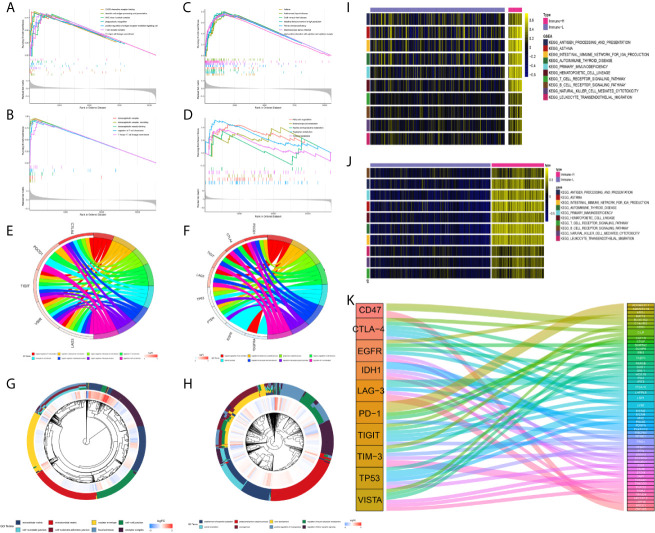
Comprehensive functional analysis relating to the immune phenotypes. **(A, B)** GSEA of GO terms of metagenes co-expressed in the immune-H and immune-L phenotypes in the TCGA microarray and CGGA RNA-seq cohorts. **(C, D)** GSEA of pathways of metagenes co-expressed in the immune-H and immune-L phenotypes in the TCGA microarray and CGGA RNA-seq cohorts. **(E–H)** GO chord plots showing correlation and clusters of PDCD1, CTLA-4, TIGIT, LAG3, TP53, VSIR, PTEN, EGFR, PDGFRA checkpoints. **(I, J)** Variants in pathway categories demonstrated by GSVA relating to immune-H and immune-L phenotypes in TCGA microarray and CGGA RNA-seq cohorts. **(K)** The Sankey diagram showed multiple correlations between CD47, CTLA-4, EGFR, IDH1, LAG-3, PD-1, TIGIT, TIM-3, TP53, VISTA and their top-ranked correlated genes in glioma.

In subsequent functional analyses of the biological processes of the identified microenvironment-related genes in the immune phenotypes, metagenes chosen as classifier gene sets for the immune-H over the immune-L phenotype in GSEA were significantly enriched in immune-related GO terms such as dendritic cell antigen processing and presentation, immunoglobulin processes, regulation of T cell chemotaxis, and T helper cell lineage (P-value and Benjamini P-value < 0.05); metagenes were significantly enriched in pathways related to immune-related graft-versus-host disease, the hematopoietic cell lineage, and the IL-17 signaling pathway (P-value and Benjamini P-value < 0.05) according to pathway GSEA (**Figures 7A–D**). Bubble plots can be found in **Appendix Figure A1**. The cluster maps display whole gene clusters and enriched GO terms, and the GO chord plots show relevant GO terms for the classic PD1/PDCD1, CTLA-4, TIGIT, VISTA/VISR, and LAG-3 molecules (**Figures 7E–H**). GSVA showed enrichment discrepancies in several immune-related pathways, including antigen processing, primary immunodeficiency, the B/T cell receptor signaling pathway, NK cell cytotoxicity, and leukocyte transendothelial migration (**Figures 7I, J** **)**. The Sankey diagram shows the links between checkpoint molecules and their correlated genes in glioma (**Figure 7K**).

### Genomic Alterations, Tumor Mutation Burden, and Histological Characteristics

**Figure 8:**
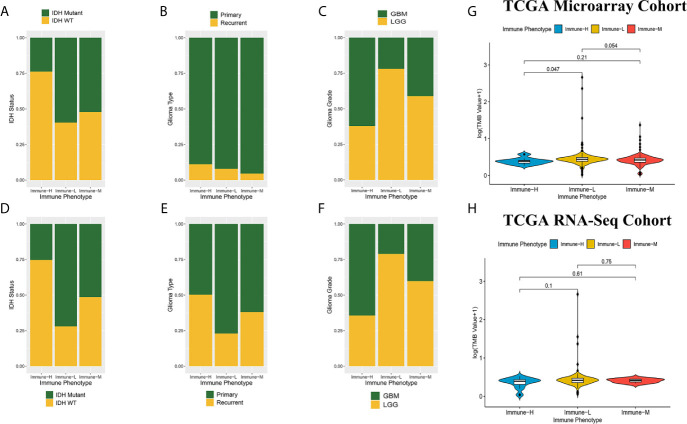
Comparison on IDH status, glioma type, grade and tumor mutation burden among immune phenotypes. **(A)** Proportion of IDH-mutant and IDH-wild type glioma in three phenotypes in CGGA microarray cohort. **(B)** Proportion of primary and recurrent glioma in three phenotypes in CGGA microarray cohort. **(C)** Proportion of LGG and GBM in three phenotypes in CGGA microarray cohort. **(D)** Proportion of IDH-mutant and IDH-wild type glioma in three phenotypes in CGGA RNA-seq cohort. **(E)** Proportion of primary and recurrent glioma in three phenotypes in CGGA RNA-seq cohort. **(F)** Proportion of LGG and GBM in three phenotypes in CGGA RNA-seq cohort. **(G)** Violin plot showing comparison of TMB based on immune-phenotypes in TCGA microarray cohort. **(H)** Violin plot showing comparison of TMB based on immune-phenotypes in TCGA RNA-seq cohort. LGG, low grade glioma; GBM, glioblastoma.

Compared with other immune phenotypes, immune-L was found to have a higher proportion of IDH-mutant patients (**Figures 8A, D** **)**; the immune-H phenotype seemed to have a higher proportion of recurrent glioma but a lower rate of primary patients (**Figures 8B, E** **)**; more GBM samples existed in immune-H, and more LGG was associated with the immune-L phenotype (**Figures 8C, F** **)**. Detailed data are presented in **Table 2**. Surprisingly, no obviously significant discrepancies in TMB were found between the immune-H and immune-L phenotypes in the TCGA microarray cohort (P = 0.047, median log_2_(TMB), 0.385 vs. 0.464) and TCGA RNA-seq cohort (P = 0.100, median log_2_(TMB), 0.357 vs. 0.447) (**Figures 8G, H**, **Supplementary Online File 7**).

**Table 2:** Distribution of IDH status, type and grade of glioma among immune phenotypes in CGGA dataset.

		Immune-L Phenotype		Immune-M Phenotype		Immune-H Phenotype		Chi-square test ^(1)^
**CGGA RNA-seq cohort**	IDH Status	IDH MT(%)	280 (72.0)	IDH MT(%)	203 (51.4)	IDH MT(%)	45 (25.3)	χ2 = 110.855; P < 0.001
		IDH WT (%)	109 (28.0)	IDH WT (%)	192 (48.6)	IDH WT (%)	133 (74.7)	
	Glioma Type	Primary (%)	314 (77.0)	Primary (%)	249 (61.9)	Primary (%)	85 (49.7)	χ2 = 45.058; P < 0.001
		Recurrent (%)	94 (23.0)	Recurrent (%)	153 (38.1)	Recurrent (%)	86 (50.3)	
	Glioma Grade	LGG (%)	322 (78.9)	LGG (%)	240 (59.9)	LGG (%)	61 (35.7)	χ2 = 101.384; P < 0.001
		GBM (%)	86 (21.1)	GBM (%)	161 (40.1)	GBM (%)	110 (64.3)	
**CGGA microarray cohort**	IDH Status	IDH MT(%)	62 (59.6)	IDH MT(%)	47 (52.2)	IDH MT(%)	25 (23.8)	χ2 = 29.941; P < 0.001
		IDH WT (%)	42 (40.4)	IDH WT (%)	43 (47.8)	IDH WT (%)	80 (76.2)	
	Glioma Type	Primary (%)	92 (91.1)	Primary (%)	83 (95.4)	Primary (%)	88 (88.9)	χ2 = 2.625; P = 0.269
		Recurrent (%)	9 (8.9)	Recurrent (%)	4 (4.6)	Recurrent (%)	11 (11.1)	
	Glioma Grade	LGG (%)	82 (78.1)	LGG (%)	53 (58.9)	LGG (%)	39 (37.9)	χ2 = 34.592; P < 0.001
		GBM (%)	23 (21.9)	GBM (%)	38 (42.2)	GBM (%)	64 (62.1)	

^(1)^Chi-square test was conducted to compare these differences between immune phenotypes.

IDH MT, IDH Mutant; IDH WT, IDH Wild Type; LGG, low grade glioma; GBM, glioblastoma.

**Figure 9:**
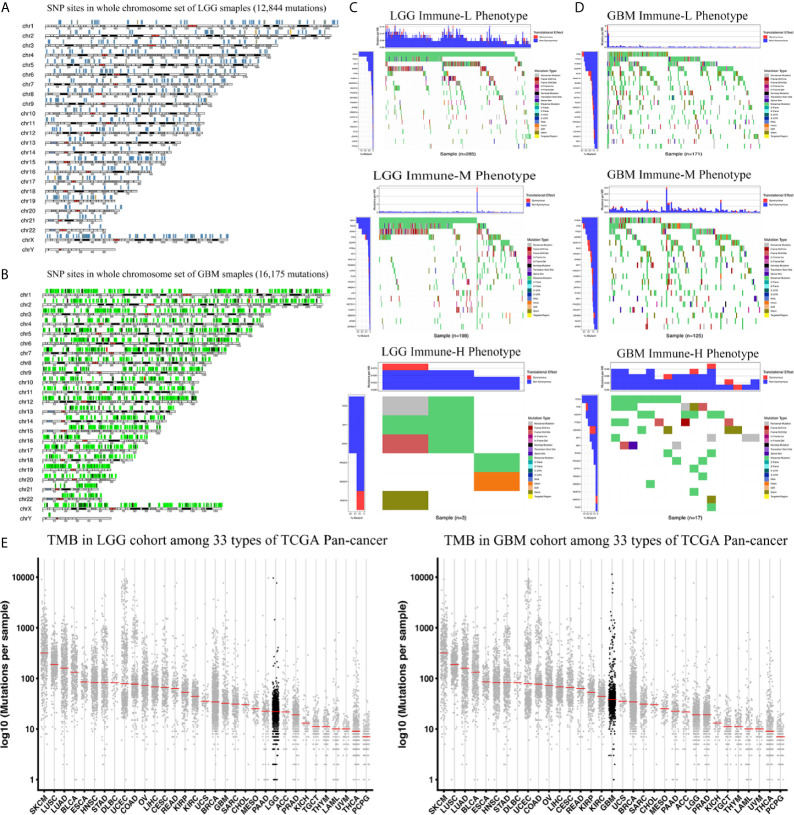
Waterfall plots of genomic alternations associated with glioma immune phenotypes. **(A, B)** Recurrent SNP sites of LGG and GBM in chromosome models. Red and orange marked high-mutant SNP, navy and green marked low-mutant SNP. **(C)** The waterfall plots summarize the genomic alternations including somatic mutations and single nucleotide polymorphism in LGG of immune-L, immune-M and immune-H phenotypes respectively. **(D)** The waterfall plots summarize the genomic alternations in GBM of immune-L, immune-M and immune-H phenotypes respectively. **(E)** Scatter plots show tumor mutation burden of LGG and GBM among 33 types of Pan-cancer respectively. LGG, low grade glioma; GBM, glioblastoma.

We analyzed the distribution differences of somatic mutations and SNPs among the immune phenotypes using data from the TCGA project. **Figures 9A, B** displays recurrent SNP sites (N > 5) in chromosome models in LGG and GBM. Sites marked by orange and red are high-mutant SNP sites, while those marked by navy and green are low-mutant SNP sites. Major mutant genes and mutation types were different among immune phenotypes in combination with glioma grade (**Figures 9C** **, D**). In addition, GBM presented more extensive TMB than LGG, with details in the left bar plots and scatter plots in **Figure 9E**.

### Phenotypes Predicting Response to Antitumor Drugs and Peritumor Edema

**Figure 10:**
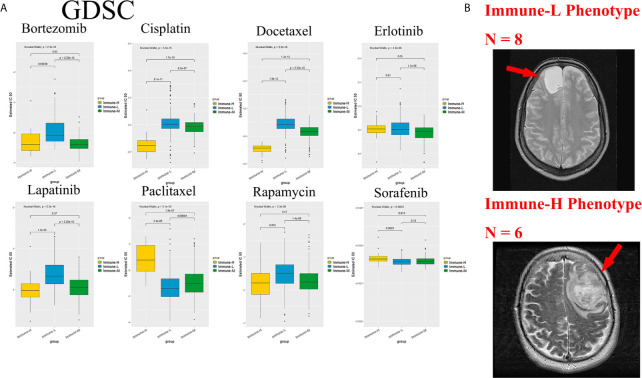
Role of phenotype in predicting anti-tumor drugs response and peri-tumoral edema. **(A)** The immune-L phenotype was more sensitive to bortezomib (P < 2.2e-16), cisplatin (P = 5.3e-15), docetaxel (P < 2.2e-16), lapatinib (P < 2.2e-16), rapamycin (P = 3.3e-8); the immune-H phenotype was more sensitive to paclitaxel (P = 3.1e-10) and sorafenib (P = 0.0053). **(B)** Representative images of the differences in the extent of peri-tumoral edema in TCGA cohort patients. Immune-H phenotype significantly possessed more-severe edema than immune-L.

Chemotherapy and targeted therapy are both standard treatments for glioma. The response to commonly used antitumor drugs was evaluated among three immune phenotypes. The expected model using the GDSC dataset was trained by ridge regression, and the level of prediction accuracy was evaluated by 10-fold cross-validation. The treatment-related IC50 for each tumor sample in TCGA was properly estimated based on a predictive model of these drugs. There were significant differences in the response to several drugs, and the immune-L phenotype was more sensitive to bortezomib (K-W P < 2.2e-16), cisplatin (P = 5.3e-15), docetaxel (P < 2.2e-16), lapatinib (P < 2.2e-16), and rapamycin (P = 3.3e-8); however, the immune-H phenotype was more sensitive to paclitaxel (P = 3.1e-10) and sorafenib (P = 0.0053) (**Figure 10A**).

**Figure 11:**
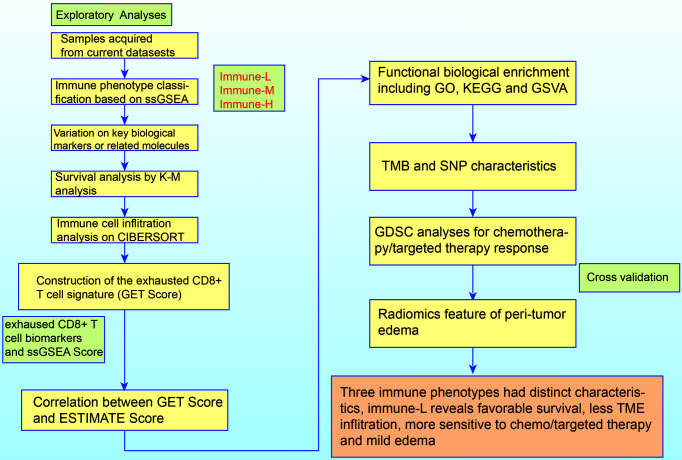
The logic flow chart of current study.

As a marker of inflammation, edema is a common pathophysiological entity surrounding gliomas. Herein, we compared the edema differences between the immune-L and immune-H phenotypes to assess the correlations. It was noted that immune-H phenotype gliomas displayed more severe edema than immune-L phenotype gliomas (**Figure 10B**). The present results suggested that peritumoral edema is also a probable marker to reflect the variations between immune phenotypes. The analysis process used in this study is shown as a flow chart in **Figure 11**.

## Discussion

Immunotherapy has been confirmed to be effective in other types of cancers except glioma, as glioma features a relatively immune-privileged microenvironment. With the aim of elucidating the immunosuppressive mechanism, in this research, we enrolled 2,466 glioma samples from 6 datasets and classified these samples into 3 immune phenotypes with distinct immunogenetic features. The immune-H phenotype has higher immune cell lineage infiltration and higher ESTIMATE, immune and stromal scores than the immune-L and immune-M phenotypes. Most HLA family genes and checkpoint molecules were significantly highly expressed in the immune-H phenotype; otherwise, some specific genes were highly expressed. Overall, patients with the immune-H phenotype will have a poor prognosis compared with those with the immune-L phenotypes, but this result was limited by the sample size. A five-gene GET signature including PDCD1, CD27, ICOS, RUNX2, and CXCR6 was established, and no significant differences in the GET score between immune phenotypes were observed. Patients with a high GET score seemed to have a better prognosis. A response difference was noticed among the phenotypes to several antitumor drugs. Immune-H was observed to have more severe peritumor edema than immune-L in representative T2 images.

Survival differences among the classified immune phenotypes of glioma were in contrast to those of some other cancer types reported previously, such as triple-negative breast cancer (38), gastric cancer (39), and head and neck squamous cancer (40). A potential reason is that the inflammatory microenvironment upregulated the tumor progressive nature and deteriorated glioma invasion and development (41, 42). The success of immunomodulatory therapy is widespread among diverse cancer types, which stimulates our interest in characterizing TME immune cell infiltration in glioma. The immune-H phenotype may be involved in immunosuppressive activities, including immunosuppressive checkpoints (**Table 3**), expression of tumor-supportive macrophage chemotactic and polarizing molecules and immune-suppressive pathway signaling (the IL-10 signaling pathway). The IL-10 pathway downregulates DC activation and IL-12 production and inhibits the cytotoxic T cell response during chemotherapy. Macrophage activation is also suppressed by IL-10 to inhibit the immune response (43). Importantly, there is large heterogeneity in the TME of different glioma genetic subtypes, and enriched tumor-associated macrophages (TAMs) participate in the promotion of glioma invasion, angiogenesis, tumor metastasis and immune suppression through intracellular and extracellular mediators (44). Glioma with IDH mutation status was shown to have low levels of infiltrating T cells and a higher ratio of TAMs derived from microglia (45). Although TAMs have distinct genetic profiles involving canonical M1 (antitumorigenic) and canonical M2 (protumorigenic) polarization, they show increased anti-PD-1 resistance-associated genes and predict poor survival (46, 47). Additionally, immunosuppressive chemokines/cytokines in the TME released by the tumor itself, such as through the TGF-β pathway, also block antitumor immunity activation (48). TIM-3 (T cell immunoglobulin mucin receptor 3) has an immunosuppressive effect in glioma, which may be due to the unique presence of TIM-3+ Tregs in tumor tissue (49). Furthermore, TIM-3 does not contain any immunoreceptor tyrosine-based inhibition motifs (ITIMs), which are necessary for avoiding major deficiencies in immunotherapy (50). VISTA (V-type immunoglobulin domain-containing suppressor of T cell activation) is a newly found checkpoint that restricts T cell activation by shaping the naive CD4+ T cell compartment (51). Therapeutics targeting VISTA curb the development of graft-versus-host disease and promote the death of naive CD4+ T cells; thus, VISTA can be regarded as a distinctive immunotherapy molecule (51, 52). Indeed, growing evidence suggests that dysfunctional CD8+ T cells incorporate heterogeneous subpopulations such as progenitor and terminally exhausted cells, and discrete functions in immunotherapy or the microenvironment need to be better elucidated (53). Clinical trials regarding Checkmate 143 (NCT02017717), Checkmate 498 (NCT02617589), and Checkmate 548 (NCT02667587) did not suggest a profound survival benefit from immunotherapy in glioma/GBM, with only some clinical advantages reported in some case reports; indeed, GBM typically has a relatively low mutational load and a paucity of T cell infiltration compared with other cancers (12, 54).

**Table 3:** Summary of the molecular and biological functions of T cell costimulatory molecules.

Molecular marker	Aliase(s)	Ligand(s)	Receptor expression pattern	Biological function	Molecular function
**Coinhibitory**					
PD-1	PDCD1, CD279, SLEB2, hPD-1	PD-L1, PD-L2	Activated T cells, NK cells, NKT cells, B cells, macrophages, subsets of DCs	Negative T cells costimulation (primarily in periphery), attenuate peripheral activity, preserve T-cell function in the context of chronic antigen	Inhibition of proximal TCR signaling, attenuate CD28 signaling
CTLA-4	CD152, ALPS5, CELIAC3, GRD4	B7-1 (CD80), B7-2 (CD86)	Activated T cells, Tregs	Negative T-cell costimulation (primarily at priming); prevent tonic signaling, attenuate high-affinity clones	Competitive inhibition of CD28 costimulation (binding to B7-1 and B7-2)
PD-L1	CD274, PDCD1L1, B7-H, B7H1	PD-1, B7-1 (CD80)	Monocytes, macrophages, mast cells, inducible in DCs, T cells, B cells, NK cells	Attenuate T cells activity in inflamed peripheral tissues	PD-1 ligation; cell-intrinsic mechanism unclear
LAG-3	CD223, Ly66	MHC-II, LSECtin	Activated CD4+ and CD8+ T cells, NK cells, Tregs	Negative regulator of T cells expansion; control T cells homeostasis; DCs activation	Competitive binding to MHC-II; proximal LSECtin mechanism unclear
TIM-3	HAVCR2, CD366, KIM-3, SPTCL, TIMD-3	Galectin-9, PtdSer, HMGB1, CEACAM-1	Th1 CD4+ and Tc1 CD8+, Tregs, DCs, NK cells, monocytes	Negative regulation of Type immunity; preserve peripheral tolerance	Negative regulation of proximal TCR components; differences between ligands unknown
TIGIT	VSIG9, VSTM3, WUCAM	PVR (CD155), PVRL2 (CD112)	CD4+ and CD8+ T cells, Tregs, TFH, NK cells	Inhibition of T cells activity; DC tolerization	Competitive inhibition of DNAM1 (CD226) costimulation (binding of PVR), binding of DNAM1 in cis; cell-intrinsic ITIM-negative signaling
VISTA	VSIR, B7-H5, B7H5, C10orf54, PD-1H	Counterreceptor unknown	T cells and activated Tregs, myeloid cells, mature APCs	Negative regulation of T cells activity; suppression of CD4+ T cells, shaping naive CD4+ T cells compartment	Increase threshold for TCR signaling, induce FOXP3 synthesis; proximal signaling unknown
**Costimulatory**					
ICOS	AILIM, CCLP, CRP-1	ICOSL	Activated T cells, B cells, ILC2	Positive costimulation; Type I and II immunity; Tregs maintenance; TFH differentiation	p50 PI3K recruitment (AKT signaling); enhance calcium signaling (PLCγ)
OX40	TNFRSF4, ACT35, CD134, TXGP1L	OX40L	Activated T cells, Tregs, NK cells, NKT cells, neutrophils	Sustain and enhance CD4+ T cell immunity; role in CD8+ T cells and Tregs	Regulation of BCL2/XL (survival); enhance PI3K/AKT signaling
GITR	TNFRSF18, AITR, CD357, ENERGEN, GITR	GITRL	Activated T cells, Tregs, B cells, NK cells, macrophages	Attenuate Tregs; costimulation of activated T cells, NK cell activation	Signal through TRAF5
CD137	TNFRSF9, 4-1BB, CDw137, ILA	4-1BBL (CD137L)	Activated T cells, Tregs, NK cells, monocytes, DCs, B cells	Positive T cells costimulation; DC activation	Signal through TRAF1, TRAF2
CD40	TNFRSF5, Bp50, CDW40, p50	CD40L	APCs, B cells, monocytes, non hematopoietic cells (e.g., fibroblasts, endothelial cells)	APC licensing	Signal through TRAF2, 3, 5, 6; TRAF-independent mechanisms unclear
CD27	TNFRSF7, S152, LPFS2, Tp55	CD70	CD4+ and CD8+ T cells, B cells, NK cells	Lymphocyte and NK cell costimulation; generation of T-cell memory	Signal through TRAF2, TRAF5

A summary of the ligands, immune-related expression pattern, biological function, and molecular mechanisms is reviewed for selected costimulatory and coinhibitory receptors. Molecular functions (i.e., downstream signaling) reflect predominant currently known mechanisms, but additional mechanisms are likely to contribute significantly.

NK, natural killer; NKT, natural killer T cell; TFH, T follicular helper; TRAF, tumor necrosis factor receptor–associated factors; DC, dendritic cell.

Similar to other studies, Chen and his colleagues (55) used ssGSEA to identify the immune microenvironment of glioma, and they did not classify glioma samples into immune phenotypes or detect the corresponding microenvironmental features of the phenotypes; however, they detected eight glioma microenvironment-associated genes, CCDC109B, EMP3, ANXA2, CLIC1, TIMP1, VIM, LGALS1, and RBMS1, and constructed a prognostic model with them through integrative omics data points. They validated the immunosuppression of LGALS1 in *in vitro* experiments. Our findings based on large genomic data help characterize the glioma microenvironment and understand tumor immune complexity. The ESTIMATE, immune, stromal, and tumor purity scores can be used properly in both basic and translational medicine to help identify glioma subtypes. Work investigating the immunosuppressive mechanisms of glioma implies that microenvironments lacking T cells feature immunosuppressive biological processes carried out by a series of immune cells; more knowledge of immune cell infiltration will inform strategies to remodel the immunosuppressive microenvironment and will aid the identification of more therapeutic targets.

Patients with the immune-H phenotype were more prone to developing a poor prognosis compared with others; thus, we may properly predict the prognosis of glioma patients with immune phenotypes. Our findings also suggest that immunotherapy will be effective in immune-H patients, who are more sensitive to checkpoint-related immunotherapy (56). Recent evidence showed that samples with high TMB could exhibit a durable response to PD-1/PD-L1 immunotherapy (57), and current findings indirectly confirmed the value of TMB in predicting immunotherapeutic outcomes of established immune phenotypes. Translational research indicated that a high TMB status may yield a long-term response and durable survival benefit (58). The presented results provide a novel perspective on immune signatures in the genetic TMB, the microenvironment and roles in immune checkpoint blockade treatment and inspired the exploration of fresh neoepitopes. Immune phenotype classification highlights the importance of individualized treatments and provides potential methods to be used in further clinical trials related to glioma immunotherapy. We believe that with the current Pan-Cancer Analysis of Whole Genomes (PCAWG) project involving classic glioma microenvironment biomarkers (i.e., IDH1), researchers will identify more specialized features of cancer immune genomes (59).

## Conclusions

Glioma samples can potentially be classified into “immune-H”, “immune-M” and “immune-L” phenotypes, which exhibit distinct immunogenetic features. The immune-H phenotype is associated with higher ESTIMATE, immune and stromal scores but poorer survival than the immune-L phenotype. HLA and checkpoint family genes are relatively highly expressed in patients with the immune-H phenotype. The GET signature cannot effectively reveal the discrepancies among immune phenotypes, and aggressive peritumor edema was displayed in immune-H compared with immune-L phenotypes. Our immunogenetic pipeline characterizes the glioma microenvironment and properly identifies patients who are more sensitive to chemo/targeted therapy and are likely to have better survival. These results possibly facilitate new therapeutic development and advance precision oncology, limited by the observational nature, the experimental profile should be highlighted in the future.

## Data Availability Statement

Publicly available datasets were analyzed in this study. The original contributions presented in the study are included in the article/**Supplementary Material**. Further inquiries can be directed to the corresponding authors.

## Author Contributions

All authors designed and conducted this review. BZ, YKW, and YaW, wrote the paper. YKW helped the study design. CD and YuW revised the statistical methodology. YuW and WM had primary responsibility for the final content. All authors contributed to the article and approved the submitted version. Notably, YuW and WM equally share the corresponding authorship.

## Funding

The work is supported by Chinese Academy of Medical Sciences Innovation Fund for Medical Sciences, number of grants (2016-I2M-2-001) and Beijing Municipal Natural Science Foundation, number of grants (7202150, 19JCZDJC64200(Z)).

## Conflict of Interest

The authors declare that the research was conducted in the absence of any commercial or financial relationships that could be construed as a potential conflict of interest.

## Supplementary Material

Supplementary Figure 1 GO bubble plots of metagenes in Immune-H over immune-L phenotypes. **(A–C)** Bubble plots for TCGA microarray, TCGA GBM RNA-seq, CGGA RNA-seq databases.

## Abbreviations

CNS, central nervous system; PI3K, phosphatidylinositol-3-kinases; Akt, protein-serine-threonine kinase; FAK, focal adhesion kinase; IGF, insulin like growth factor; STAT3, signal transducer and activator of transcription; HIF-1α, hypoxia inducible factor-1α; IL-6, interleukin-6; TGF-β, transforming growth factor-β; PD-1, programmed death 1; PD-L1, programmed death-ligand 1; CTLA-4, cytotoxic T-lymphocyte-associated protein 4; IDH1, isocitrate dehydrogenase 1; MGMT, methylguanine methyltransferase; TP53, tumour protein 53; TERT, telomerase reverse transcriptase; mAb, monoclonal antibody; OS, overall survival; NSCLC, non-small cell lung cancer; APC, antigen-presenting cell; GBM, glioblastoma; LGG, low grade glioma; NK cell, natural killer cell; HLA, human leukocyte antigen; TNFRSF6, tumour necrosis factor receptor superfamily member 6; DC, dendritic cell; TCGA, The Cancer Genome Atlas; CGGA, Chinese Glioma Genome Atlas; GEO, Gene Expression Omnibus; SNP, single nucleotide polymorphism; ssGSEA, Single-sample Gene Set Enrichment Analysis; GO, Gene Ontology; KEGG, Kyoto Encyclopedia of Genes and Genomes; GSEA, Gene Set Enrichment Analysis; GSVA, Gene set variation analysis; FDR, false discover rate; PCA, principal component analysis; TMZ, temozolomide; TMB, tumor mutation burden; GET, exhausted CD8+ T cells; TAMs, tumor-associated macrophages; TIM-3, T cell immunoglobulin and mucin domain-containing protein 3; LAG-3, lymphocyte activation gene-3; TIGIT, T cell immunoreceptor with Ig and ITIM domains; VISTA, V-type immunoglobulin domain-containing suppressor of T cell activation; TAM, Tumour-associated macrophages; CHI3L1, Chitinase-3-like protein 1; IL-13Ra2, interleukin-13 receptor α2 chain; VEGFR, Vascular Endothelial Growth Factor Receptor; VEGFA, vascular endothelial growth factor A; PCAWG, Pan-Cancer Analysis of Whole Genomes

## Glossary

CNS: central nervous system
PI3K: phosphatidylinositol-3-kinases
Akt: protein-serine-threonine kinase
FAK: focal adhesion kinase
IGF: insulin like growth factor
STAT3: signal transducer and activator of transcription
HIF-1α: hypoxia inducible factor-1α
IL-6: interleukin-6
TGF-β: transforming growth factor-β
PD-1: programmed death 1
PD-L1: programmed death-ligand 1
CTLA-4: cytotoxic T-lymphocyte-associated protein 4
IDH1: isocitrate dehydrogenase 1
MGMT: methylguanine methyltransferase
TP53: tumour protein 53
TERT: telomerase reverse transcriptase
mAb: monoclonal antibody
OS: overall survival
NSCLC: non-small cell lung cancer
APC: antigen-presenting cell
GBM: glioblastoma
LGG: low grade glioma
NK cell: natural killer cell
HLA: human leukocyte antigen
TNFRSF6: tumour necrosis factor receptor superfamily member 6
DC: dendritic cell
TCGA: The Cancer Genome Atlas
CGGA: Chinese Glioma Genome Atlas
GEO: Gene Expression Omnibus
SNP: single nucleotide polymorphism
ssGSEA: Single-sample Gene Set Enrichment Analysis
GO: Gene Ontology
KEGG: Kyoto Encyclopedia of Genes and Genomes
GSEA: Gene Set Enrichment Analysis
GSVA: Gene set variation analysis
FDR: false discover rate
PCA: principal component analysis
TMZ: temozolomide
TMB: tumor mutation burden
GET: exhausted CD8+ T cells
TAMs: tumor-associated macrophages
TIM-3: T cell immunoglobulin and mucin domain-containing protein 3
LAG-3: lymphocyte activation gene-3
TIGIT: T cell immunoreceptor with Ig and ITIM domains
VISTA: V-type immunoglobulin domain-containing suppressor of T cell activation
TAM: Tumour-associated macrophages
CHI3L1: Chitinase-3-like protein 1
IL-13Ra2: interleukin-13 receptor α2 chain
VEGFR: Vascular Endothelial Growth Factor Receptor
VEGFA: vascular endothelial growth factor A
PCAWG: Pan-Cancer Analysis of Whole Genomes
