# Supplementary figures and images for: Investigation of Genetic Determinants of Glioma Immune Phenotype by Integrative Immunogenomic Scale Analysis
Source: Front Immunol. 2021 Jun 16;12:557994. doi: 10.3389/fimmu.2021.557994 (PMC8242587; doi:10.3389/fimmu.2021.557994)

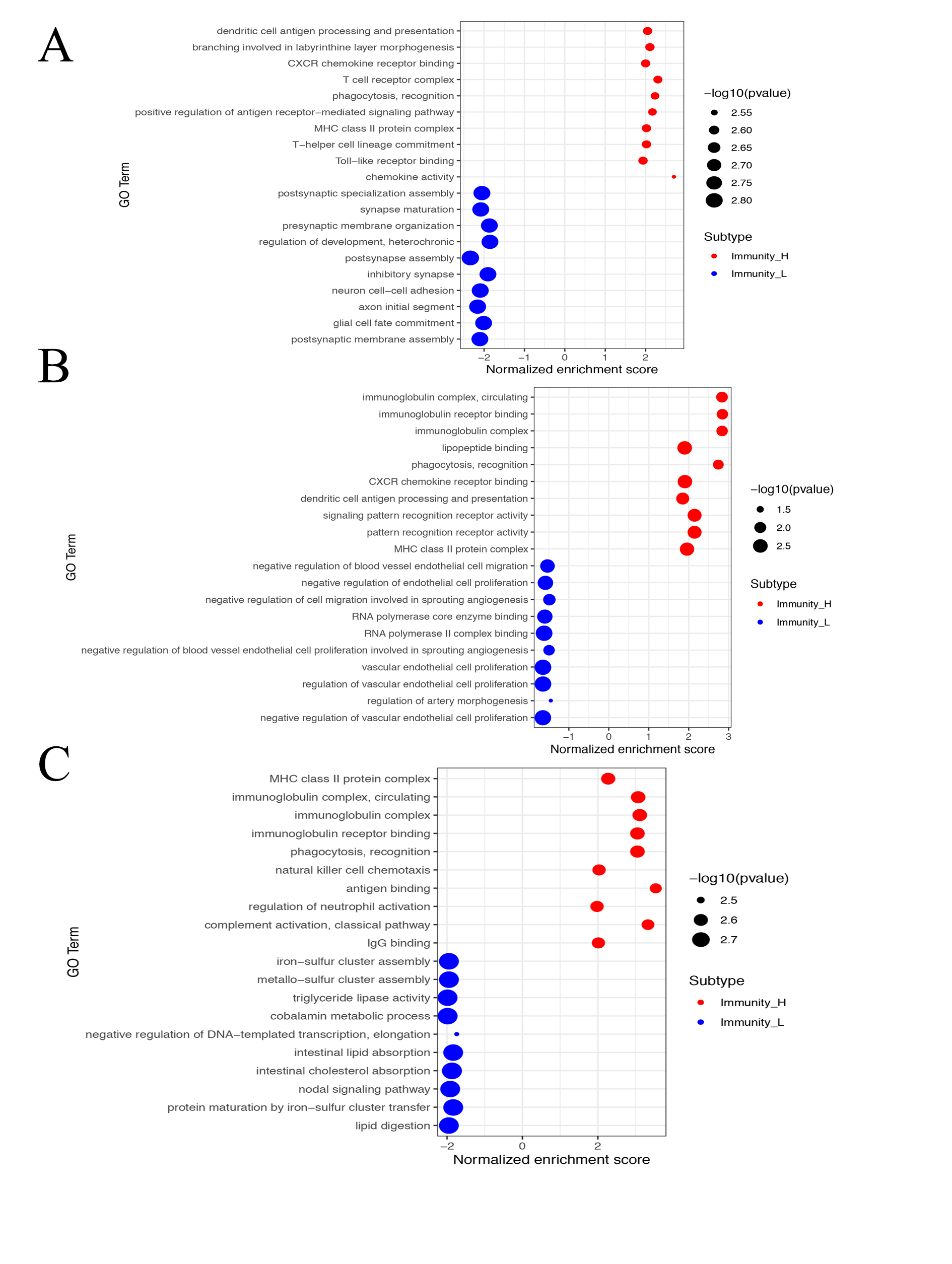

Supplement: Supplementary Figure 1 — GO bubble plots of metagenes in Immune-H over immune-L phenotypes. (A–C) Bubble plots for TCGA microarray, TCGA GBM RNA-seq, CGGA RNA-seq databases. [file Image_1.tif]
