# Supplementary material for: Investigation of Genetic Determinants of Glioma Immune Phenotype by Integrative Immunogenomic Scale Analysis
Source: Front Immunol. 2021 Jun 16;12:557994. doi: 10.3389/fimmu.2021.557994 (PMC8242587; doi:10.3389/fimmu.2021.557994)
Supplement: Supplementary file 2 [file Table_1.docx]

**Supplementary Online File 1. Clinical data for TCGA microarray samples**

| id | OS (day) | Survival status | Gender | Pharmaceutical therapy | Ethnicity | Radiation | Race |
| --- | --- | --- | --- | --- | --- | --- | --- |
| TCGA-02-0001 | 358 | Dead | Female | Yes | Not Hispanic or Latino | Yes | White |
| TCGA-02-0003 | 144 | Dead | Male | Yes | Not Hispanic or Latino | Yes | White |
| TCGA-02-0004 | 345 | Dead | Male | Yes | Not Hispanic or Latino | Yes | White |
| TCGA-02-0007 | 705 | Dead | Female | Yes | Not Hispanic or Latino | Yes | White |
| TCGA-02-0009 | 322 | Dead | Female | No | Not Hispanic or Latino | Yes | White |
| TCGA-02-0010 | 1077 | Dead | Female | Yes | Not Hispanic or Latino | Yes | White |
| TCGA-02-0011 | 630 | Dead | Female | Yes | Hispanic or Latino | Yes | White |
| TCGA-02-0014 | 2512 | Dead | Male | Yes | Not Hispanic or Latino | Yes | White |
| TCGA-02-0015 | 627 | Dead | Male | Yes | Not Hispanic or Latino | Yes | White |
| TCGA-02-0016 | 2648 | Dead | Male | Yes | Not Hispanic or Latino | Yes | White |
| TCGA-02-0021 | 2362 | Dead | Female | Yes | Not Hispanic or Latino | Yes | White |
| TCGA-02-0023 | 612 | Dead | Female | Yes | Not Hispanic or Latino | Yes | White |
| TCGA-02-0024 | 1615 | Dead | Male | Yes | Not Hispanic or Latino | Yes | White |
| TCGA-02-0025 | 1300 | Dead | Male | Yes | Not Hispanic or Latino | Yes | White |
| TCGA-02-0026 | 748 | Dead | Male | Yes | Not Hispanic or Latino | Yes | White |
| TCGA-02-0027 | 370 | Dead | Female | Yes | Not Hispanic or Latino | Yes | White |
| TCGA-02-0028 | 2755 | Dead | Male | Yes | Not Hispanic or Latino | Yes | White |
| TCGA-02-0034 | 430 | Dead | Male | Yes | Not Hispanic or Latino | Yes | White |
| TCGA-02-0038 | 326 | Dead | Female | Yes | Not Hispanic or Latino | Yes | White |
| TCGA-02-0039 | 320 | Dead | Male | No | Not Hispanic or Latino | Yes | White |
| TCGA-02-0043 | 557 | Dead | Female | Yes | Hispanic or Latino | Yes | White |
| TCGA-02-0047 | 448 | Dead | Male | No | Not Hispanic or Latino | Yes | White |
| TCGA-02-0051 | 459 | Dead | Male | No | Not Hispanic or Latino | Yes | White |
| TCGA-02-0054 | 199 | Dead | Female | Yes | Not Hispanic or Latino | Yes | White |
| TCGA-02-0057 | 604 | Dead | Female | Yes | Not Hispanic or Latino | Yes | White |
| TCGA-02-0058 | 254 | Dead | Female | Yes | Not Hispanic or Latino | Yes | White |
| TCGA-02-0059 | 291 | Dead | Female | No | Not Hispanic or Latino | Yes | White |
| TCGA-02-0060 | 183 | Dead | Female | Yes | Not Hispanic or Latino | Yes | White |
| TCGA-02-0064 | 600 | Dead | Male | Yes | Not Hispanic or Latino | Yes | White |
| TCGA-02-0068 | 804 | Dead | Male | Yes | Not Hispanic or Latino | Yes | White |
| TCGA-02-0069 | 873 | Alive | Female | Yes | Not Hispanic or Latino | Yes | Asian |
| TCGA-02-0070 | 762 | Alive | Male | Yes | Not Hispanic or Latino | Yes | White |
| TCGA-02-0071 | 167 | Dead | Male | No | Not Hispanic or Latino | Yes | White |
| TCGA-02-0074 | 310 | Dead | Female | Yes | Not Hispanic or Latino | Yes | Asian |
| TCGA-02-0075 | 634 | Dead | Male | Yes | Not Hispanic or Latino | Yes | White |
| TCGA-02-0079 | 829 | Dead | Male | No | Not Hispanic or Latino | Yes | White |
| TCGA-02-0080 | 2729 | Dead | Male | Yes | Not Hispanic or Latino | Yes | White |
| TCGA-02-0083 | 691 | Dead | Female | Yes | Not Hispanic or Latino | Yes | White |
| TCGA-02-0085 | 1561 | Dead | Female | Yes | Not Hispanic or Latino | Yes | White |
| TCGA-02-0086 | 268 | Dead | Female | Yes | Not Hispanic or Latino | Yes | White |
| TCGA-02-0087 | 1757 | Alive | Female | No | Not Hispanic or Latino | Yes | White |
| TCGA-02-0089 | 516 | Dead | Male | Yes | Not Hispanic or Latino | Yes | White |
| TCGA-02-0099 | 106 | Dead | Male | Yes | Not Hispanic or Latino | Yes | White |
| TCGA-02-0102 | 822 | Dead | Male | Yes | Not Hispanic or Latino | Yes | White |
| TCGA-02-0104 | 1977 | Dead | Female | Yes | Not Hispanic or Latino | Yes | White |
| TCGA-02-0106 | 355 | Dead | Male | Yes | Not Hispanic or Latino | Yes | White |
| TCGA-02-0107 | 537 | Dead | Male | Yes | Not Hispanic or Latino | Yes | White |
| TCGA-02-0111 | 705 | Dead | Male | Yes | Not Hispanic or Latino | Yes | White |
| TCGA-02-0113 | 2818 | Alive | Female | Yes | Not Hispanic or Latino | Yes | White |
| TCGA-02-0114 | 3041 | Dead | Female | Yes | Not Hispanic or Latino | Yes | White |
| TCGA-02-0115 | 476 | Dead | Male | Yes | Not Hispanic or Latino | Yes | Asian |
| TCGA-02-0116 | 1489 | Dead | Male | Yes | Not Hispanic or Latino | Yes | White |
| TCGA-02-0258 | 503 | Dead | Female | No | Not Hispanic or Latino | Yes | Black or African American |
| TCGA-02-0260 | 515 | Dead | Male | No | Hispanic or Latino | Yes | Asian |
| TCGA-02-0266 | 539 | Dead | Male | No | Hispanic or Latino | Yes | White |
| TCGA-02-0269 | 327 | Dead | Male | No | Not Hispanic or Latino | Yes | White |
| TCGA-02-0285 | 422 | Dead | Female | No | Not Hispanic or Latino | Yes | White |
| TCGA-02-0290 | 485 | Dead | Male | Yes | Not Hispanic or Latino | Yes | White |
| TCGA-02-0317 | 372 | Dead | Male | Yes | Not Hispanic or Latino | Yes | White |
| TCGA-02-0321 | 301 | Dead | Male | No | Not Hispanic or Latino | Yes | White |
| TCGA-02-0324 | 235 | Dead | Female | No | Not Hispanic or Latino | Yes | White |
| TCGA-02-0325 | 323 | Dead | Male | Yes | Not Hispanic or Latino | Yes | White |
| TCGA-02-0326 | 223 | Dead | Female | No | Not Hispanic or Latino | Yes | White |
| TCGA-02-0332 | 782 | Dead | Female | Yes | Not Hispanic or Latino | Yes | White |
| TCGA-02-0333 | 133 | Dead | Female | No | Not Hispanic or Latino | Yes | White |
| TCGA-02-0337 | 764 | Dead | Male | Yes | Not Hispanic or Latino | Yes | Black or African American |
| TCGA-02-0338 | 322 | Dead | Male | No | Not Hispanic or Latino | Yes | White |
| TCGA-02-0339 | 377 | Dead | Male | No | Not Hispanic or Latino | Yes | White |
| TCGA-02-0422 | 441 | Dead | Male | No | Not Hispanic or Latino | Yes | White |
| TCGA-02-0430 | 321 | Dead | Female | No | Not Hispanic or Latino | Yes | White |
| TCGA-02-0432 | 1433 | Dead | Male | Yes | Not Hispanic or Latino | Yes | White |
| TCGA-02-0439 | 20 | Dead | Female | No | Not Hispanic or Latino | Yes | White |
| TCGA-02-0440 | 345 | Dead | Male | Yes | Not Hispanic or Latino | Yes | White |
| TCGA-02-0446 | 282 | Dead | Male | No | Not Hispanic or Latino | Yes | White |
| TCGA-02-0451 | 493 | Dead | Female | No | Not Hispanic or Latino | Yes | White |
| TCGA-02-0456 | 102 | Dead | Female | No | Not Hispanic or Latino | Yes | White |
| TCGA-02-2466 | 511 | Dead | Male | Yes | Not Hispanic or Latino | Yes | White |
| TCGA-02-2470 | 393 | Dead | Male | Yes | Not Hispanic or Latino | Yes | White |
| TCGA-02-2483 | 466 | Alive | Male | Yes | Not Hispanic or Latino | Yes | White |
| TCGA-02-2485 | 470 | Alive | Male | Yes | Not Hispanic or Latino | Yes | White |
| TCGA-02-2486 | 618 | Dead | Male | Yes | Not Hispanic or Latino | Yes | White |
| TCGA-06-0124 | 620 | Dead | Male | Yes | Not Hispanic or Latino | Yes | Black or African American |
| TCGA-06-0125 | 1448 | Dead | Female | Yes | Not Hispanic or Latino | Yes | White |
| TCGA-06-0127 | 121 | Dead | Male | Yes | Not Hispanic or Latino | Yes | White |
| TCGA-06-0128 | 691 | Dead | Male | Yes | Not Hispanic or Latino | Yes | White |
| TCGA-06-0129 | 1024 | Dead | Male | Yes | Not Hispanic or Latino | Yes | White |
| TCGA-06-0130 | 394 | Dead | Male | Yes | Not Hispanic or Latino | Yes | White |
| TCGA-06-0132 | 771 | Dead | Male | No | Not Hispanic or Latino | Yes | White |
| TCGA-06-0133 | 435 | Dead | Male | Yes | Not Hispanic or Latino | Yes | White |
| TCGA-06-0137 | 812 | Dead | Female | Yes | Not Hispanic or Latino | Yes | Asian |
| TCGA-06-0138 | 737 | Dead | Male | No | Not Hispanic or Latino | Yes | White |
| TCGA-06-0141 | 313 | Dead | Male | Yes | Not Hispanic or Latino | Yes | White |
| TCGA-06-0143 | 357 | Dead | Male | No | Not Hispanic or Latino | Yes | White |
| TCGA-06-0145 | 71 | Dead | Female | Yes | Not Hispanic or Latino | No | White |
| TCGA-06-0146 | 611 | Dead | Female | Yes | Not Hispanic or Latino | Yes | White |
| TCGA-06-0147 | 541 | Dead | Female | Yes | Not Hispanic or Latino | Yes | White |
| TCGA-06-0148 | 307 | Dead | Male | No | Not Hispanic or Latino | Yes | White |
| TCGA-06-0149 | 262 | Dead | Female | No | Not Hispanic or Latino | Yes | White |
| TCGA-06-0152 | 375 | Dead | Male | Yes | Not Hispanic or Latino | Yes | White |
| TCGA-06-0154 | 424 | Dead | Male | Yes | Not Hispanic or Latino | Yes | White |
| TCGA-06-0155 | 318 | Dead | Male | Yes | Not Hispanic or Latino | Yes | White |
| TCGA-06-0156 | 178 | Dead | Male | Yes | Not Hispanic or Latino | Yes | Black or African American |
| TCGA-06-0157 | 97 | Dead | Female | No | Not Hispanic or Latino | Yes | White |
| TCGA-06-0158 | 329 | Dead | Male | Yes | Not Hispanic or Latino | Yes | White |
| TCGA-06-0162 | 104 | Dead | Female | Yes | Not Hispanic or Latino | Yes | White |
| TCGA-06-0164 | 1731 | Dead | Male | No | Not Hispanic or Latino | Yes | White |
| TCGA-06-0166 | 178 | Dead | Male | Yes | Not Hispanic or Latino | No | White |
| TCGA-06-0168 | 598 | Dead | Female | Yes | Not Hispanic or Latino | Yes | White |
| TCGA-06-0169 | 100 | Dead | Male | Yes | Not Hispanic or Latino | Yes | White |
| TCGA-06-0171 | 399 | Dead | Male | Yes | Not Hispanic or Latino | Yes | White |
| TCGA-06-0173 | 136 | Dead | Female | No | Not Hispanic or Latino | No | White |
| TCGA-06-0174 | 98 | Dead | Male | Yes | Not Hispanic or Latino | Yes | White |
| TCGA-06-0175 | 123 | Dead | Male | Yes | Not Hispanic or Latino | No | Asian |
| TCGA-06-0176 | 2768 | Alive | Male | Yes | Not Hispanic or Latino | Yes | White |
| TCGA-06-0177 | 127 | Dead | Male | No | Not Hispanic or Latino | No | White |
| TCGA-06-0178 | 2681 | Dead | Male | Yes | Not Hispanic or Latino | Yes | Black or African American |
| TCGA-06-0179 | 616 | Dead | Male | No | Not Hispanic or Latino | Yes | White |
| TCGA-06-0182 | 111 | Dead | Male | No | Not Hispanic or Latino | No | White |
| TCGA-06-0184 | 2126 | Dead | Male | Yes | Not Hispanic or Latino | Yes | White |
| TCGA-06-0185 | 2246 | Alive | Male | Yes | Not Hispanic or Latino | Yes | White |
| TCGA-06-0187 | 828 | Dead | Male | No | Not Hispanic or Latino | Yes | White |
| TCGA-06-0188 | 1356 | Dead | Male | Yes | Not Hispanic or Latino | Yes | White |
| TCGA-06-0189 | 469 | Dead | Male | No | Not Hispanic or Latino | Yes | Asian |
| TCGA-06-0190 | 317 | Dead | Male | No | Not Hispanic or Latino | Yes | White |
| TCGA-06-0192 | 1185 | Dead | Male | Yes | Not Hispanic or Latino | Yes | White |
| TCGA-06-0194 | 142 | Dead | Female | No | Not Hispanic or Latino | Yes | White |
| TCGA-06-0195 | 225 | Dead | Male | Yes | Not Hispanic or Latino | Yes | White |
| TCGA-06-0197 | 169 | Dead | Female | Yes | Not Hispanic or Latino | No | White |
| TCGA-06-0201 | 12 | Dead | Female | No | Not Hispanic or Latino | No | White |
| TCGA-06-0206 | 233 | Dead | Male | Yes | Not Hispanic or Latino | Yes | White |
| TCGA-06-0208 | 256 | Dead | Female | Yes | Not Hispanic or Latino | Yes | White |
| TCGA-06-0210 | 225 | Dead | Female | No | Not Hispanic or Latino | Yes | White |
| TCGA-06-0211 | 360 | Dead | Male | Yes | Not Hispanic or Latino | Yes | White |
| TCGA-06-0213 | 16 | Dead | Female | No | Not Hispanic or Latino | No | White |
| TCGA-06-0214 | 457 | Dead | Male | Yes | Not Hispanic or Latino | No | White |
| TCGA-06-0216 | 735 | Dead | Female | Yes | Not Hispanic or Latino | Yes | White |
| TCGA-06-0219 | 22 | Dead | Male | No | Not Hispanic or Latino | No | White |
| TCGA-06-0221 | 603 | Dead | Male | Yes | Not Hispanic or Latino | Yes | White |
| TCGA-06-0237 | 415 | Dead | Female | No | Not Hispanic or Latino | Yes | White |
| TCGA-06-0238 | 405 | Dead | Male | No | Not Hispanic or Latino | Yes | White |
| TCGA-06-0241 | 1481 | Dead | Female | Yes | Not Hispanic or Latino | Yes | White |
| TCGA-06-0394 | 329 | Dead | Male | Yes | Not Hispanic or Latino | Yes | White |
| TCGA-06-0397 | 274 | Dead | Female | Yes | Not Hispanic or Latino | Yes | White |
| TCGA-06-0402 | 8 | Dead | Male | No | Not Hispanic or Latino | No | White |
| TCGA-06-0409 | 2201 | Dead | Male | Yes | Not Hispanic or Latino | Yes | White |
| TCGA-06-0410 | 143 | Dead | Female | No | Not Hispanic or Latino | No | White |
| TCGA-06-0412 | 291 | Dead | Female | Yes | Not Hispanic or Latino | Yes | White |
| TCGA-06-0413 | 96 | Dead | Female | No | Not Hispanic or Latino | No | White |
| TCGA-06-0414 | 1068 | Dead | Male | Yes | Not Hispanic or Latino | Yes | White |
| TCGA-06-0644 | 384 | Dead | Male | Yes | Not Hispanic or Latino | Yes | White |
| TCGA-06-0645 | 175 | Dead | Female | No | Not Hispanic or Latino | No | White |
| TCGA-06-0646 | 175 | Dead | Male | No | Not Hispanic or Latino | Yes | Black or African American |
| TCGA-06-0648 | 298 | Dead | Male | No | Not Hispanic or Latino | Yes | White |
| TCGA-06-0649 | 64 | Dead | Female | No | Not Hispanic or Latino | No | White |
| TCGA-06-0650 | 717 | Dead | Female | Yes | Not Hispanic or Latino | Yes | Black or African American |
| TCGA-06-0686 | 432 | Dead | Male | Yes | Not Hispanic or Latino | Yes | White |
| TCGA-06-0743 | 803 | Dead | Male | Yes | Not Hispanic or Latino | Yes | White |
| TCGA-06-0744 | 1426 | Dead | Male | Yes | Not Hispanic or Latino | Yes | White |
| TCGA-06-0745 | 239 | Dead | Male | No | Not Hispanic or Latino | Yes | White |
| TCGA-06-0747 | 82 | Dead | Male | No | Not Hispanic or Latino | Yes | Black or African American |
| TCGA-06-0749 | 82 | Dead | Male | No | Not Hispanic or Latino | Yes | White |
| TCGA-06-0750 | 28 | Dead | Male | No | Not Hispanic or Latino | No | White |
| TCGA-06-0875 | 1322 | Alive | Female | Yes | Not Hispanic or Latino | Yes | White |
| TCGA-06-0876 | 1405 | Alive | Female | Yes | Not Hispanic or Latino | Yes | Black or African American |
| TCGA-06-0877 | 204 | Alive | Male | No | Not Hispanic or Latino | Yes | White |
| TCGA-06-0878 | 218 | Alive | Male | Yes | Not Hispanic or Latino | Yes | White |
| TCGA-06-0879 | 1229 | Dead | Male | Yes | Not Hispanic or Latino | Yes | White |
| TCGA-06-0881 | 504 | Dead | Male | Yes | Not Hispanic or Latino | Yes | White |
| TCGA-06-0882 | 632 | Dead | Male | Yes | Not Hispanic or Latino | Yes | White |
| TCGA-06-0939 | 814 | Dead | Female | Yes | Not Hispanic or Latino | Yes | White |
| TCGA-06-1084 | 728 | Dead | Male | Yes | Not Hispanic or Latino | Yes | Black or African American |
| TCGA-06-1086 | 208 | Dead | Male | Yes | Not Hispanic or Latino | Yes | White |
| TCGA-06-1087 | 123 | Dead | Male | Yes | Not Hispanic or Latino | Yes | White |
| TCGA-06-1800 | 815 | Dead | Male | Yes | Not Hispanic or Latino | Yes | Black or African American |
| TCGA-06-1801 | 360 | Dead | Female | Yes | Not Hispanic or Latino | Yes | White |
| TCGA-06-1802 | 466 | Dead | Male | Yes | Not Hispanic or Latino | Yes | White |
| TCGA-06-1804 | 414 | Dead | Female | No | Not Hispanic or Latino | No | White |
| TCGA-06-1805 | 1031 | Alive | Female | Yes | Not Hispanic or Latino | Yes | White |
| TCGA-06-2557 | 33 | Dead | Male | No | Not Hispanic or Latino | No | White |
| TCGA-06-2558 | 380 | Dead | Female | No | Not Hispanic or Latino | Yes | White |
| TCGA-06-2559 | 150 | Dead | Male | Yes | Not Hispanic or Latino | Yes | Black or African American |
| TCGA-06-2561 | 537 | Dead | Female | Yes | Not Hispanic or Latino | Yes | Black or African American |
| TCGA-06-2562 | 382 | Dead | Male | Yes | Not Hispanic or Latino | Yes | White |
| TCGA-06-2563 | 932 | Alive | Female | Yes | Not Hispanic or Latino | Yes | White |
| TCGA-06-2564 | 181 | Alive | Male | Yes | Not Hispanic or Latino | Yes | White |
| TCGA-06-2565 | 506 | Dead | Male | Yes | Not Hispanic or Latino | Yes | White |
| TCGA-06-2566 | 182 | Dead | Female | Yes | Not Hispanic or Latino | Yes | White |
| TCGA-06-2567 | 133 | Dead | Male | Yes | Not Hispanic or Latino | Yes | White |
| TCGA-06-2570 | 958 | Alive | Female | Yes | Not Hispanic or Latino | Yes | Black or African American |
| TCGA-06-5408 | 357 | Dead | Female | Yes | Not Hispanic or Latino | Yes | White |
| TCGA-06-5410 | 108 | Dead | Female | No | Not Hispanic or Latino | No | White |
| TCGA-06-5411 | 254 | Dead | Male | Yes | Not Hispanic or Latino | Yes | White |
| TCGA-06-5412 | 138 | Dead | Female | Yes | Not Hispanic or Latino | Yes | White |
| TCGA-06-5413 | 268 | Alive | Male | Yes | Not Hispanic or Latino | Yes | White |
| TCGA-06-5414 | 273 | Alive | Male | Yes | Not Hispanic or Latino | Yes | White |
| TCGA-06-5415 | 260 | Alive | Male | Yes | Not Hispanic or Latino | Yes | Asian |
| TCGA-06-5416 | 204 | Alive | Female | Yes | Not Hispanic or Latino | Yes | White |
| TCGA-06-5417 | 155 | Alive | Female | Yes | Not Hispanic or Latino | Yes | White |
| TCGA-06-5418 | 83 | Dead | Female | No | Not Hispanic or Latino | No | Black or African American |
| TCGA-06-5856 | 114 | Dead | Male | No | Not Hispanic or Latino | No | White |
| TCGA-06-5858 | 187 | Alive | Female | Yes | Not Hispanic or Latino | Yes | White |
| TCGA-06-5859 | 139 | Alive | Male | Yes | Not Hispanic or Latino | Yes | White |
| TCGA-06-6389 | 237 | Alive | Female | Yes | Not Hispanic or Latino | Yes | White |
| TCGA-06-6390 | 164 | Dead | Male | Yes | Not Hispanic or Latino | Yes | White |
| TCGA-06-6391 | 45 | Dead | Female | No | Not Hispanic or Latino | No | White |
| TCGA-08-0244 | 690 | Dead | Male | Yes | Not Hispanic or Latino | Yes | White |
| TCGA-08-0245 | 1151 | Dead | Female | Yes | Not Hispanic or Latino | Yes | White |
| TCGA-08-0246 | 127 | Dead | Female | Yes | Not Hispanic or Latino | Yes | White |
| TCGA-08-0344 | 3524 | Dead | Male | Yes | Not Hispanic or Latino | Yes | White |
| TCGA-08-0348 | 370 | Dead | Male | Yes | Not Hispanic or Latino | Yes | White |
| TCGA-08-0349 | 298 | Dead | Male | Yes | Not Hispanic or Latino | Yes | White |
| TCGA-08-0350 | 889 | Dead | Male | Yes | Not Hispanic or Latino | Yes | White |
| TCGA-08-0351 | 1987 | Dead | Male | Yes | Not Hispanic or Latino | Yes | White |
| TCGA-08-0352 | 39 | Dead | Male | No | Not Hispanic or Latino | No | White |
| TCGA-08-0353 | 256 | Dead | Male | Yes | Not Hispanic or Latino | Yes | White |
| TCGA-08-0354 | 546 | Dead | Female | Yes | Not Hispanic or Latino | Yes | White |
| TCGA-08-0355 | 747 | Dead | Female | Yes | Not Hispanic or Latino | Yes | White |
| TCGA-08-0356 | 946 | Dead | Female | Yes | Not Hispanic or Latino | Yes | White |
| TCGA-08-0357 | 1143 | Dead | Male | Yes | Not Hispanic or Latino | Yes | White |
| TCGA-08-0358 | 678 | Dead | Male | Yes | Not Hispanic or Latino | Yes | White |
| TCGA-08-0359 | 103 | Dead | Female | No | Not Hispanic or Latino | Yes | White |
| TCGA-08-0360 | 468 | Dead | Male | Yes | Not Hispanic or Latino | Yes | White |
| TCGA-08-0386 | 548 | Dead | Male | Yes | Not Hispanic or Latino | Yes | Black or African American |
| TCGA-08-0389 | 467 | Dead | Male | Yes | Not Hispanic or Latino | Yes | Black or African American |
| TCGA-08-0390 | 425 | Dead | Male | Yes | Not Hispanic or Latino | Yes | White |
| TCGA-08-0392 | 22 | Dead | Male | No | Not Hispanic or Latino | No | Black or African American |
| TCGA-08-0510 | 130 | Dead | Male | Yes | Not Hispanic or Latino | Yes | White |
| TCGA-08-0516 | 596 | Dead | Male | No | Not Hispanic or Latino | Yes | White |
| TCGA-08-0518 | 588 | Dead | Female | No | Not Hispanic or Latino | Yes | White |
| TCGA-08-0520 | 327 | Dead | Male | Yes | Not Hispanic or Latino | Yes | White |
| TCGA-08-0521 | 146 | Dead | Male | Yes | Hispanic or Latino | Yes | White |
| TCGA-08-0524 | 221 | Dead | Female | Yes | Not Hispanic or Latino | Yes | White |
| TCGA-08-0525 | 486 | Dead | Male | Yes | Not Hispanic or Latino | Yes | White |
| TCGA-08-0529 | 560 | Dead | Female | Yes | Hispanic or Latino | Yes | White |
| TCGA-08-0531 | 230 | Dead | Male | Yes | Not Hispanic or Latino | Yes | White |
| TCGA-12-0616 | 448 | Dead | Female | Yes | Not Hispanic or Latino | Yes | White |
| TCGA-12-0618 | 395 | Dead | Male | Yes | Not Hispanic or Latino | Yes | White |
| TCGA-12-0619 | 1062 | Dead | Male | Yes | Not Hispanic or Latino | Yes | White |
| TCGA-12-0670 | 790 | Dead | Male | Yes | Not Hispanic or Latino | Yes | White |
| TCGA-12-0692 | 111 | Dead | Female | Yes | Not Hispanic or Latino | No | White |
| TCGA-12-0703 | 620 | Dead | Male | Yes | Not Hispanic or Latino | Yes | White |
| TCGA-12-0772 | 1638 | Dead | Male | Yes | Not Hispanic or Latino | Yes | White |
| TCGA-12-0773 | 1315 | Dead | Male | Yes | Not Hispanic or Latino | Yes | White |
| TCGA-12-0775 | 232 | Dead | Female | Yes | Not Hispanic or Latino | Yes | White |
| TCGA-12-0776 | 296 | Dead | Male | Yes | Not Hispanic or Latino | Yes | White |
| TCGA-12-0778 | 454 | Dead | Male | Yes | Not Hispanic or Latino | Yes | White |
| TCGA-12-0780 | 452 | Dead | Female | Yes | Not Hispanic or Latino | Yes | White |
| TCGA-12-0818 | 2791 | Dead | Female | Yes | Not Hispanic or Latino | Yes | White |
| TCGA-12-0819 | 754 | Dead | Female | Yes | Not Hispanic or Latino | Yes | White |
| TCGA-12-0820 | 562 | Dead | Male | Yes | Not Hispanic or Latino | Yes | Black or African American |
| TCGA-12-0821 | 323 | Dead | Male | Yes | Not Hispanic or Latino | Yes | White |
| TCGA-12-0822 | 715 | Dead | Male | Yes | Not Hispanic or Latino | Yes | White |
| TCGA-12-0828 | 272 | Dead | Male | Yes | Not Hispanic or Latino | Yes | White |
| TCGA-12-0829 | 626 | Dead | Male | Yes | Not Hispanic or Latino | Yes | White |
| TCGA-12-1088 | 3881 | Dead | Female | Yes | Not Hispanic or Latino | Yes | White |
| TCGA-12-1089 | 177 | Dead | Male | Yes | Not Hispanic or Latino | Yes | White |
| TCGA-12-1090 | 231 | Dead | Male | Yes | Not Hispanic or Latino | Yes | White |
| TCGA-12-1091 | 1010 | Dead | Female | Yes | Not Hispanic or Latino | Yes | White |
| TCGA-12-1092 | 661 | Dead | Male | Yes | Not Hispanic or Latino | Yes | White |
| TCGA-12-1093 | 486 | Dead | Female | Yes | Not Hispanic or Latino | Yes | White |
| TCGA-12-1094 | 372 | Dead | Male | Yes | Not Hispanic or Latino | Yes | Black or African American |
| TCGA-12-1095 | 482 | Dead | Female | Yes | Not Hispanic or Latino | Yes | White |
| TCGA-12-1096 | 277 | Dead | Male | Yes | Not Hispanic or Latino | No | White |
| TCGA-12-1097 | 442 | Dead | Male | Yes | Not Hispanic or Latino | Yes | White |
| TCGA-12-1098 | 121 | Dead | Female | Yes | Not Hispanic or Latino | Yes | White |
| TCGA-12-1099 | 126 | Dead | Female | Yes | Not Hispanic or Latino | Yes | White |
| TCGA-12-1597 | 675 | Dead | Female | Yes | Not Hispanic or Latino | Yes | White |
| TCGA-12-1598 | 476 | Dead | Female | Yes | Not Hispanic or Latino | Yes | White |
| TCGA-12-1599 | 781 | Dead | Female | Yes | Not Hispanic or Latino | Yes | White |
| TCGA-12-1600 | 448 | Dead | Male | Yes | Not Hispanic or Latino | Yes | White |
| TCGA-12-1602 | 206 | Dead | Male | Yes | Not Hispanic or Latino | Yes | White |
| TCGA-12-3644 | 1818 | Dead | Female | Yes | Not Hispanic or Latino | Yes | White |
| TCGA-12-3646 | 1339 | Dead | Female | Yes | Not Hispanic or Latino | Yes | Black or African American |
| TCGA-12-3648 | 819 | Dead | Female | Yes | Not Hispanic or Latino | Yes | White |
| TCGA-12-3649 | 463 | Dead | Male | Yes | Not Hispanic or Latino | No | White |
| TCGA-12-3650 | 333 | Dead | Male | Yes | Not Hispanic or Latino | Yes | White |
| TCGA-12-3651 | 386 | Dead | Male | Yes | Not Hispanic or Latino | Yes | White |
| TCGA-12-3652 | 1062 | Dead | Male | Yes | Not Hispanic or Latino | Yes | Black or African American |
| TCGA-12-3653 | 442 | Dead | Female | Yes | Not Hispanic or Latino | Yes | White |
| TCGA-12-5295 | 454 | Dead | Female | Yes | Not Hispanic or Latino | Yes | White |
| TCGA-12-5299 | 98 | Dead | Female | Yes | Not Hispanic or Latino | Yes | White |
| TCGA-12-5301 | 62 | Dead | Male | Yes | Not Hispanic or Latino | Yes | White |
| TCGA-14-0787 | 68 | Dead | Male | Yes | Not Hispanic or Latino | No | White |
| TCGA-14-1825 | 232 | Dead | Male | Yes | Not Hispanic or Latino | Yes | White |
| TCGA-14-3477 | 115 | Alive | Female | Yes | Not Hispanic or Latino | Yes | Asian |
| TCGA-15-0742 | 419 | Dead | Male | Yes | Not Hispanic or Latino | Yes | White |
| TCGA-16-0846 | 119 | Dead | Male | No | Not Hispanic or Latino | No | White |
| TCGA-16-0848 | 535 | Dead | Male | No | Not Hispanic or Latino | Yes | White |
| TCGA-16-0849 | 793 | Alive | Male | No | Not Hispanic or Latino | Yes | White |
| TCGA-16-0850 | 498 | Dead | Female | No | Not Hispanic or Latino | Yes | White |
| TCGA-16-1045 | 883 | Dead | Female | No | Not Hispanic or Latino | Yes | White |
| TCGA-16-1047 | 139 | Dead | Female | No | Not Hispanic or Latino | No | White |
| TCGA-16-1055 | 313 | Dead | Male | No | Not Hispanic or Latino | Yes | White |
| TCGA-16-1056 | 426 | Dead | Male | No | Not Hispanic or Latino | Yes | White |
| TCGA-16-1060 | 278 | Dead | Female | No | Not Hispanic or Latino | Yes | White |
| TCGA-16-1062 | 646 | Dead | Female | No | Not Hispanic or Latino | Yes | White |
| TCGA-16-1063 | 425 | Dead | Male | No | Not Hispanic or Latino | Yes | Black or African American |
| TCGA-16-1460 | 195 | Alive | Female | No | Not Hispanic or Latino | Yes | White |
| TCGA-19-0955 | 358 | Alive | Male | Yes | Not Hispanic or Latino | Yes | White |
| TCGA-19-0957 | 666 | Dead | Female | Yes | Not Hispanic or Latino | Yes | White |
| TCGA-19-0960 | 262 | Alive | Female | Yes | Not Hispanic or Latino | Yes | White |
| TCGA-19-0962 | 20 | Dead | Female | No | Not Hispanic or Latino | No | White |
| TCGA-19-0963 | 434 | Dead | Male | Yes | Not Hispanic or Latino | Yes | White |
| TCGA-19-0964 | 103 | Alive | Male | Yes | Not Hispanic or Latino | Yes | White |
| TCGA-19-1385 | 327 | Dead | Male | Yes | Not Hispanic or Latino | Yes | White |
| TCGA-19-1387 | 181 | Alive | Male | Yes | Not Hispanic or Latino | Yes | White |
| TCGA-19-1388 | 394 | Dead | Male | Yes | Not Hispanic or Latino | Yes | White |
| TCGA-19-1389 | 141 | Dead | Male | Yes | Not Hispanic or Latino | Yes | White |
| TCGA-19-1390 | 772 | Dead | Female | No | Not Hispanic or Latino | No | White |
| TCGA-19-1392 | 111 | Dead | Female | No | Not Hispanic or Latino | No | White |
| TCGA-19-1786 | 218 | Alive | Female | No | Not Hispanic or Latino | Yes | Black or African American |
| TCGA-19-1787 | 385 | Dead | Male | Yes | Not Hispanic or Latino | Yes | White |
| TCGA-19-1788 | 112 | Dead | Male | Yes | Not Hispanic or Latino | No | White |
| TCGA-19-1789 | 99 | Dead | Female | Yes | Not Hispanic or Latino | Yes | White |
| TCGA-19-1790 | 154 | Dead | Male | Yes | Not Hispanic or Latino | Yes | Black or African American |
| TCGA-19-1791 | 4 | Alive | Female | No | Not Hispanic or Latino | No | Black or African American |
| TCGA-19-2619 | 294 | Alive | Female | Yes | Not Hispanic or Latino | Yes | White |
| TCGA-19-2620 | 148 | Dead | Male | Yes | Not Hispanic or Latino | Yes | White |
| TCGA-19-2621 | 33 | Dead | Male | No | Not Hispanic or Latino | No | White |
| TCGA-19-2623 | 229 | Alive | Male | Yes | Not Hispanic or Latino | Yes | White |
| TCGA-19-2624 | 5 | Dead | Male | No | Not Hispanic or Latino | No | White |
| TCGA-19-2625 | 124 | Dead | Female | No | Not Hispanic or Latino | No | White |
| TCGA-19-2629 | 737 | Dead | Male | Yes | Not Hispanic or Latino | Yes | White |
| TCGA-19-2631 | 213 | Dead | Female | No | Not Hispanic or Latino | Yes | White |
| TCGA-19-4068 | 137 | Alive | Female | Yes | Not Hispanic or Latino | Yes | White |
| TCGA-19-5947 | 202 | Dead | Female | No | Not Hispanic or Latino | Yes | White |
| TCGA-19-5950 | 523 | Dead | Female | Yes | Not Hispanic or Latino | Yes | White |
| TCGA-19-5952 | 575 | Dead | Male | No | Not Hispanic or Latino | Yes | White |
| TCGA-19-5954 | 368 | Dead | Female | Yes | Not Hispanic or Latino | Yes | White |
| TCGA-19-5955 | 54 | Dead | Male | No | Not Hispanic or Latino | No | White |
| TCGA-19-5956 | 684 | Dead | Female | Yes | Not Hispanic or Latino | Yes | White |
| TCGA-19-5958 | 428 | Dead | Male | Yes | Not Hispanic or Latino | Yes | White |
| TCGA-19-5959 | 511 | Dead | Female | Yes | Not Hispanic or Latino | Yes | White |
| TCGA-19-5960 | 455 | Dead | Male | Yes | Not Hispanic or Latino | Yes | Asian |
| TCGA-26-1438 | 305 | Alive | Male | Yes | Not Hispanic or Latino | Yes | White |
| TCGA-26-1439 | 422 | Dead | Male | Yes | Not Hispanic or Latino | Yes | White |
| TCGA-26-1440 | 296 | Alive | Male | Yes | Not Hispanic or Latino | Yes | White |
| TCGA-26-1442 | 953 | Alive | Male | Yes | Not Hispanic or Latino | Yes | White |
| TCGA-26-1443 | 217 | Alive | Female | Yes | Not Hispanic or Latino | Yes | White |
| TCGA-26-1799 | 285 | Dead | Male | Yes | Not Hispanic or Latino | Yes | White |
| TCGA-26-5132 | 286 | Alive | Male | Yes | Not Hispanic or Latino | Yes | White |
| TCGA-26-5133 | 452 | Alive | Male | Yes | Not Hispanic or Latino | Yes | White |
| TCGA-26-5134 | 167 | Alive | Male | No | Not Hispanic or Latino | Yes | White |
| TCGA-26-5139 | 48 | Alive | Female | No | Not Hispanic or Latino | No | White |
| TCGA-27-1830 | 154 | Dead | Male | Yes | Not Hispanic or Latino | Yes | Asian |
| TCGA-27-1831 | 505 | Dead | Male | Yes | Not Hispanic or Latino | Yes | White |
| TCGA-27-1832 | 300 | Dead | Female | Yes | Not Hispanic or Latino | Yes | White |
| TCGA-27-1833 | 737 | Dead | Female | Yes | Not Hispanic or Latino | Yes | White |
| TCGA-27-1834 | 1233 | Dead | Male | Yes | Not Hispanic or Latino | Yes | White |
| TCGA-27-1835 | 648 | Dead | Female | Yes | Not Hispanic or Latino | Yes | White |
| TCGA-27-1836 | 914 | Dead | Female | Yes | Not Hispanic or Latino | Yes | Black or African American |
| TCGA-27-1837 | 427 | Dead | Male | Yes | Not Hispanic or Latino | Yes | White |
| TCGA-27-1838 | 350 | Dead | Female | Yes | Not Hispanic or Latino | Yes | White |
| TCGA-27-2518 | 753 | Dead | Male | Yes | Not Hispanic or Latino | Yes | White |
| TCGA-27-2519 | 550 | Dead | Male | Yes | Not Hispanic or Latino | Yes | White |
| TCGA-27-2521 | 510 | Dead | Male | Yes | Not Hispanic or Latino | Yes | White |
| TCGA-27-2523 | 489 | Dead | Male | Yes | Not Hispanic or Latino | Yes | White |
| TCGA-27-2524 | 231 | Dead | Male | Yes | Not Hispanic or Latino | Yes | White |
| TCGA-27-2526 | 87 | Dead | Female | Yes | Not Hispanic or Latino | No | White |
| TCGA-27-2527 | 438 | Dead | Male | Yes | Not Hispanic or Latino | Yes | White |
| TCGA-27-2528 | 480 | Dead | Male | Yes | Not Hispanic or Latino | Yes | Black or African American |
| TCGA-28-1745 | 345 | Alive | Male | Yes | Not Hispanic or Latino | Yes | White |
| TCGA-28-1746 | 6 | Alive | Female | No | Not Hispanic or Latino | Yes | White |
| TCGA-28-1747 | 77 | Dead | Male | Yes | Not Hispanic or Latino | Yes | White |
| TCGA-28-1749 | 280 | Alive | Male | Yes | Not Hispanic or Latino | Yes | White |
| TCGA-28-1750 | 254 | Alive | Female | Yes | Not Hispanic or Latino | Yes | White |
| TCGA-28-1751 | 232 | Alive | Female | Yes | Not Hispanic or Latino | Yes | White |
| TCGA-28-1752 | 258 | Alive | Female | Yes | Not Hispanic or Latino | Yes | White |
| TCGA-28-1753 | 37 | Alive | Male | No | Hispanic or Latino | Yes | White |
| TCGA-28-1755 | 47 | Dead | Female | Yes | Not Hispanic or Latino | Yes | White |
| TCGA-28-1756 | 86 | Alive | Male | Yes | Not Hispanic or Latino | Yes | White |
| TCGA-28-1760 | 143 | Alive | Male | Yes | Not Hispanic or Latino | Yes | Black or African American |
| TCGA-28-2502 | 20 | Alive | Male | No | Not Hispanic or Latino | Yes | White |
| TCGA-28-2506 | 205 | Alive | Female | Yes | Not Hispanic or Latino | Yes | White |
| TCGA-28-2509 | 145 | Alive | Female | Yes | Not Hispanic or Latino | Yes | White |
| TCGA-28-2513 | 222 | Alive | Female | Yes | Not Hispanic or Latino | Yes | White |
| TCGA-28-2514 | 160 | Alive | Male | Yes | Not Hispanic or Latino | Yes | White |
| TCGA-32-1973 | 641 | Dead | Male | Yes | Hispanic or Latino | Yes | White |
| TCGA-32-1976 | 15 | Dead | Male | No | Not Hispanic or Latino | No | White |
| TCGA-32-1980 | 36 | Dead | Male | No | Not Hispanic or Latino | No | White |
| TCGA-32-1986 | 386 | Dead | Male | Yes | Not Hispanic or Latino | Yes | White |
| TCGA-32-1987 | 452 | Dead | Female | Yes | Not Hispanic or Latino | Yes | White |
| TCGA-32-1991 | 515 | Dead | Male | Yes | Not Hispanic or Latino | Yes | White |
| TCGA-32-2491 | 372 | Dead | Male | Yes | Not Hispanic or Latino | Yes | White |
| TCGA-32-2494 | 632 | Dead | Female | Yes | Not Hispanic or Latino | Yes | White |
| TCGA-32-2495 | 457 | Dead | Female | Yes | Not Hispanic or Latino | Yes | White |
| TCGA-32-2615 | 485 | Dead | Male | Yes | Not Hispanic or Latino | Yes | White |
| TCGA-32-2616 | 224 | Dead | Female | Yes | Hispanic or Latino | Yes | White |
| TCGA-32-2634 | 693 | Alive | Male | Yes | Not Hispanic or Latino | Yes | White |
| TCGA-32-2638 | 766 | Dead | Male | Yes | Not Hispanic or Latino | Yes | White |
| TCGA-32-4208 | 643 | Alive | Male | Yes | Not Hispanic or Latino | Yes | White |
| TCGA-32-4209 | 618 | Dead | Male | Yes | Hispanic or Latino | Yes | White |
| TCGA-32-4211 | 383 | Dead | Male | Yes | Not Hispanic or Latino | Yes | White |
| TCGA-32-4213 | 604 | Alive | Female | Yes | Not Hispanic or Latino | Yes | White |
| TCGA-32-4719 | 330 | Dead | Male | Yes | Not Hispanic or Latino | Yes | White |
| TCGA-32-5222 | 585 | Dead | Male | Yes | Not Hispanic or Latino | Yes | White |
| TCGA-41-2571 | 26 | Dead | Male | Yes | Not Hispanic or Latino | No | White |
| TCGA-41-2572 | 406 | Dead | Male | Yes | Not Hispanic or Latino | Yes | White |
| TCGA-41-2573 | 272 | Alive | Male | Yes | Not Hispanic or Latino | Yes | White |
| TCGA-41-2575 | 290 | Dead | Male | Yes | Not Hispanic or Latino | Yes | White |
| TCGA-41-3392 | 30 | Dead | Male | No | Not Hispanic or Latino | No | White |
| TCGA-41-3393 | 135 | Dead | Female | Yes | Not Hispanic or Latino | Yes | White |
| TCGA-41-3915 | 184 | Alive | Male | Yes | Not Hispanic or Latino | Yes | Asian |
| TCGA-41-4097 | 6 | Dead | Female | No | Not Hispanic or Latino | No | White |
| TCGA-41-5651 | 460 | Dead | Female | Yes | Not Hispanic or Latino | Yes | White |
| TCGA-76-4925 | 146 | Dead | Male | Yes | Not Hispanic or Latino | Yes | White |
| TCGA-76-6192 | 100 | Dead | Male | Yes | Not Hispanic or Latino | Yes | White |
| TCGA-76-6282 | 519 | Dead | Male | Yes | Not Hispanic or Latino | Yes | White |
| TCGA-87-5896 | 800 | Alive | Female | Yes | Not Hispanic or Latino | Yes | White |
