# Supplementary material for: Investigation of Genetic Determinants of Glioma Immune Phenotype by Integrative Immunogenomic Scale Analysis
Source: Front Immunol. 2021 Jun 16;12:557994. doi: 10.3389/fimmu.2021.557994 (PMC8242587; doi:10.3389/fimmu.2021.557994)
Supplement: Supplementary file 3 [file Table_2.docx]

**Supplementary Online File 2. Clinical data for TCGA GBM RNA-seq samples.**

| id | OS (day) | Survival status | Age | Gender | Radiation | Pharmaceutical therapy | Adjuvant TMZ chemotherapy | Histology subtype |
| --- | --- | --- | --- | --- | --- | --- | --- | --- |
| TCGA-19-2625 | 124 | Dead | 70-79 | Female | No | No | No | Classical |
| TCGA-06-5856 | 114 | Dead | 50-59 | Male | No | No | No | Classical |
| TCGA-06-0157 | 97 | Dead | 60-69 | Female | Yes | No | No | Classical |
| TCGA-06-0158 | 329 | Dead | 70-79 | Male | Yes | No | No | Classical |
| TCGA-14-1402 | 975 | Dead | 50-59 | Female | Yes | Yes | No | Classical |
| TCGA-06-0743 | 679 | Alive | 60-69 | Male | Yes | Yes | No | Classical |
| TCGA-41-2572 | 406 | Dead | 60-69 | Male | Yes | Yes | No | Classical |
| TCGA-32-2638 | 224 | Alive | 60-69 | Male | No | Yes | Yes | Classical |
| TCGA-19-2619 | 294 | Alive | 50-59 | Female | Yes | Yes | Yes | Classical |
| TCGA-06-2563 | 259 | Alive | 70-79 | Female | Yes | Yes | Yes | Classical |
| TCGA-12-3653 | 442 | Dead | < 50 | Female | Yes | Yes | Yes | Classical |
| TCGA-06-5408 | 357 | Dead | 50-59 | Female | Yes | Yes | Yes | Classical |
| TCGA-27-1835 | 648 | Dead | 50-59 | Female | Yes | Yes | Yes | Classical |
| TCGA-06-0125 | 1448 | Dead | 60-69 | Female | Yes | Yes | Yes | Classical |
| TCGA-14-0790 | 419 | Dead | 60-69 | Female | Yes | Yes | Yes | Classical |
| TCGA-76-4928 | 94 | Dead | > 80 | Female | Yes | Yes | Yes | Classical |
| TCGA-28-2514 | 160 | Alive | < 50 | Male | Yes | Yes | Yes | Classical |
| TCGA-02-2485 | 470 | Alive | 50-59 | Male | Yes | Yes | Yes | Classical |
| TCGA-06-2564 | 181 | Alive | 50-59 | Male | Yes | Yes | Yes | Classical |
| TCGA-06-2565 | 207 | Alive | 50-59 | Male | Yes | Yes | Yes | Classical |
| TCGA-06-0744 | 595 | Alive | 60-69 | Male | Yes | Yes | Yes | Classical |
| TCGA-06-5414 | 273 | Alive | 60-69 | Male | Yes | Yes | Yes | Classical |
| TCGA-28-5220 | 319 | Alive | 60-69 | Male | Yes | Yes | Yes | Classical |
| TCGA-26-5132 | 286 | Alive | 70-79 | Male | Yes | Yes | Yes | Classical |
| TCGA-06-0211 | 360 | Dead | < 50 | Male | Yes | Yes | Yes | Classical |
| TCGA-27-1837 | 427 | Dead | < 50 | Male | Yes | Yes | Yes | Classical |
| TCGA-32-1970 | 468 | Dead | 50-59 | Male | Yes | Yes | Yes | Classical |
| TCGA-06-0187 | 828 | Dead | 60-69 | Male | Yes | Yes | Yes | Classical |
| TCGA-12-3652 | 1062 | Dead | 60-69 | Male | Yes | Yes | Yes | Classical |
| TCGA-15-0742 | 419 | Dead | 60-69 | Male | Yes | Yes | Yes | Classical |
| TCGA-27-2523 | 489 | Dead | 60-69 | Male | Yes | Yes | Yes | Classical |
| TCGA-27-2528 | 480 | Dead | 60-69 | Male | Yes | Yes | Yes | Classical |
| TCGA-76-4926 | 138 | Dead | 60-69 | Male | Yes | Yes | Yes | Classical |
| TCGA-06-0645 | 175 | Dead | 50-59 | Female | No | No | No | Mesenchymal |
| TCGA-06-5410 | 108 | Dead | 70-79 | Female | No | No | No | Mesenchymal |
| TCGA-06-0210 | 225 | Dead | 70-79 | Female | Yes | No | No | Mesenchymal |
| TCGA-16-1045 | 883 | Dead | < 50 | Female | Yes | Yes | No | Mesenchymal |
| TCGA-14-1034 | 485 | Dead | 60-69 | Female | Yes | Yes | No | Mesenchymal |
| TCGA-14-0871 | 880 | Dead | 70-79 | Female | Yes | Yes | No | Mesenchymal |
| TCGA-26-5136 | 577 | Dead | 70-79 | Female | Yes | Yes | No | Mesenchymal |
| TCGA-14-0736 | 460 | Dead | < 50 | Male | Yes | Yes | No | Mesenchymal |
| TCGA-06-0130 | 394 | Dead | 50-59 | Male | Yes | Yes | No | Mesenchymal |
| TCGA-06-0152 | 375 | Dead | 60-69 | Male | Yes | Yes | No | Mesenchymal |
| TCGA-32-2632 | 269 | Dead | > 80 | Male | Yes | Yes | No | Mesenchymal |
| TCGA-06-5858 | 187 | Alive | < 50 | Female | Yes | Yes | Yes | Mesenchymal |
| TCGA-32-4213 | 357 | Alive | < 50 | Female | Yes | Yes | Yes | Mesenchymal |
| TCGA-06-2561 | 282 | Alive | 50-59 | Female | Yes | Yes | Yes | Mesenchymal |
| TCGA-28-2513 | 222 | Alive | 60-69 | Female | Yes | Yes | Yes | Mesenchymal |
| TCGA-28-2509 | 145 | Alive | 70-79 | Female | Yes | Yes | Yes | Mesenchymal |
| TCGA-32-2616 | 224 | Dead | < 50 | Female | Yes | Yes | Yes | Mesenchymal |
| TCGA-06-0168 | 598 | Dead | 50-59 | Female | Yes | Yes | Yes | Mesenchymal |
| TCGA-14-1823 | 543 | Dead | 50-59 | Female | Yes | Yes | Yes | Mesenchymal |
| TCGA-27-1832 | 300 | Dead | 50-59 | Female | Yes | Yes | Yes | Mesenchymal |
| TCGA-28-5215 | 335 | Dead | 60-69 | Female | Yes | Yes | Yes | Mesenchymal |
| TCGA-06-5412 | 138 | Dead | 70-79 | Female | Yes | Yes | Yes | Mesenchymal |
| TCGA-27-2519 | 316 | Alive | < 50 | Male | Yes | Yes | Yes | Mesenchymal |
| TCGA-28-5208 | 474 | Alive | 50-59 | Male | Yes | Yes | Yes | Mesenchymal |
| TCGA-28-5216 | 415 | Alive | 50-59 | Male | Yes | Yes | Yes | Mesenchymal |
| TCGA-02-2486 | 493 | Alive | 60-69 | Male | Yes | Yes | Yes | Mesenchymal |
| TCGA-06-0184 | 1228 | Alive | 60-69 | Male | Yes | Yes | Yes | Mesenchymal |
| TCGA-06-0644 | 375 | Alive | 70-79 | Male | Yes | Yes | Yes | Mesenchymal |
| TCGA-06-0878 | 218 | Alive | 70-79 | Male | Yes | Yes | Yes | Mesenchymal |
| TCGA-28-5213 | 298 | Alive | 70-79 | Male | Yes | Yes | Yes | Mesenchymal |
| TCGA-06-0139 | 362 | Dead | < 50 | Male | Yes | Yes | Yes | Mesenchymal |
| TCGA-19-1787 | 385 | Dead | < 50 | Male | Yes | Yes | Yes | Mesenchymal |
| TCGA-41-3915 | 360 | Dead | < 50 | Male | Yes | Yes | Yes | Mesenchymal |
| TCGA-14-0789 | 342 | Dead | 50-59 | Male | Yes | Yes | Yes | Mesenchymal |
| TCGA-19-1389 | 141 | Dead | 50-59 | Male | Yes | Yes | Yes | Mesenchymal |
| TCGA-27-1834 | 1233 | Dead | 50-59 | Male | Yes | Yes | Yes | Mesenchymal |
| TCGA-27-2524 | 231 | Dead | 50-59 | Male | Yes | Yes | Yes | Mesenchymal |
| TCGA-06-0141 | 313 | Dead | 60-69 | Male | Yes | Yes | Yes | Mesenchymal |
| TCGA-06-0190 | 317 | Dead | 60-69 | Male | Yes | Yes | Yes | Mesenchymal |
| TCGA-12-0619 | 1062 | Dead | 60-69 | Male | Yes | Yes | Yes | Mesenchymal |
| TCGA-28-5218 | 157 | Dead | 60-69 | Male | Yes | Yes | Yes | Mesenchymal |
| TCGA-32-2615 | 485 | Dead | 60-69 | Male | Yes | Yes | Yes | Mesenchymal |
| TCGA-28-5207 | 343 | Dead | 70-79 | Male | Yes | Yes | Yes | Mesenchymal |
| TCGA-32-1982 | 142 | Dead | 70-79 | Female | No | Yes | No | Neural |
| TCGA-14-2554 | 532 | Dead | 50-59 | Female | Yes | Yes | No | Neural |
| TCGA-06-0132 | 771 | Dead | < 50 | Male | Yes | Yes | No | Neural |
| TCGA-06-0221 | 603 | Dead | < 50 | Male | Yes | Yes | No | Neural |
| TCGA-76-4927 | 535 | Dead | 50-59 | Male | Yes | Yes | No | Neural |
| TCGA-12-0821 | 323 | Dead | 60-69 | Male | Yes | Yes | No | Neural |
| TCGA-14-0817 | 164 | Dead | 60-69 | Female | No | Yes | Yes | Neural |
| TCGA-12-5295 | 454 | Dead | 60-69 | Female | Yes | Yes | Yes | Neural |
| TCGA-06-0178 | 1642 | Alive | < 50 | Male | Yes | Yes | Yes | Neural |
| TCGA-06-0882 | 165 | Alive | < 50 | Male | Yes | Yes | Yes | Neural |
| TCGA-14-1829 | 218 | Alive | 50-59 | Male | Yes | Yes | Yes | Neural |
| TCGA-06-5413 | 268 | Alive | 60-69 | Male | Yes | Yes | Yes | Neural |
| TCGA-06-5859 | 139 | Alive | 60-69 | Male | Yes | Yes | Yes | Neural |
| TCGA-06-0138 | 737 | Dead | < 50 | Male | Yes | Yes | Yes | Neural |
| TCGA-06-5411 | 254 | Dead | 50-59 | Male | Yes | Yes | Yes | Neural |
| TCGA-06-0171 | 399 | Dead | 60-69 | Male | Yes | Yes | Yes | Neural |
| TCGA-06-2567 | 133 | Dead | 60-69 | Male | Yes | Yes | Yes | Neural |
| TCGA-27-1831 | 505 | Dead | 60-69 | Male | Yes | Yes | Yes | Neural |
| TCGA-08-0386 | 548 | Dead | 70-79 | Male | Yes | Yes | Yes | Neural |
| TCGA-19-2620 | 148 | Dead | 70-79 | Male | Yes | Yes | Yes | Neural |
| TCGA-28-5204 | 454 | Dead | 70-79 | Male | Yes | Yes | Yes | Neural |
| TCGA-19-1390 | 772 | Dead | 60-69 | Female | No | No | No | Proneural |
| TCGA-06-2558 | 380 | Dead | 70-79 | Female | Yes | No | No | Proneural |
| TCGA-02-0047 | 448 | Dead | 70-79 | Male | Yes | No | No | Proneural |
| TCGA-16-0846 | 119 | Dead | > 80 | Male | No | Yes | No | Proneural |
| TCGA-26-5135 | 270 | Dead | 70-79 | Female | Yes | Yes | No | Proneural |
| TCGA-26-5134 | 167 | Alive | 70-79 | Male | Yes | Yes | No | Proneural |
| TCGA-06-0129 | 1024 | Dead | < 50 | Male | Yes | Yes | No | Proneural |
| TCGA-06-0156 | 178 | Dead | 50-59 | Male | Yes | Yes | No | Proneural |
| TCGA-06-0174 | 98 | Dead | 50-59 | Male | Yes | Yes | No | Proneural |
| TCGA-06-0745 | 239 | Dead | 50-59 | Male | Yes | Yes | No | Proneural |
| TCGA-14-1825 | 232 | Dead | 70-79 | Male | Yes | Yes | No | Proneural |
| TCGA-32-2634 | 270 | Alive | > 80 | Male | No | Yes | Yes | Proneural |
| TCGA-02-2483 | 466 | Alive | < 50 | Female | Yes | Yes | Yes | Proneural |
| TCGA-06-2570 | 285 | Alive | < 50 | Female | Yes | Yes | Yes | Proneural |
| TCGA-06-5416 | 204 | Alive | < 50 | Female | Yes | Yes | Yes | Proneural |
| TCGA-06-5417 | 155 | Alive | < 50 | Female | Yes | Yes | Yes | Proneural |
| TCGA-41-5651 | 351 | Alive | 50-59 | Female | Yes | Yes | Yes | Proneural |
| TCGA-12-0616 | 448 | Dead | < 50 | Female | Yes | Yes | Yes | Proneural |
| TCGA-19-0957 | 666 | Dead | < 50 | Female | Yes | Yes | Yes | Proneural |
| TCGA-12-1597 | 675 | Dead | 60-69 | Female | Yes | Yes | Yes | Proneural |
| TCGA-26-1442 | 953 | Alive | < 50 | Male | Yes | Yes | Yes | Proneural |
| TCGA-27-2521 | 316 | Alive | < 50 | Male | Yes | Yes | Yes | Proneural |
| TCGA-06-0686 | 286 | Alive | 50-59 | Male | Yes | Yes | Yes | Proneural |
| TCGA-19-5960 | 165 | Alive | 50-59 | Male | Yes | Yes | Yes | Proneural |
| TCGA-26-5133 | 452 | Alive | 50-59 | Male | Yes | Yes | Yes | Proneural |
| TCGA-32-5222 | 165 | Alive | 60-69 | Male | Yes | Yes | Yes | Proneural |
| TCGA-06-0238 | 405 | Dead | < 50 | Male | Yes | Yes | Yes | Proneural |
| TCGA-12-0618 | 395 | Dead | < 50 | Male | Yes | Yes | Yes | Proneural |
| TCGA-12-3650 | 333 | Dead | < 50 | Male | Yes | Yes | Yes | Proneural |
| TCGA-27-1830 | 154 | Dead | 50-59 | Male | Yes | Yes | Yes | Proneural |
| TCGA-06-0646 | 175 | Dead | 60-69 | Male | Yes | Yes | Yes | Proneural |
| TCGA-19-2629 | 737 | Dead | 60-69 | Male | Yes | Yes | Yes | Proneural |
| TCGA-06-2559 | 150 | Dead | > 80 | Male | Yes | Yes | Yes | Proneural |
