# Supplementary material for: Investigation of Genetic Determinants of Glioma Immune Phenotype by Integrative Immunogenomic Scale Analysis
Source: Front Immunol. 2021 Jun 16;12:557994. doi: 10.3389/fimmu.2021.557994 (PMC8242587; doi:10.3389/fimmu.2021.557994)
Supplement: Supplementary file 4 [file Table_3.docx]

**Supplementary Online File 3. Clinical data for CGGA microarray samples**

| ID | OS (day) | Survival status | Histology subtype | PRS type | Histology | Grade | Gender | Age | Radiation | Pharmaceutical therapy | IDH mutation | 1p19q codeletion status |
| --- | --- | --- | --- | --- | --- | --- | --- | --- | --- | --- | --- | --- |
| CGGA_406 | 90 | Dead | Classical | Recurrent | rLGG | WHO III | Female | < 50 | No | No | IDH1 Mutant | Non-codel |
| CGGA_1068 | 68 | Dead | Mesenchymal | Secondary | sGBM | WHO IV | Male | < 50 | No | No | IDH1 Mutant | Non-codel |
| CGGA_267 | 1374 | Dead | Neural | Primary | LGG | WHO II | Male | < 50 | No | No | IDH1 Mutant | Non-codel |
| CGGA_1059 | 21 | Dead | Proneural | Recurrent | rLGG | WHO II | Female | 50-59 | No | No | IDH1 Mutant | Non-codel |
| CGGA_662 | 284 | Dead | Proneural | Primary | LGG | WHO II | Female | 50-59 | No | No | IDH1 Mutant | Codel |
| CGGA_718 | 3829 | Alive | Mesenchymal | Primary | LGG | WHO II | Male | < 50 | Yes | No | IDH1 Mutant | NA |
| CGGA_672 | 3885 | Alive | Proneural | Primary | LGG | WHO II | Male | < 50 | Yes | No | IDH1 Mutant | NA |
| CGGA_522 | 3630 | Alive | Neural | Primary | LGG | WHO II | Female | < 50 | Yes | No | IDH1 Mutant | NA |
| CGGA_663 | 3880 | Alive | Neural | Primary | LGG | WHO II | Female | < 50 | Yes | No | IDH1 Mutant | Non-codel |
| CGGA_316 | 4347 | Alive | Proneural | Primary | LGG | WHO II | Female | < 50 | Yes | No | IDH1 Mutant | NA |
| CGGA_469 | 4138 | Alive | Neural | Primary | LGG | WHO II | Male | < 50 | Yes | No | IDH1 Mutant | NA |
| CGGA_243 | 2977 | Alive | Proneural | Primary | LGG | WHO II | Female | < 50 | Yes | No | IDH1 Mutant | Codel |
| CGGA_441 | 4159 | Alive | Proneural | Primary | LGG | WHO II | Female | < 50 | Yes | No | IDH1 Mutant | NA |
| CGGA_484 | 4116 | Alive | Neural | Primary | LGG | WHO II | Female | < 50 | Yes | No | IDH1 Mutant | NA |
| CGGA_511 | 3770 | Alive | Neural | Primary | LGG | WHO II | Male | < 50 | Yes | No | IDH1 Mutant | NA |
| CGGA_407 | 4220 | Alive | Classical | Primary | LGG | WHO II | Male | < 50 | Yes | No | IDH1 Mutant | NA |
| CGGA_589 | 3991 | Alive | Proneural | Primary | LGG | WHO II | Male | < 50 | Yes | No | IDH1 Mutant | NA |
| CGGA_315 | 4033 | Alive | Neural | Primary | LGG | WHO II | Female | < 50 | Yes | No | IDH1 Mutant | NA |
| CGGA_760 | 3780 | Alive | Neural | Primary | LGG | WHO II | Female | < 50 | Yes | No | IDH1 Mutant | Codel |
| CGGA_765 | 3773 | Alive | Proneural | Primary | LGG | WHO II | Male | < 50 | Yes | No | IDH1 Mutant | NA |
| CGGA_434 | 4178 | Alive | Mesenchymal | Primary | LGG | WHO II | Male | < 50 | Yes | No | IDH1 Mutant | NA |
| CGGA_446 | 4173 | Alive | Proneural | Primary | LGG | WHO II | Female | < 50 | Yes | No | IDH1 Mutant | Codel |
| CGGA_590 | 3990 | Alive | Proneural | Primary | LGG | WHO II | Male | < 50 | Yes | No | IDH1 Mutant | NA |
| CGGA_411 | 4217 | Alive | Proneural | Primary | LGG | WHO II | Male | < 50 | Yes | No | IDH1 Mutant | NA |
| CGGA_671 | 3885 | Alive | Neural | Primary | LGG | WHO II | Female | < 50 | Yes | No | IDH1 Mutant | Codel |
| CGGA_253 | 4824 | Alive | Proneural | Primary | LGG | WHO II | Female | < 50 | Yes | No | IDH1 Mutant | NA |
| CGGA_868 | 3640 | Alive | Classical | Primary | LGG | WHO II | Male | < 50 | Yes | No | IDH1 Mutant | NA |
| CGGA_519 | 4070 | Alive | Neural | Primary | LGG | WHO II | Female | < 50 | Yes | No | IDH1 Mutant | NA |
| CGGA_708 | 3837 | Alive | Proneural | Primary | LGG | WHO II | Female | < 50 | Yes | No | IDH1 Mutant | NA |
| CGGA_502 | 4088 | Alive | Proneural | Primary | LGG | WHO II | Male | < 50 | Yes | No | IDH1 Mutant | Non-codel |
| CGGA_615 | 3957 | Alive | Proneural | Primary | LGG | WHO II | Female | < 50 | Yes | No | IDH1 Mutant | NA |
| CGGA_1005 | 3426 | Alive | Neural | Primary | LGG | WHO II | Female | < 50 | Yes | No | IDH1 Mutant | Non-codel |
| CGGA_560 | 4025 | Alive | Proneural | Primary | LGG | WHO III | Male | < 50 | Yes | No | IDH1 Mutant | Non-codel |
| CGGA_543 | 4042 | Alive | Proneural | Primary | LGG | WHO II | Male | < 50 | Yes | No | IDH1 Mutant | Codel |
| CGGA_459 | 3613 | Alive | Mesenchymal | Primary | LGG | WHO II | Male | < 50 | Yes | No | IDH1 Mutant | NA |
| CGGA_251 | 1299 | Alive | Neural | Primary | LGG | WHO II | Male | < 50 | Yes | No | IDH1 Mutant | Non-codel |
| CGGA_623 | 2407 | Alive | Neural | Primary | LGG | WHO II | Male | < 50 | Yes | No | IDH1 Mutant | NA |
| CGGA_639 | 3927 | Alive | Neural | Primary | LGG | WHO II | Male | < 50 | Yes | No | IDH1 Mutant | NA |
| CGGA_776 | 3761 | Alive | Proneural | Primary | LGG | WHO II | Female | < 50 | Yes | No | IDH1 Mutant | NA |
| CGGA_402 | 4227 | Alive | Neural | Primary | LGG | WHO III | Male | 50-59 | Yes | No | IDH1 Mutant | NA |
| CGGA_523 | 4063 | Alive | Neural | Primary | LGG | WHO II | Female | 60-69 | Yes | No | IDH1 Mutant | NA |
| CGGA_508 | 285 | Dead | Classical | Primary | LGG | WHO III | Female | < 50 | Yes | No | IDH1 Mutant | NA |
| CGGA_518 | 212 | Dead | Neural | Secondary | sGBM | WHO IV | Male | < 50 | Yes | No | IDH1 Mutant | NA |
| CGGA_714 | 400 | Dead | Proneural | Primary | GBM | WHO IV | Female | < 50 | Yes | No | IDH1 Mutant | NA |
| CGGA_743 | 851 | Dead | Mesenchymal | Primary | LGG | WHO II | Male | < 50 | Yes | No | IDH1 Mutant | NA |
| CGGA_778 | 175 | Dead | Neural | Primary | GBM | WHO IV | Female | < 50 | Yes | No | IDH1 Mutant | NA |
| CGGA_740 | 766 | Dead | Proneural | Primary | LGG | WHO II | Male | < 50 | Yes | No | IDH1 Mutant | NA |
| CGGA_792 | 816 | Dead | Proneural | Primary | LGG | WHO II | Male | < 50 | Yes | No | IDH1 Mutant | NA |
| CGGA_273 | 2635 | Dead | Proneural | Primary | LGG | WHO II | Female | < 50 | Yes | No | IDH1 Mutant | Non-codel |
| CGGA_461 | 1266 | Dead | Neural | Primary | LGG | WHO II | Male | < 50 | Yes | No | IDH1 Mutant | NA |
| CGGA_505 | 2994 | Dead | Mesenchymal | Primary | LGG | WHO II | Male | < 50 | Yes | No | IDH1 Mutant | NA |
| CGGA_583 | 3107 | Dead | Proneural | Primary | LGG | WHO II | Female | < 50 | Yes | No | IDH1 Mutant | NA |
| CGGA_601 | 1133 | Dead | Neural | Primary | LGG | WHO II | Male | < 50 | Yes | No | IDH1 Mutant | Non-codel |
| CGGA_379 | 1077 | Dead | Mesenchymal | Primary | LGG | WHO II | Female | < 50 | Yes | No | IDH1 Mutant | NA |
| CGGA_311 | 230 | Dead | Neural | Primary | GBM | WHO IV | Male | < 50 | Yes | No | IDH1 Mutant | NA |
| CGGA_798 | 136 | Dead | Mesenchymal | Secondary | sGBM | WHO IV | Female | < 50 | Yes | No | IDH1 Mutant | NA |
| CGGA_282 | 247 | Dead | Mesenchymal | Primary | LGG | WHO II | Male | < 50 | Yes | No | IDH1 Mutant | NA |
| CGGA_654 | 626 | Dead | Mesenchymal | Primary | GBM | WHO IV | Male | < 50 | Yes | No | IDH1 Mutant | NA |
| CGGA_761 | 1840 | Dead | Proneural | Primary | GBM | WHO IV | Male | < 50 | Yes | No | IDH1 Mutant | Non-codel |
| CGGA_260 | 567 | Dead | Proneural | Primary | LGG | WHO III | Female | < 50 | Yes | No | IDH1 Mutant | NA |
| CGGA_437 | 1025 | Dead | Mesenchymal | Primary | GBM | WHO IV | Female | 50-59 | Yes | No | IDH1 Mutant | NA |
| CGGA_353 | 516 | Dead | Neural | Primary | LGG | WHO III | Female | 60-69 | Yes | No | IDH1 Mutant | NA |
| CGGA_1046-1 | 3170 | Alive | Neural | Primary | LGG | WHO II | Male | < 50 | No | No | IDH1 Mutant | NA |
| CGGA_D46 | 300 | Dead | Proneural | Recurrent | rLGG | WHO III | Female | < 50 | No | Yes | IDH1 Mutant | Non-codel |
| CGGA_D60 | 196 | Dead | Proneural | Secondary | sGBM | WHO IV | Male | < 50 | No | Yes | IDH1 Mutant | Non-codel |
| CGGA_1060 | 227 | Dead | Proneural | Recurrent | rGBM | WHO IV | Male | < 50 | No | Yes | IDH1 Mutant | Non-codel |
| CGGA_899 | 231 | Dead | Proneural | Recurrent | rGBM | WHO IV | Male | < 50 | No | Yes | IDH1 Mutant | Non-codel |
| CGGA_700 | 170 | Alive | Mesenchymal | Primary | GBM | WHO IV | Female | < 50 | Yes | Yes | IDH1 Mutant | Non-codel |
| CGGA_489 | 3804 | Alive | Mesenchymal | Primary | LGG | WHO III | Male | < 50 | Yes | Yes | IDH1 Mutant | NA |
| CGGA_485 | 2437 | Alive | Neural | Primary | LGG | WHO II | Male | < 50 | Yes | Yes | IDH1 Mutant | Codel |
| CGGA_365 | 3593 | Alive | Neural | Primary | LGG | WHO II | Female | < 50 | Yes | Yes | IDH1 Mutant | NA |
| CGGA_558 | 188 | Alive | Neural | Primary | LGG | WHO III | Male | < 50 | Yes | Yes | IDH1 Mutant | NA |
| CGGA_281 | 1236 | Alive | Neural | Primary | LGG | WHO II | Female | < 50 | Yes | Yes | IDH1 Mutant | NA |
| CGGA_490 | 3804 | Alive | Proneural | Primary | LGG | WHO III | Female | < 50 | Yes | Yes | IDH1 Mutant | Codel |
| CGGA_1027 | 2688 | Alive | Proneural | Primary | LGG | WHO III | Male | < 50 | Yes | Yes | IDH1 Mutant | Codel |
| CGGA_256 | 158 | Alive | Neural | Primary | LGG | WHO II | Male | < 50 | Yes | Yes | IDH1 Mutant | Non-codel |
| CGGA_420 | 3514 | Alive | Proneural | Recurrent | rLGG | WHO III | Female | < 50 | Yes | Yes | IDH1 Mutant | Codel |
| CGGA_277 | 4404 | Alive | Neural | Primary | LGG | WHO III | Male | < 50 | Yes | Yes | IDH1 Mutant | NA |
| CGGA_317 | 4346 | Alive | Proneural | Primary | LGG | WHO II | Female | < 50 | Yes | Yes | IDH1 Mutant | NA |
| CGGA_664 | 3894 | Alive | Proneural | Primary | GBM | WHO IV | Female | < 50 | Yes | Yes | IDH1 Mutant | NA |
| CGGA_403 | 925 | Alive | Proneural | Primary | LGG | WHO III | Male | < 50 | Yes | Yes | IDH1 Mutant | NA |
| CGGA_348 | 4310 | Alive | Proneural | Primary | LGG | WHO II | Female | < 50 | Yes | Yes | IDH1 Mutant | NA |
| CGGA_605 | 3971 | Alive | Mesenchymal | Recurrent | rLGG | WHO III | Male | < 50 | Yes | Yes | IDH1 Mutant | Non-codel |
| CGGA_544 | 4042 | Alive | Proneural | Primary | LGG | WHO II | Female | < 50 | Yes | Yes | IDH1 Mutant | NA |
| CGGA_635 | 3934 | Alive | Neural | Primary | LGG | WHO II | Male | < 50 | Yes | Yes | IDH1 Mutant | Codel |
| CGGA_592 | 3986 | Alive | Proneural | Primary | LGG | WHO II | Male | < 50 | Yes | Yes | IDH1 Mutant | NA |
| CGGA_711 | 1798 | Alive | Neural | Primary | LGG | WHO II | Male | < 50 | Yes | Yes | IDH1 Mutant | NA |
| CGGA_378 | 3147 | Alive | Proneural | Primary | LGG | WHO II | Male | 50-59 | Yes | Yes | IDH1 Mutant | NA |
| CGGA_753 | 3217 | Dead | Proneural | Primary | LGG | WHO II | Male | < 50 | Yes | Yes | IDH1 Mutant | NA |
| CGGA_809 | 1252 | Dead | Proneural | Primary | LGG | WHO II | Male | < 50 | Yes | Yes | IDH1 Mutant | Non-codel |
| CGGA_547 | 1337 | Dead | Mesenchymal | Primary | GBM | WHO IV | Male | < 50 | Yes | Yes | IDH1 Mutant | NA |
| CGGA_747 | 970 | Dead | Proneural | Primary | GBM | WHO IV | Male | < 50 | Yes | Yes | IDH1 Mutant | Non-codel |
| CGGA_391 | 2010 | Dead | Mesenchymal | Primary | LGG | WHO III | Female | < 50 | Yes | Yes | IDH1 Mutant | NA |
| CGGA_674 | 1132 | Dead | Neural | Primary | LGG | WHO II | Male | < 50 | Yes | Yes | IDH1 Mutant | NA |
| CGGA_703 | 1355 | Dead | Neural | Recurrent | rLGG | WHO III | Female | < 50 | Yes | Yes | IDH1 Mutant | Non-codel |
| CGGA_364 | 666 | Dead | Proneural | Primary | LGG | WHO III | Male | < 50 | Yes | Yes | IDH1 Mutant | NA |
| CGGA_439 | 3813 | Dead | Mesenchymal | Primary | GBM | WHO IV | Female | < 50 | Yes | Yes | IDH1 Mutant | NA |
| CGGA_648 | 3647 | Dead | Proneural | Primary | LGG | WHO II | Female | < 50 | Yes | Yes | IDH1 Mutant | NA |
| CGGA_549 | 413 | Dead | Neural | Primary | GBM | WHO IV | Female | < 50 | Yes | Yes | IDH1 Mutant | NA |
| CGGA_312 | 740 | Dead | Proneural | Primary | LGG | WHO II | Female | < 50 | Yes | Yes | IDH1 Mutant | NA |
| CGGA_694 | 1194 | Dead | Mesenchymal | Recurrent | rLGG | WHO II | Male | < 50 | Yes | Yes | IDH1 Mutant | Non-codel |
| CGGA_491 | 1074 | Dead | Proneural | Primary | GBM | WHO IV | Female | < 50 | Yes | Yes | IDH1 Mutant | Non-codel |
| CGGA_399 | 2663 | Dead | Proneural | Primary | LGG | WHO II | Male | < 50 | Yes | Yes | IDH1 Mutant | NA |
| CGGA_704 | 2572 | Dead | Proneural | Primary | LGG | WHO II | Male | < 50 | Yes | Yes | IDH1 Mutant | Non-codel |
| CGGA_357 | 2518 | Dead | Proneural | Primary | LGG | WHO II | Female | < 50 | Yes | Yes | IDH1 Mutant | NA |
| CGGA_498 | 356 | Dead | Neural | Primary | LGG | WHO III | Male | < 50 | Yes | Yes | IDH1 Mutant | NA |
| CGGA_374 | 493 | Dead | Proneural | Secondary | sGBM | WHO IV | Male | < 50 | Yes | Yes | IDH1 Mutant | Non-codel |
| CGGA_736 | 1164 | Dead | Proneural | Primary | LGG | WHO II | Male | < 50 | Yes | Yes | IDH1 Mutant | NA |
| CGGA_314 | 2568 | Dead | Neural | Recurrent | rLGG | WHO III | Male | < 50 | Yes | Yes | IDH1 Mutant | Codel |
| CGGA_412 | 358 | Dead | Neural | Primary | LGG | WHO III | Female | < 50 | Yes | Yes | IDH1 Mutant | NA |
| CGGA_652 | 476 | Dead | Neural | Recurrent | rLGG | WHO III | Male | < 50 | Yes | Yes | IDH1 Mutant | Non-codel |
| CGGA_1061 | 532 | Dead | Proneural | Recurrent | rLGG | WHO III | Female | < 50 | Yes | Yes | IDH1 Mutant | Non-codel |
| CGGA_482 | 2511 | Dead | Neural | Primary | LGG | WHO II | Male | < 50 | Yes | Yes | IDH1 Mutant | Codel |
| CGGA_822 | 2237 | Dead | Proneural | Secondary | sGBM | WHO IV | Male | < 50 | Yes | Yes | IDH1 Mutant | Non-codel |
| CGGA_387 | 539 | Dead | Neural | Primary | LGG | WHO II | Female | 50-59 | Yes | Yes | IDH1 Mutant | NA |
| CGGA_438 | 686 | Dead | Mesenchymal | Primary | LGG | WHO III | Male | 50-59 | Yes | Yes | IDH1 Mutant | NA |
| CGGA_405 | 497 | Dead | Proneural | Primary | LGG | WHO III | Male | 50-59 | Yes | Yes | IDH1 Mutant | NA |
| CGGA_598 | 3677 | Dead | Proneural | Primary | LGG | WHO III | Female | 60-69 | Yes | Yes | IDH1 Mutant | Codel |
| CGGA_442 | 1555 | Dead | Proneural | Primary | GBM | WHO IV | Male | 60-69 | Yes | Yes | IDH1 Mutant | NA |
| CGGA_354 | 180 | Dead | Neural | Primary | LGG | WHO III | Male | 60-69 | Yes | Yes | IDH1 Mutant | NA |
| CGGA_393 | 2190 | Dead | Proneural | Primary | LGG | WHO III | Male | 60-69 | Yes | Yes | IDH1 Mutant | NA |
| CGGA_447 | 4173 | Alive | Mesenchymal | Primary | LGG | WHO II | Female | < 50 | No | NA | IDH1 Mutant | NA |
| CGGA_712 | 1111 | Alive | Proneural | Primary | LGG | WHO II | Male | < 50 | No | NA | IDH1 Mutant | NA |
| CGGA_548 | 177 | Alive | Proneural | Primary | LGG | WHO II | Male | < 50 | No | NA | IDH1 Mutant | NA |
| CGGA_595 | 1390 | Alive | Neural | Primary | LGG | WHO II | Female | 50-59 | No | NA | IDH1 Mutant | NA |
| CGGA_191 | 3036 | Dead | Neural | Primary | LGG | WHO II | Male | 50-59 | No | NA | IDH1 Mutant | NA |
| CGGA_835 | 517 | Alive | Neural | Primary | LGG | WHO II | Male | < 50 | Yes | NA | IDH1 Mutant | Codel |
| CGGA_856 | 1297 | Alive | Proneural | Primary | LGG | WHO II | Male | 50-59 | Yes | NA | IDH1 Mutant | Codel |
| CGGA_898 | 1663 | Dead | Neural | Primary | LGG | WHO II | Male | < 50 | Yes | NA | IDH1 Mutant | Non-codel |
| CGGA_D39 | 355 | Alive | Proneural | Recurrent | rLGG | WHO II | Female | < 50 | NA | NA | IDH1 Mutant | Non-codel |
| CGGA_285 | 1576 | Alive | Proneural | Primary | LGG | WHO II | Male | < 50 | NA | NA | IDH1 Mutant | NA |
| CGGA_433 | 1444 | Alive | Neural | Primary | LGG | WHO II | Male | < 50 | NA | NA | IDH1 Mutant | NA |
| CGGA_571 | 1289 | Alive | Proneural | Primary | LGG | WHO II | Male | < 50 | NA | NA | IDH1 Mutant | NA |
| CGGA_1069 | NA | NA | Mesenchymal | Recurrent | rLGG | WHO II | Male | < 50 | NA | NA | IDH1 Mutant | Non-codel |
| CGGA_767 | 1310 | Dead | Mesenchymal | Primary | LGG | WHO III | Male | < 50 | Yes | Yes | NA | NA |
| CGGA_692 | 157 | Alive | Neural | Primary | LGG | WHO III | Male | < 50 | No | NA | NA | NA |
| CGGA_626 | 3944 | Alive | Neural | Primary | LGG | WHO II | Male | < 50 | No | No | IDH WT | NA |
| CGGA_610 | 3549 | Alive | Proneural | Primary | LGG | WHO II | Female | < 50 | No | No | IDH WT | NA |
| CGGA_104 | 440 | Alive | Classical | Secondary | sGBM | WHO IV | Male | < 50 | No | No | IDH WT | NA |
| CGGA_323 | 4338 | Alive | Neural | Primary | LGG | WHO II | Female | < 50 | No | No | IDH WT | Non-codel |
| CGGA_850 | 564 | Alive | Classical | Primary | GBM | WHO IV | Female | 50-59 | No | No | IDH WT | Non-codel |
| CGGA_713 | 27 | Dead | Mesenchymal | Primary | GBM | WHO IV | Male | < 50 | No | No | IDH WT | NA |
| CGGA_706 | 619 | Dead | Neural | Primary | LGG | WHO II | Male | < 50 | No | No | IDH WT | NA |
| CGGA_566 | 1653 | Dead | Proneural | Primary | LGG | WHO II | Male | < 50 | No | No | IDH WT | NA |
| CGGA_681 | 110 | Dead | Mesenchymal | NA | NA | NA | Male | < 50 | No | No | IDH WT | NA |
| CGGA_373 | 281 | Dead | Mesenchymal | Primary | GBM | WHO IV | Female | < 50 | No | No | IDH WT | NA |
| CGGA_746 | 332 | Dead | Mesenchymal | Primary | LGG | WHO II | Male | < 50 | No | No | IDH WT | NA |
| CGGA_495 | 295 | Dead | Mesenchymal | NA | NA | NA | Male | 50-59 | No | No | IDH WT | NA |
| CGGA_419 | 198 | Dead | Mesenchymal | Primary | GBM | WHO IV | Male | 50-59 | No | No | IDH WT | NA |
| CGGA_649 | 147 | Dead | Neural | Primary | GBM | WHO IV | Female | 50-59 | No | No | IDH WT | NA |
| CGGA_444 | 225 | Dead | Neural | Primary | GBM | WHO IV | Female | > 70 | No | No | IDH WT | NA |
| CGGA_688 | 3866 | Alive | Mesenchymal | Primary | LGG | WHO II | Male | < 50 | Yes | No | IDH WT | NA |
| CGGA_398 | 3115 | Alive | Neural | Primary | LGG | WHO II | Male | < 50 | Yes | No | IDH WT | NA |
| CGGA_770 | 1057 | Alive | Neural | Primary | LGG | WHO II | Male | < 50 | Yes | No | IDH WT | NA |
| CGGA_500 | 1520 | Alive | Proneural | Primary | LGG | WHO II | Female | < 50 | Yes | No | IDH WT | NA |
| CGGA_266 | 3328 | Alive | Mesenchymal | Primary | LGG | WHO II | Male | < 50 | Yes | No | IDH WT | NA |
| CGGA_275 | 4387 | Alive | Neural | Primary | LGG | WHO II | Female | < 50 | Yes | No | IDH WT | NA |
| CGGA_440 | 4159 | Alive | Neural | Primary | LGG | WHO II | Male | < 50 | Yes | No | IDH WT | NA |
| CGGA_455 | 4159 | Alive | Proneural | Primary | LGG | WHO III | Female | < 50 | Yes | No | IDH WT | NA |
| CGGA_296 | 198 | Alive | Neural | Primary | LGG | WHO II | Male | < 50 | Yes | No | IDH WT | NA |
| CGGA_396 | 4241 | Alive | Proneural | Primary | LGG | WHO II | Female | < 50 | Yes | No | IDH WT | NA |
| CGGA_291 | 1736 | Alive | Neural | Primary | LGG | WHO II | Female | 50-59 | Yes | No | IDH WT | NA |
| CGGA_640 | 3922 | Alive | Mesenchymal | Primary | GBM | WHO IV | Male | 50-59 | Yes | No | IDH WT | NA |
| CGGA_683 | 688 | Alive | Mesenchymal | Primary | GBM | WHO IV | Female | 50-59 | Yes | No | IDH WT | NA |
| CGGA_570 | 3602 | Alive | Proneural | Primary | GBM | WHO IV | Female | 60-69 | Yes | No | IDH WT | NA |
| CGGA_415 | 182 | Alive | Neural | Primary | LGG | WHO II | Male | NA | Yes | No | IDH WT | NA |
| CGGA_504 | 563 | Dead | Classical | Primary | GBM | WHO IV | Female | < 50 | Yes | No | IDH WT | NA |
| CGGA_347 | 3900 | Dead | Neural | Primary | LGG | WHO II | Female | < 50 | Yes | No | IDH WT | NA |
| CGGA_334 | 1450 | Dead | Neural | Primary | LGG | WHO III | Female | < 50 | Yes | No | IDH WT | NA |
| CGGA_221 | 287 | Dead | Mesenchymal | Primary | GBM | WHO IV | Male | < 50 | Yes | No | IDH WT | NA |
| CGGA_557 | 257 | Dead | Mesenchymal | Primary | GBM | WHO IV | Female | < 50 | Yes | No | IDH WT | NA |
| CGGA_464 | 403 | Dead | Mesenchymal | Primary | GBM | WHO IV | Female | < 50 | Yes | No | IDH WT | NA |
| CGGA_203 | 188 | Dead | Mesenchymal | Primary | GBM | WHO IV | Male | < 50 | Yes | No | IDH WT | NA |
| CGGA_579 | 2125 | Dead | Neural | Primary | LGG | WHO II | Female | < 50 | Yes | No | IDH WT | NA |
| CGGA_1011 | 109 | Dead | Mesenchymal | Primary | GBM | WHO IV | Female | < 50 | Yes | No | IDH WT | Non-codel |
| CGGA_436 | 955 | Dead | Mesenchymal | Primary | GBM | WHO IV | Male | < 50 | Yes | No | IDH WT | NA |
| CGGA_686 | 348 | Dead | Mesenchymal | Primary | GBM | WHO IV | Male | 50-59 | Yes | No | IDH WT | NA |
| CGGA_731 | 503 | Dead | Mesenchymal | Primary | GBM | WHO IV | Male | 50-59 | Yes | No | IDH WT | Non-codel |
| CGGA_764 | 67 | Dead | Mesenchymal | Primary | GBM | WHO IV | Male | 50-59 | Yes | No | IDH WT | NA |
| CGGA_370 | 338 | Dead | Neural | Primary | GBM | WHO IV | Male | 50-59 | Yes | No | IDH WT | NA |
| CGGA_499 | 122 | Dead | Proneural | Primary | GBM | WHO IV | Male | 50-59 | Yes | No | IDH WT | Non-codel |
| CGGA_528 | 2424 | Dead | Proneural | Primary | LGG | WHO II | Female | 50-59 | Yes | No | IDH WT | NA |
| CGGA_272 | 217 | Dead | Mesenchymal | Secondary | sGBM | WHO IV | Male | 50-59 | Yes | No | IDH WT | Non-codel |
| CGGA_514 | 2022 | Dead | Proneural | Primary | LGG | WHO III | Female | 50-59 | Yes | No | IDH WT | NA |
| CGGA_264 | 383 | Dead | Proneural | Primary | GBM | WHO IV | Male | 50-59 | Yes | No | IDH WT | NA |
| CGGA_395 | 1551 | Dead | Proneural | Primary | LGG | WHO II | Male | 50-59 | Yes | No | IDH WT | NA |
| CGGA_859 | 387 | Dead | Mesenchymal | Primary | GBM | WHO IV | Male | 50-59 | Yes | No | IDH WT | Non-codel |
| CGGA_734 | 466 | Dead | Classical | Primary | GBM | WHO IV | Female | 50-59 | Yes | No | IDH WT | NA |
| CGGA_11 | 155 | Dead | Classical | Primary | GBM | WHO IV | Female | 50-59 | Yes | No | IDH WT | NA |
| CGGA_741 | 396 | Dead | Mesenchymal | Primary | GBM | WHO IV | Female | 50-59 | Yes | No | IDH WT | NA |
| CGGA_786 | 1234 | Dead | Neural | Primary | LGG | WHO II | Male | 50-59 | Yes | No | IDH WT | NA |
| CGGA_335 | 385 | Dead | Proneural | Primary | GBM | WHO IV | Male | 50-59 | Yes | No | IDH WT | NA |
| CGGA_782 | 289 | Dead | Mesenchymal | Primary | GBM | WHO IV | Male | 60-69 | Yes | No | IDH WT | Non-codel |
| CGGA_231 | 510 | Dead | Classical | Primary | LGG | WHO III | Male | 60-69 | Yes | No | IDH WT | NA |
| CGGA_562 | 290 | Dead | Mesenchymal | Primary | LGG | WHO III | Female | 60-69 | Yes | No | IDH WT | NA |
| CGGA_1015 | 164 | Dead | Mesenchymal | Primary | GBM | WHO IV | Male | 60-69 | Yes | No | IDH WT | Non-codel |
| CGGA_679 | 263 | Dead | Mesenchymal | Primary | GBM | WHO IV | Female | 60-69 | Yes | No | IDH WT | Non-codel |
| CGGA_729 | 687 | Dead | Mesenchymal | Primary | GBM | WHO IV | Female | 60-69 | Yes | No | IDH WT | NA |
| CGGA_775 | 255 | Dead | Mesenchymal | Primary | GBM | WHO IV | Male | 60-69 | Yes | No | IDH WT | NA |
| CGGA_646 | 363 | Dead | Mesenchymal | Primary | GBM | WHO IV | Female | 60-69 | Yes | No | IDH WT | NA |
| CGGA_1022 | 518 | Alive | Mesenchymal | Recurrent | rLGG | WHO II | Female | 60-69 | No | Yes | IDH WT | Non-codel |
| CGGA_628 | 480 | Dead | Mesenchymal | Primary | GBM | WHO IV | Male | < 50 | No | Yes | IDH WT | NA |
| CGGA_1039 | 727 | Dead | Classical | Primary | GBM | WHO IV | Male | < 50 | No | Yes | IDH WT | Non-codel |
| CGGA_530 | 506 | Dead | Neural | Recurrent | rGBM | WHO IV | Female | < 50 | No | Yes | IDH WT | Non-codel |
| CGGA_588 | 773 | Dead | Mesenchymal | Primary | GBM | WHO IV | Male | 50-59 | No | Yes | IDH WT | NA |
| CGGA_168 | 3086 | Alive | Mesenchymal | Primary | GBM | WHO IV | Male | < 50 | Yes | Yes | IDH WT | NA |
| CGGA_352 | 4304 | Alive | Neural | Primary | LGG | WHO III | Male | < 50 | Yes | Yes | IDH WT | NA |
| CGGA_259 | 649 | Alive | Proneural | Recurrent | rLGG | WHO III | Female | < 50 | Yes | Yes | IDH WT | NA |
| CGGA_594 | 3989 | Alive | Mesenchymal | Primary | GBM | WHO IV | Female | < 50 | Yes | Yes | IDH WT | NA |
| CGGA_492 | 652 | Alive | Mesenchymal | Recurrent | rLGG | WHO III | Male | < 50 | Yes | Yes | IDH WT | Non-codel |
| CGGA_512 | 4045 | Alive | Neural | Primary | GBM | WHO IV | Male | < 50 | Yes | Yes | IDH WT | NA |
| CGGA_552 | 4033 | Alive | Mesenchymal | Primary | LGG | WHO II | Female | < 50 | Yes | Yes | IDH WT | NA |
| CGGA_541 | 4047 | Alive | Neural | Primary | LGG | WHO II | Female | < 50 | Yes | Yes | IDH WT | Non-codel |
| CGGA_577 | 3901 | Alive | Neural | Primary | LGG | WHO III | Female | < 50 | Yes | Yes | IDH WT | NA |
| CGGA_271 | 1603 | Alive | Neural | Primary | LGG | WHO II | Male | < 50 | Yes | Yes | IDH WT | NA |
| CGGA_345 | 1547 | Alive | Classical | Primary | GBM | WHO IV | Female | < 50 | Yes | Yes | IDH WT | NA |
| CGGA_609 | 512 | Alive | Classical | Primary | GBM | WHO IV | Female | < 50 | Yes | Yes | IDH WT | NA |
| CGGA_418 | 4201 | Alive | Neural | Primary | LGG | WHO III | Male | < 50 | Yes | Yes | IDH WT | NA |
| CGGA_659 | 730 | Alive | Neural | Primary | LGG | WHO II | Female | 50-59 | Yes | Yes | IDH WT | Non-codel |
| CGGA_848 | 239 | Alive | Mesenchymal | Primary | GBM | WHO IV | Female | 50-59 | Yes | Yes | IDH WT | Non-codel |
| CGGA_413 | 183 | Alive | Mesenchymal | Primary | GBM | WHO IV | Male | 50-59 | Yes | Yes | IDH WT | Non-codel |
| CGGA_474 | 2029 | Alive | Classical | Primary | LGG | WHO III | Male | NA | Yes | Yes | IDH WT | Non-codel |
| CGGA_218 | 313 | Dead | Classical | Primary | GBM | WHO IV | Male | < 50 | Yes | Yes | IDH WT | NA |
| CGGA_377 | 398 | Dead | Classical | Primary | GBM | WHO IV | Male | < 50 | Yes | Yes | IDH WT | NA |
| CGGA_401 | 168 | Dead | Classical | Primary | GBM | WHO IV | Female | < 50 | Yes | Yes | IDH WT | NA |
| CGGA_331 | 1638 | Dead | Mesenchymal | Primary | LGG | WHO III | Female | < 50 | Yes | Yes | IDH WT | NA |
| CGGA_232 | 415 | Dead | Mesenchymal | Recurrent | rLGG | WHO III | Male | < 50 | Yes | Yes | IDH WT | NA |
| CGGA_1081 | 216 | Dead | Proneural | Recurrent | rLGG | WHO III | Female | < 50 | Yes | Yes | IDH WT | Non-codel |
| CGGA_597 | 733 | Dead | Proneural | Primary | GBM | WHO IV | Female | < 50 | Yes | Yes | IDH WT | NA |
| CGGA_225 | 1741 | Dead | Proneural | Primary | GBM | WHO IV | Male | < 50 | Yes | Yes | IDH WT | NA |
| CGGA_709 | 406 | Dead | Mesenchymal | Primary | GBM | WHO IV | Female | < 50 | Yes | Yes | IDH WT | NA |
| CGGA_527 | 439 | Dead | Classical | Primary | GBM | WHO IV | Male | < 50 | Yes | Yes | IDH WT | NA |
| CGGA_699 | 570 | Dead | Mesenchymal | Primary | GBM | WHO IV | Female | < 50 | Yes | Yes | IDH WT | NA |
| CGGA_573 | 946 | Dead | Mesenchymal | Primary | GBM | WHO IV | Male | < 50 | Yes | Yes | IDH WT | NA |
| CGGA_351 | 742 | Dead | Mesenchymal | Primary | LGG | WHO III | Female | < 50 | Yes | Yes | IDH WT | NA |
| CGGA_454 | 412 | Dead | Mesenchymal | Primary | GBM | WHO IV | Male | < 50 | Yes | Yes | IDH WT | NA |
| CGGA_593 | 242 | Dead | Mesenchymal | Primary | GBM | WHO IV | Male | < 50 | Yes | Yes | IDH WT | NA |
| CGGA_476 | 936 | Dead | Mesenchymal | Primary | LGG | WHO II | Female | < 50 | Yes | Yes | IDH WT | NA |
| CGGA_178 | 972 | Dead | Mesenchymal | Primary | GBM | WHO IV | Male | < 50 | Yes | Yes | IDH WT | NA |
| CGGA_462 | 361 | Dead | Neural | Secondary | sGBM | WHO IV | Male | < 50 | Yes | Yes | IDH WT | Non-codel |
| CGGA_287 | 571 | Dead | Mesenchymal | Primary | GBM | WHO IV | Female | < 50 | Yes | Yes | IDH WT | NA |
| CGGA_249 | 312 | Dead | Mesenchymal | Primary | LGG | WHO III | Male | < 50 | Yes | Yes | IDH WT | NA |
| CGGA_802 | 681 | Dead | Mesenchymal | Primary | GBM | WHO IV | Male | < 50 | Yes | Yes | IDH WT | Non-codel |
| CGGA_661 | NA | Dead | Mesenchymal | Primary | LGG | WHO III | Male | < 50 | Yes | Yes | IDH WT | Non-codel |
| CGGA_329 | 419 | Dead | Mesenchymal | Primary | LGG | WHO III | Male | < 50 | Yes | Yes | IDH WT | NA |
| CGGA_371 | 811 | Dead | Mesenchymal | Primary | GBM | WHO IV | Male | < 50 | Yes | Yes | IDH WT | NA |
| CGGA_1006 | 254 | Dead | Mesenchymal | Primary | LGG | WHO III | Male | < 50 | Yes | Yes | IDH WT | Non-codel |
| CGGA_513 | 1374 | Dead | Neural | Primary | LGG | WHO III | Female | < 50 | Yes | Yes | IDH WT | NA |
| CGGA_680 | 2373 | Dead | Proneural | Primary | GBM | WHO IV | Male | < 50 | Yes | Yes | IDH WT | Non-codel |
| CGGA_309 | 139 | Dead | Mesenchymal | NA | NA | NA | Male | < 50 | Yes | Yes | IDH WT | NA |
| CGGA_330 | 1121 | Dead | Mesenchymal | Primary | LGG | WHO III | Male | < 50 | Yes | Yes | IDH WT | Non-codel |
| CGGA_346 | 104 | Dead | Mesenchymal | Primary | GBM | WHO IV | Male | < 50 | Yes | Yes | IDH WT | NA |
| CGGA_693 | 1195 | Dead | Mesenchymal | Primary | GBM | WHO IV | Female | < 50 | Yes | Yes | IDH WT | NA |
| CGGA_604 | 381 | Dead | Mesenchymal | Primary | GBM | WHO IV | Male | < 50 | Yes | Yes | IDH WT | Non-codel |
| CGGA_612 | 2023 | Dead | Proneural | Primary | GBM | WHO IV | Male | < 50 | Yes | Yes | IDH WT | NA |
| CGGA_564 | 679 | Dead | Neural | Primary | LGG | WHO III | Male | < 50 | Yes | Yes | IDH WT | Non-codel |
| CGGA_195 | 486 | Dead | Mesenchymal | Primary | GBM | WHO IV | Male | < 50 | Yes | Yes | IDH WT | NA |
| CGGA_342 | 500 | Dead | Mesenchymal | Primary | GBM | WHO IV | Female | < 50 | Yes | Yes | IDH WT | NA |
| CGGA_126 | 1177 | Dead | Mesenchymal | Primary | GBM | WHO IV | Female | 50-59 | Yes | Yes | IDH WT | NA |
| CGGA_156 | 179 | Dead | Classical | Primary | GBM | WHO IV | Male | 50-59 | Yes | Yes | IDH WT | NA |
| CGGA_719 | 101 | Dead | Neural | Secondary | sGBM | WHO IV | Male | 50-59 | Yes | Yes | IDH WT | NA |
| CGGA_324 | 336 | Dead | Mesenchymal | Primary | GBM | WHO IV | Male | 50-59 | Yes | Yes | IDH WT | NA |
| CGGA_124 | 414 | Dead | Mesenchymal | Primary | GBM | WHO IV | Male | 50-59 | Yes | Yes | IDH WT | NA |
| CGGA_684 | 714 | Dead | Mesenchymal | Primary | GBM | WHO IV | Male | 50-59 | Yes | Yes | IDH WT | NA |
| CGGA_240 | 386 | Dead | Classical | Primary | GBM | WHO IV | Male | 50-59 | Yes | Yes | IDH WT | NA |
| CGGA_366 | 255 | Dead | Mesenchymal | Primary | GBM | WHO IV | Male | 50-59 | Yes | Yes | IDH WT | NA |
| CGGA_575 | 551 | Dead | Mesenchymal | Primary | GBM | WHO IV | Male | 50-59 | Yes | Yes | IDH WT | NA |
| CGGA_308 | 823 | Dead | Neural | Primary | GBM | WHO IV | Female | 50-59 | Yes | Yes | IDH WT | NA |
| CGGA_658 | 372 | Dead | Mesenchymal | Primary | GBM | WHO IV | Male | 50-59 | Yes | Yes | IDH WT | Non-codel |
| CGGA_831 | 546 | Dead | Mesenchymal | Primary | GBM | WHO IV | Female | 50-59 | Yes | Yes | IDH WT | Non-codel |
| CGGA_643 | 331 | Dead | Proneural | Primary | LGG | WHO III | Male | 50-59 | Yes | Yes | IDH WT | Non-codel |
| CGGA_633 | 1812 | Dead | Proneural | Primary | LGG | WHO II | Female | 50-59 | Yes | Yes | IDH WT | Non-codel |
| CGGA_678 | 386 | Dead | Classical | Recurrent | rLGG | WHO III | Male | 50-59 | Yes | Yes | IDH WT | Non-codel |
| CGGA_172 | 462 | Dead | Mesenchymal | Primary | GBM | WHO IV | Female | 50-59 | Yes | Yes | IDH WT | NA |
| CGGA_375 | 657 | Dead | Mesenchymal | Primary | GBM | WHO IV | Male | 50-59 | Yes | Yes | IDH WT | NA |
| CGGA_837 | 432 | Dead | Classical | Primary | GBM | WHO IV | Male | 50-59 | Yes | Yes | IDH WT | Non-codel |
| CGGA_1026 | 1570 | Dead | Classical | Primary | GBM | WHO IV | Male | 50-59 | Yes | Yes | IDH WT | Non-codel |
| CGGA_483 | 277 | Dead | Mesenchymal | Primary | GBM | WHO IV | Female | 50-59 | Yes | Yes | IDH WT | Non-codel |
| CGGA_676 | 376 | Dead | Mesenchymal | Primary | GBM | WHO IV | Male | 60-69 | Yes | Yes | IDH WT | Non-codel |
| CGGA_596 | 1618 | Dead | Mesenchymal | Primary | LGG | WHO III | Male | 60-69 | Yes | Yes | IDH WT | NA |
| CGGA_1035 | 567 | Dead | Mesenchymal | Primary | GBM | WHO IV | Female | 60-69 | Yes | Yes | IDH WT | Non-codel |
| CGGA_451 | 795 | Dead | Mesenchymal | Primary | GBM | WHO IV | Male | 60-69 | Yes | Yes | IDH WT | NA |
| CGGA_258 | 663 | Dead | Mesenchymal | Primary | LGG | WHO II | Female | 60-69 | Yes | Yes | IDH WT | NA |
| CGGA_578 | 362 | Dead | Proneural | Primary | LGG | WHO III | Female | 60-69 | Yes | Yes | IDH WT | Non-codel |
| CGGA_1078 | 315 | Dead | Mesenchymal | Recurrent | rGBM | WHO IV | Female | 60-69 | Yes | Yes | IDH WT | Non-codel |
| CGGA_606 | 325 | Dead | Mesenchymal | Primary | GBM | WHO IV | Male | 60-69 | Yes | Yes | IDH WT | NA |
| CGGA_1024 | 3074 | Dead | Mesenchymal | Primary | GBM | WHO IV | Male | 60-69 | Yes | Yes | IDH WT | Non-codel |
| CGGA_205 | 292 | Dead | Mesenchymal | Primary | GBM | WHO IV | Male | 60-69 | Yes | Yes | IDH WT | NA |
| CGGA_210 | 300 | Dead | Mesenchymal | Primary | GBM | WHO IV | Female | 60-69 | Yes | Yes | IDH WT | NA |
| CGGA_292 | 812 | Dead | Proneural | Primary | GBM | WHO IV | Male | < 50 | NA | Yes | IDH WT | NA |
| CGGA_318 | 222 | Dead | Mesenchymal | Secondary | sGBM | WHO IV | Female | < 50 | NA | Yes | IDH WT | Non-codel |
| CGGA_321 | 1681 | Alive | Neural | Primary | LGG | WHO II | Male | < 50 | No | NA | IDH WT | NA |
| CGGA_673 | 150 | Alive | Neural | Primary | LGG | WHO III | Male | 50-59 | No | NA | IDH WT | NA |
| CGGA_784 | 587 | Alive | Neural | Primary | LGG | WHO II | Male | < 50 | Yes | NA | IDH WT | NA |
| CGGA_542 | 189 | Alive | Neural | Primary | LGG | WHO II | Male | < 50 | NA | NA | IDH WT | NA |
| CGGA_869 | 537 | Alive | Mesenchymal | Recurrent | rGBM | WHO IV | Female | < 50 | NA | NA | IDH WT | Non-codel |
| CGGA_255 | 591 | Dead | Mesenchymal | Primary | GBM | WHO IV | Female | < 50 | NA | NA | IDH WT | NA |
| CGGA_380 | 165 | Dead | Proneural | Primary | GBM | WHO IV | Male | 50-59 | NA | NA | IDH WT | NA |
| CGGA_902 | 193 | Dead | Mesenchymal | Primary | GBM | WHO IV | Male | 50-59 | NA | NA | IDH WT | Non-codel |
| CGGA_907 | 177 | Dead | Mesenchymal | Primary | GBM | WHO IV | Male | 60-69 | NA | NA | IDH WT | NA |
| CGGA_404 | NA | NA | Mesenchymal | Primary | GBM | WHO IV | Male | 60-69 | NA | NA | IDH WT | NA |
