# Supplementary material for: Investigation of Genetic Determinants of Glioma Immune Phenotype by Integrative Immunogenomic Scale Analysis
Source: Front Immunol. 2021 Jun 16;12:557994. doi: 10.3389/fimmu.2021.557994 (PMC8242587; doi:10.3389/fimmu.2021.557994)
Supplement: Supplementary file 5 [file Table_4.docx]

**Supplementary Online File 4. Clinical data for CGGA RNA-seq samples**

| CGGA_ID | OS (day) | Survival status | PRS type | Histology | Grade | Gender | Age | Radioation | Pharmaceutical therapy | IDH mutation | 1p19q codeletion status |
| --- | --- | --- | --- | --- | --- | --- | --- | --- | --- | --- | --- |
| CGGA_1052 | 2964 | Alive | Primary | LGG | WHO II | Male | < 50 | NA | NA | IDH1 Mutant | Codel |
| CGGA_1643 | 170 | Alive | Primary | GBM | WHO IV | Female | 50-59 | NA | NA | IDH WT | Non-codel |
| CGGA_1972 | 915 | Alive | Recurrent | rGBM | WHO IV | Male | < 50 | NA | NA | IDH WT | Non-codel |
| CGGA_1994 | 862 | Alive | Recurrent | rLGG | WHO III | Female | < 50 | NA | NA | IDH1 Mutant | Non-codel |
| CGGA_2106 | 501 | Alive | Recurrent | rGBM | WHO IV | Female | < 50 | NA | NA | IDH1 Mutant | Codel |
| CGGA_869 | 537 | Alive | Recurrent | rGBM | WHO IV | Female | < 50 | NA | NA | IDH WT | Non-codel |
| CGGA_P11 | 433 | Alive | Primary | LGG | WHO II | Female | < 50 | NA | NA | IDH1 Mutant | Non-codel |
| CGGA_P16 | 447 | Alive | Primary | GBM | WHO IV | Male | < 50 | NA | NA | IDH1 Mutant | Codel |
| CGGA_P286 | 658 | Alive | Recurrent | rLGG | WHO II | Female | 50-59 | NA | NA | IDH1 Mutant | Codel |
| CGGA_P311 | 884 | Alive | Recurrent | rLGG | WHO II | Male | < 50 | NA | NA | IDH1 Mutant | Non-codel |
| CGGA_P335 | 840 | Alive | Recurrent | rGBM | WHO IV | Male | < 50 | NA | NA | IDH1 Mutant | Codel |
| CGGA_P346 | 522 | Alive | Recurrent | rLGG | WHO II | Male | < 50 | NA | NA | IDH1 Mutant | Non-codel |
| CGGA_P385 | 754 | Alive | Recurrent | rGBM | WHO IV | Male | < 50 | NA | NA | IDH WT | Non-codel |
| CGGA_P399 | 732 | Alive | Recurrent | rLGG | WHO II | Female | < 50 | NA | NA | IDH1 Mutant | Codel |
| CGGA_P416 | 709 | Alive | Recurrent | rLGG | WHO III | Male | < 50 | NA | NA | IDH1 Mutant | Codel |
| CGGA_P422 | 731 | Alive | Primary | LGG | WHO II | Female | < 50 | NA | NA | IDH1 Mutant | Codel |
| CGGA_P438 | 662 | Alive | Recurrent | rLGG | WHO III | Male | < 50 | NA | NA | IDH1 Mutant | Codel |
| CGGA_P461 | 626 | Alive | Recurrent | rLGG | WHO III | Male | < 50 | NA | NA | IDH1 Mutant | Non-codel |
| CGGA_P468 | 611 | Alive | Primary | LGG | WHO III | Female | < 50 | NA | NA | IDH1 Mutant | Codel |
| CGGA_P492 | 575 | Alive | Recurrent | rLGG | WHO II | Female | < 50 | NA | NA | IDH1 Mutant | Codel |
| CGGA_P501 | 563 | Alive | Recurrent | rLGG | WHO II | Male | < 50 | NA | NA | IDH1 Mutant | Non-codel |
| CGGA_P505 | 561 | Alive | Recurrent | rLGG | WHO II | Male | < 50 | NA | NA | IDH1 Mutant | Non-codel |
| CGGA_P508 | 555 | Alive | Recurrent | rLGG | WHO III | Male | < 50 | NA | NA | IDH1 Mutant | Non-codel |
| CGGA_P510 | 549 | Alive | Recurrent | rLGG | WHO III | Male | < 50 | NA | NA | IDH1 Mutant | Non-codel |
| CGGA_P512 | 535 | Alive | Recurrent | rGBM | WHO IV | Male | < 50 | NA | NA | IDH WT | Non-codel |
| CGGA_P520 | 521 | Alive | Recurrent | rLGG | WHO III | Female | < 50 | NA | NA | IDH1 Mutant | Codel |
| CGGA_P585 | 431 | Alive | Recurrent | rGBM | WHO IV | Male | 50-59 | NA | NA | IDH WT | Non-codel |
| CGGA_P596 | 411 | Alive | Recurrent | rGBM | WHO IV | Female | 60-69 | NA | NA | IDH1 Mutant | Codel |
| CGGA_P619 | 354 | Alive | Recurrent | rGBM | WHO IV | Female | 60-69 | NA | NA | IDH WT | Non-codel |
| CGGA_1032 | 643 | Dead | Primary | LGG | WHO II | Female | < 50 | NA | NA | IDH WT | NA |
| CGGA_1051 | 838 | Dead | Primary | LGG | WHO II | Male | < 50 | NA | NA | IDH WT | Codel |
| CGGA_1079 | 471 | Dead | Primary | LGG | WHO III | Male | < 50 | NA | NA | IDH WT | Non-codel |
| CGGA_1109 | 1348 | Dead | Primary | GBM | WHO IV | Male | < 50 | NA | NA | IDH WT | Non-codel |
| CGGA_1141 | 432 | Dead | Primary | LGG | WHO III | Male | 60-69 | NA | NA | IDH WT | Non-codel |
| CGGA_1154 | 369 | Dead | Recurrent | rLGG | WHO III | Female | < 50 | NA | NA | IDH1 Mutant | Non-codel |
| CGGA_1160 | 426 | Dead | Primary | LGG | WHO III | Female | < 50 | NA | NA | IDH1 Mutant | Non-codel |
| CGGA_1529 | 583 | Dead | Primary | GBM | WHO IV | Male | 60-69 | NA | NA | IDH WT | Non-codel |
| CGGA_1697 | 89 | Dead | Recurrent | rGBM | WHO IV | Female | < 50 | NA | NA | IDH WT | Non-codel |
| CGGA_1807 | 247 | Dead | Primary | GBM | WHO IV | Female | 60-69 | NA | NA | IDH WT | Non-codel |
| CGGA_1812 | 780 | Dead | Primary | GBM | WHO IV | Male | 60-69 | NA | NA | IDH WT | Non-codel |
| CGGA_1985 | 380 | Dead | Recurrent | rGBM | WHO IV | Male | < 50 | NA | NA | IDH1 Mutant | Non-codel |
| CGGA_2008 | 44 | Dead | Recurrent | rGBM | WHO IV | Male | 50-59 | NA | NA | IDH WT | Non-codel |
| CGGA_2038 | NA | Dead | Recurrent | rGBM | WHO IV | Male | < 50 | NA | NA | IDH1 Mutant | Non-codel |
| CGGA_337 | 1008 | Dead | Primary | LGG | WHO III | Female | 50-59 | NA | NA | IDH1 Mutant | Non-codel |
| CGGA_624 | 122 | Dead | Recurrent | rGBM | WHO IV | Female | 50-59 | NA | NA | IDH WT | Non-codel |
| CGGA_876 | 159 | Dead | Primary | GBM | WHO IV | Female | 60-69 | NA | NA | IDH WT | Non-codel |
| CGGA_902 | 193 | Dead | Primary | GBM | WHO IV | Male | 50-59 | NA | NA | IDH WT | Non-codel |
| CGGA_D06 | NA | Dead | Recurrent | rLGG | WHO III | Female | < 50 | NA | NA | IDH WT | Codel |
| CGGA_P100 | 268 | Dead | Primary | GBM | WHO IV | Male | 60-69 | NA | NA | IDH WT | Non-codel |
| CGGA_P181 | 238 | Dead | Primary | LGG | WHO III | Female | < 50 | NA | NA | IDH WT | Non-codel |
| CGGA_P310 | 224 | Dead | Recurrent | rLGG | WHO III | Female | < 50 | NA | NA | NA | Codel |
| CGGA_P337 | 689 | Dead | Recurrent | rLGG | WHO III | Female | 60-69 | NA | NA | IDH1 Mutant | Codel |
| CGGA_P356 | 569 | Dead | Recurrent | rLGG | WHO II | Female | < 50 | NA | NA | IDH1 Mutant | Non-codel |
| CGGA_P388 | 293 | Dead | Recurrent | rLGG | WHO III | Female | < 50 | NA | NA | IDH1 Mutant | Non-codel |
| CGGA_P401 | 788 | Dead | Recurrent | rGBM | WHO IV | Male | < 50 | NA | NA | IDH1 Mutant | Non-codel |
| CGGA_P415 | 195 | Dead | Recurrent | rGBM | WHO IV | Female | 50-59 | NA | NA | IDH WT | Non-codel |
| CGGA_P421 | 308 | Dead | Recurrent | rLGG | WHO III | Male | < 50 | NA | NA | IDH1 Mutant | Non-codel |
| CGGA_P483 | 299 | Dead | Recurrent | rLGG | WHO III | Male | < 50 | NA | NA | IDH WT | Non-codel |
| CGGA_P594 | 84 | Dead | Recurrent | rLGG | WHO III | Male | < 50 | NA | NA | IDH WT | Non-codel |
| CGGA_P625 | 161 | Dead | Recurrent | rGBM | WHO IV | Female | < 50 | NA | NA | IDH1 Mutant | Non-codel |
| CGGA_1063 | NA | NA | Primary | LGG | WHO II | Male | < 50 | NA | NA | IDH1 Mutant | NA |
| CGGA_1069 | NA | NA | Recurrent | rLGG | WHO II | Male | < 50 | NA | NA | IDH1 Mutant | Non-codel |
| CGGA_1082 | NA | NA | Primary | LGG | WHO II | Female | 50-59 | NA | NA | IDH1 Mutant | Non-codel |
| CGGA_1498 | NA | NA | Primary | GBM | WHO IV | Male | 60-69 | NA | NA | IDH WT | NA |
| CGGA_1531 | NA | NA | Primary | LGG | WHO III | Male | < 50 | NA | NA | IDH1 Mutant | Non-codel |
| CGGA_1654 | NA | NA | Primary | GBM | WHO IV | Male | 60-69 | NA | NA | IDH WT | Non-codel |
| CGGA_1672 | NA | NA | Primary | LGG | WHO III | Female | < 50 | NA | NA | NA | Non-codel |
| CGGA_1677 | NA | NA | Primary | LGG | WHO II | Female | < 50 | NA | NA | NA | Non-codel |
| CGGA_1684 | NA | NA | Primary | GBM | WHO IV | Male | 50-59 | NA | NA | IDH WT | Non-codel |
| CGGA_1877 | NA | NA | Recurrent | rLGG | WHO III | Male | < 50 | NA | NA | IDH1 Mutant | Codel |
| CGGA_2039 | NA | NA | Recurrent | rGBM | WHO IV | Male | < 50 | NA | NA | IDH WT | Non-codel |
| CGGA_D14 | NA | NA | Recurrent | rLGG | WHO III | Male | < 50 | NA | NA | IDH1 Mutant | Non-codel |
| CGGA_D18 | NA | NA | Primary | LGG | WHO III | Male | < 50 | NA | NA | IDH WT | Non-codel |
| CGGA_D39 | NA | NA | Recurrent | rLGG | WHO II | Female | < 50 | NA | NA | IDH1 Mutant | Non-codel |
| CGGA_D49 | NA | NA | Recurrent | NA | NA | Female | < 50 | NA | NA | IDH1 Mutant | Codel |
| CGGA_D53 | NA | NA | Recurrent | rGBM | WHO IV | Male | < 50 | NA | NA | IDH WT | Non-codel |
| CGGA_J100 | NA | NA | Recurrent | rGBM | WHO IV | Male | 50-59 | NA | NA | IDH1 Mutant | Non-codel |
| CGGA_P107 | NA | NA | Primary | LGG | WHO II | Male | < 50 | NA | NA | IDH1 Mutant | Non-codel |
| CGGA_P154 | NA | NA | Primary | GBM | WHO IV | Male | 50-59 | NA | NA | IDH1 Mutant | NA |
| CGGA_P499 | NA | NA | Recurrent | rGBM | WHO IV | Female | 60-69 | NA | NA | IDH WT | Non-codel |
| CGGA_P623 | NA | NA | Primary | LGG | WHO III | Male | 50-59 | NA | NA | IDH1 Mutant | Non-codel |
| CGGA_362 | 1680 | Alive | Recurrent | rLGG | WHO II | Female | < 50 | No | NA | IDH1 Mutant | Codel |
| CGGA_862 | 1046 | Alive | Recurrent | rLGG | WHO II | Male | < 50 | No | NA | IDH1 Mutant | Non-codel |
| CGGA_883 | 1016 | Alive | Recurrent | rLGG | WHO II | Female | < 50 | No | NA | IDH1 Mutant | Non-codel |
| CGGA_887 | 1011 | Alive | Recurrent | rLGG | WHO III | Male | < 50 | No | NA | IDH WT | Non-codel |
| CGGA_1325 | NA | NA | Recurrent | rGBM | WHO IV | Male | < 50 | No | NA | IDH WT | NA |
| CGGA_171 | NA | NA | Primary | LGG | WHO II | Female | < 50 | No | NA | IDH1 Mutant | Codel |
| CGGA_538 | NA | NA | Primary | LGG | WHO II | Female | < 50 | No | NA | IDH1 Mutant | Codel |
| CGGA_732 | NA | NA | Primary | LGG | WHO III | Female | < 50 | No | NA | IDH WT | Non-codel |
| CGGA_1383 | 884 | Alive | Recurrent | rLGG | WHO III | Female | < 50 | Yes | NA | IDH1 Mutant | Codel |
| CGGA_1443 | 2284 | Alive | Recurrent | rLGG | WHO II | Male | < 50 | Yes | NA | IDH WT | Non-codel |
| CGGA_835 | 517 | Alive | Primary | LGG | WHO II | Male | < 50 | Yes | NA | IDH1 Mutant | Codel |
| CGGA_856 | 1297 | Alive | Primary | LGG | WHO II | Male | 50-59 | Yes | NA | IDH1 Mutant | Codel |
| CGGA_892 | 448 | Alive | Primary | LGG | WHO II | Male | < 50 | Yes | NA | IDH1 Mutant | Codel |
| CGGA_P306 | 895 | Alive | Recurrent | rLGG | WHO II | Male | < 50 | Yes | NA | IDH1 Mutant | Codel |
| CGGA_P319 | 868 | Alive | Recurrent | rLGG | WHO III | Male | < 50 | Yes | NA | IDH1 Mutant | Non-codel |
| CGGA_P338 | 838 | Alive | Recurrent | rLGG | WHO II | Male | 50-59 | Yes | NA | IDH WT | Non-codel |
| CGGA_P437 | 662 | Alive | Primary | LGG | WHO III | Female | < 50 | Yes | NA | IDH1 Mutant | Codel |
| CGGA_1374 | 141 | Dead | Recurrent | rLGG | WHO III | Male | < 50 | Yes | NA | IDH1 Mutant | Non-codel |
| CGGA_1375 | 92 | Dead | Secondary | sGBM | WHO IV | Female | 50-59 | Yes | NA | IDH1 Mutant | Non-codel |
| CGGA_2013 | 139 | Dead | Recurrent | rLGG | WHO II | Male | < 50 | Yes | NA | IDH1 Mutant | Codel |
| CGGA_2024 | 328 | Dead | Recurrent | rGBM | WHO IV | Female | < 50 | Yes | NA | IDH WT | Non-codel |
| CGGA_898 | 1663 | Dead | Primary | LGG | WHO II | Male | < 50 | Yes | NA | IDH1 Mutant | Non-codel |
| CGGA_908 | 2436 | Dead | Primary | LGG | WHO II | Male | < 50 | Yes | NA | IDH1 Mutant | Non-codel |
| CGGA_P114 | NA | Dead | Primary | LGG | WHO II | Male | < 50 | Yes | NA | IDH1 Mutant | Codel |
| CGGA_P116 | 305 | Dead | Primary | GBM | WHO IV | Male | 50-59 | Yes | NA | IDH WT | NA |
| CGGA_P328 | 580 | Dead | Recurrent | rLGG | WHO III | Female | 50-59 | Yes | NA | IDH1 Mutant | Non-codel |
| CGGA_P364 | 495 | Dead | Recurrent | rLGG | WHO III | Female | < 50 | Yes | NA | IDH1 Mutant | Non-codel |
| CGGA_909 | 3582 | Alive | Primary | LGG | WHO II | Female | < 50 | NA | No | IDH1 Mutant | Non-codel |
| CGGA_318 | 222 | Dead | Secondary | sGBM | WHO IV | Female | < 50 | NA | No | IDH WT | Non-codel |
| CGGA_773 | 443 | Dead | Secondary | sGBM | WHO IV | Female | 50-59 | NA | No | IDH1 Mutant | Non-codel |
| CGGA_1191 | 2695 | Alive | Primary | LGG | WHO III | Male | 50-59 | No | No | IDH1 Mutant | Codel |
| CGGA_1211 | 2654 | Alive | Primary | LGG | WHO II | Male | < 50 | No | No | IDH1 Mutant | Codel |
| CGGA_1238 | 2611 | Alive | Primary | LGG | WHO II | Female | < 50 | No | No | IDH WT | Non-codel |
| CGGA_1455 | 2274 | Alive | Primary | LGG | WHO II | Male | < 50 | No | No | IDH1 Mutant | NA |
| CGGA_1488 | 2206 | Alive | Primary | LGG | WHO II | Male | < 50 | No | No | IDH1 Mutant | Non-codel |
| CGGA_1502 | 2185 | Alive | Primary | LGG | WHO II | Female | < 50 | No | No | IDH1 Mutant | Codel |
| CGGA_1565 | 1417 | Alive | Primary | LGG | WHO III | Female | < 50 | No | No | IDH1 Mutant | Non-codel |
| CGGA_1568 | 345 | Alive | Primary | LGG | WHO III | Female | < 50 | No | No | NA | Non-codel |
| CGGA_1569 | 1406 | Alive | Primary | LGG | WHO III | Female | < 50 | No | No | IDH WT | Codel |
| CGGA_1579 | 2018 | Alive | Primary | LGG | WHO II | Male | < 50 | No | No | IDH WT | Non-codel |
| CGGA_1588 | 2003 | Alive | Primary | LGG | WHO III | Female | < 50 | No | No | IDH WT | Non-codel |
| CGGA_1621 | 1931 | Alive | Primary | LGG | WHO II | Female | < 50 | No | No | IDH1 Mutant | Non-codel |
| CGGA_1630 | 1912 | Alive | Primary | LGG | WHO II | Male | < 50 | No | No | NA | Codel |
| CGGA_1657 | 1835 | Alive | Primary | LGG | WHO III | Female | < 50 | No | No | NA | Non-codel |
| CGGA_1673 | 1781 | Alive | Primary | LGG | WHO II | Female | < 50 | No | No | IDH1 Mutant | Non-codel |
| CGGA_1715 | 1653 | Alive | Primary | LGG | WHO II | Female | < 50 | No | No | IDH WT | Non-codel |
| CGGA_1829 | 801 | Alive | Primary | LGG | WHO II | Male | < 50 | No | No | IDH WT | Non-codel |
| CGGA_1880 | 1168 | Alive | Recurrent | rLGG | WHO III | Female | < 50 | No | No | IDH1 Mutant | Non-codel |
| CGGA_1882 | 1155 | Alive | Primary | LGG | WHO III | Male | < 50 | No | No | IDH1 Mutant | Codel |
| CGGA_1902 | 1103 | Alive | Primary | LGG | WHO II | Male | < 50 | No | No | IDH1 Mutant | Non-codel |
| CGGA_1916 | NA | Alive | Primary | GBM | WHO IV | Female | < 50 | No | No | IDH1 Mutant | Non-codel |
| CGGA_2129 | 439 | Alive | Recurrent | rLGG | WHO III | Male | < 50 | No | No | IDH1 Mutant | Non-codel |
| CGGA_276 | 529 | Alive | Primary | LGG | WHO II | Male | < 50 | No | No | IDH1 Mutant | Non-codel |
| CGGA_323 | 4338 | Alive | Primary | LGG | WHO II | Female | < 50 | No | No | IDH WT | Non-codel |
| CGGA_724 | 3817 | Alive | Recurrent | rLGG | WHO II | Male | < 50 | No | No | IDH1 Mutant | Non-codel |
| CGGA_850 | 564 | Alive | Primary | GBM | WHO IV | Female | 50-59 | No | No | IDH WT | Non-codel |
| CGGA_D04 | 3359 | Alive | Primary | LGG | WHO II | Male | < 50 | No | No | IDH1 Mutant | Non-codel |
| CGGA_D40 | 3219 | Alive | Recurrent | rLGG | WHO II | Male | < 50 | No | No | IDH1 Mutant | Non-codel |
| CGGA_J024 | 2801 | Alive | Recurrent | rLGG | WHO II | Female | < 50 | No | No | IDH1 Mutant | Non-codel |
| CGGA_P13 | 1569 | Alive | Primary | LGG | WHO II | Female | < 50 | No | No | IDH1 Mutant | Codel |
| CGGA_P153 | 1122 | Alive | Primary | LGG | WHO II | Male | < 50 | No | No | IDH1 Mutant | NA |
| CGGA_P156 | 1126 | Alive | Primary | LGG | WHO II | Male | < 50 | No | No | IDH1 Mutant | Codel |
| CGGA_P314 | 875 | Alive | Recurrent | rLGG | WHO III | Female | < 50 | No | No | IDH1 Mutant | Codel |
| CGGA_P326 | 854 | Alive | Recurrent | rLGG | WHO III | Female | < 50 | No | No | IDH1 Mutant | Non-codel |
| CGGA_P609 | 380 | Alive | Primary | GBM | WHO IV | Female | < 50 | No | No | IDH WT | Non-codel |
| CGGA_P610 | 373 | Alive | Recurrent | rGBM | WHO IV | Male | 60-69 | No | No | IDH WT | NA |
| CGGA_P93 | 1310 | Alive | Primary | LGG | WHO II | Female | < 50 | No | No | IDH1 Mutant | Non-codel |
| CGGA_1068 | 68 | Dead | Secondary | sGBM | WHO IV | Male | < 50 | No | No | IDH1 Mutant | Non-codel |
| CGGA_107 | 2156 | Dead | Recurrent | rLGG | WHO III | Female | < 50 | No | No | IDH WT | Non-codel |
| CGGA_1101 | 214 | Dead | Primary | LGG | WHO II | Female | < 50 | No | No | IDH WT | NA |
| CGGA_1118 | 398 | Dead | Recurrent | rLGG | WHO III | Male | < 50 | No | No | IDH1 Mutant | Non-codel |
| CGGA_1195 | 476 | Dead | Primary | LGG | WHO III | Male | < 50 | No | No | IDH1 Mutant | Non-codel |
| CGGA_1236 | 191 | Dead | Primary | GBM | WHO IV | Female | < 50 | No | No | IDH WT | Non-codel |
| CGGA_1240 | 19 | Dead | Primary | GBM | WHO IV | Male | < 50 | No | No | IDH WT | Non-codel |
| CGGA_1258 | 114 | Dead | Primary | GBM | WHO IV | Male | < 50 | No | No | IDH WT | Non-codel |
| CGGA_1285 | 79 | Dead | Recurrent | rGBM | WHO IV | Female | 50-59 | No | No | IDH WT | Non-codel |
| CGGA_1314 | 172 | Dead | Primary | GBM | WHO IV | Female | < 50 | No | No | IDH1 Mutant | Non-codel |
| CGGA_1416 | 1933 | Dead | Primary | LGG | WHO III | Male | < 50 | No | No | IDH1 Mutant | Codel |
| CGGA_1472 | 1025 | Dead | Primary | GBM | WHO IV | Male | < 50 | No | No | NA | Non-codel |
| CGGA_1475 | 182 | Dead | Secondary | sGBM | WHO IV | Male | < 50 | No | No | IDH1 Mutant | Non-codel |
| CGGA_1476 | 299 | Dead | Primary | GBM | WHO IV | Female | 50-59 | No | No | IDH WT | Non-codel |
| CGGA_1481 | 131 | Dead | Primary | GBM | WHO IV | Male | 50-59 | No | No | IDH WT | NA |
| CGGA_1508 | 1049 | Dead | Primary | LGG | WHO III | Male | < 50 | No | No | IDH1 Mutant | Non-codel |
| CGGA_1514 | 76 | Dead | Primary | LGG | WHO II | Female | 50-59 | No | No | IDH WT | NA |
| CGGA_1534 | 27 | Dead | Primary | GBM | WHO IV | Female | 50-59 | No | No | IDH1 Mutant | Non-codel |
| CGGA_1536 | 840 | Dead | Primary | LGG | WHO II | Male | 50-59 | No | No | IDH1 Mutant | Non-codel |
| CGGA_1537 | 97 | Dead | Primary | GBM | WHO IV | Male | 70-79 | No | No | IDH WT | Non-codel |
| CGGA_1544 | 208 | Dead | Primary | LGG | WHO III | Female | < 50 | No | No | IDH1 Mutant | Non-codel |
| CGGA_1587 | 750 | Dead | Primary | LGG | WHO III | Female | < 50 | No | No | IDH1 Mutant | Non-codel |
| CGGA_1634 | 366 | Dead | Primary | GBM | WHO IV | Female | < 50 | No | No | IDH WT | Non-codel |
| CGGA_1669 | 139 | Dead | Primary | LGG | WHO III | Female | < 50 | No | No | IDH WT | Non-codel |
| CGGA_1698 | 388 | Dead | Primary | GBM | WHO IV | Female | 50-59 | No | No | IDH WT | Non-codel |
| CGGA_1740 | 363 | Dead | Primary | GBM | WHO IV | Female | 50-59 | No | No | IDH WT | Non-codel |
| CGGA_1743 | 1361 | Dead | Primary | LGG | WHO III | Female | < 50 | No | No | IDH1 Mutant | Non-codel |
| CGGA_1767 | 86 | Dead | Primary | GBM | WHO IV | Male | 60-69 | No | No | IDH WT | NA |
| CGGA_1826 | 44 | Dead | Primary | GBM | WHO IV | Female | 70-79 | No | No | IDH WT | Non-codel |
| CGGA_1865 | 89 | Dead | Recurrent | rGBM | WHO IV | Female | < 50 | No | No | IDH WT | Non-codel |
| CGGA_267 | 1374 | Dead | Primary | LGG | WHO II | Male | < 50 | No | No | IDH1 Mutant | Non-codel |
| CGGA_358 | 122 | Dead | Recurrent | rLGG | WHO III | Female | < 50 | No | No | IDH1 Mutant | Non-codel |
| CGGA_406 | 90 | Dead | Recurrent | rLGG | WHO III | Female | < 50 | No | No | IDH1 Mutant | Non-codel |
| CGGA_457 | 75 | Dead | Recurrent | rLGG | WHO III | Male | < 50 | No | No | IDH1 Mutant | Non-codel |
| CGGA_495 | 295 | Dead | NA | NA | NA | Male | 50-59 | No | No | IDH WT | NA |
| CGGA_625 | 2465 | Dead | Recurrent | rLGG | WHO III | Female | < 50 | No | No | IDH1 Mutant | Codel |
| CGGA_662 | 284 | Dead | Primary | LGG | WHO II | Female | 50-59 | No | No | IDH1 Mutant | Codel |
| CGGA_681 | 110 | Dead | NA | NA | NA | Male | < 50 | No | No | IDH WT | NA |
| CGGA_719 | 101 | Dead | Secondary | sGBM | WHO IV | Male | 50-59 | No | No | IDH1 Mutant | NA |
| CGGA_861 | 893 | Dead | Primary | LGG | WHO III | Male | < 50 | No | No | IDH WT | Non-codel |
| CGGA_867 | 367 | Dead | Recurrent | rLGG | WHO III | Female | < 50 | No | No | IDH1 Mutant | Non-codel |
| CGGA_D20 | 2367 | Dead | Primary | LGG | WHO II | Female | < 50 | No | No | IDH1 Mutant | Non-codel |
| CGGA_D33 | 116 | Dead | Recurrent | rLGG | WHO III | Male | < 50 | No | No | IDH1 Mutant | Non-codel |
| CGGA_D34 | 147 | Dead | Secondary | sGBM | WHO IV | Male | < 50 | No | No | IDH1 Mutant | Non-codel |
| CGGA_D36 | 34 | Dead | Recurrent | rGBM | WHO IV | Male | 50-59 | No | No | IDH WT | Non-codel |
| CGGA_D50 | 782 | Dead | Recurrent | rLGG | WHO II | Male | < 50 | No | No | IDH1 Mutant | Codel |
| CGGA_D58 | 181 | Dead | Recurrent | rLGG | WHO III | Male | < 50 | No | No | IDH WT | Codel |
| CGGA_J042 | 1409 | Dead | Recurrent | rLGG | WHO II | Male | < 50 | No | No | IDH1 Mutant | Non-codel |
| CGGA_P159 | 211 | Dead | Primary | LGG | WHO III | Male | 50-59 | No | No | IDH WT | Non-codel |
| CGGA_P199 | 203 | Dead | Recurrent | rGBM | WHO IV | Male | 60-69 | No | No | IDH WT | Non-codel |
| CGGA_P28 | 107 | Dead | Primary | GBM | WHO IV | Male | 60-69 | No | No | IDH WT | Non-codel |
| CGGA_P568 | 218 | Dead | Recurrent | rLGG | WHO III | Female | < 50 | No | No | IDH1 Mutant | Codel |
| CGGA_P604 | 262 | Dead | Recurrent | rLGG | WHO III | Female | < 50 | No | No | IDH WT | Non-codel |
| CGGA_P615 | NA | NA | Recurrent | rLGG | WHO II | Male | < 50 | No | No | IDH1 Mutant | Codel |
| CGGA_1005 | 3426 | Alive | Primary | LGG | WHO II | Female | < 50 | Yes | No | IDH1 Mutant | Non-codel |
| CGGA_1037 | 780 | Alive | Primary | LGG | WHO II | Male | < 50 | Yes | No | NA | Codel |
| CGGA_1050 | 3040 | Alive | Primary | LGG | WHO II | Male | < 50 | Yes | No | IDH1 Mutant | Codel |
| CGGA_1071 | 3009 | Alive | Primary | LGG | WHO II | Male | < 50 | Yes | No | IDH WT | Non-codel |
| CGGA_1156 | 2805 | Alive | Primary | LGG | WHO II | Female | < 50 | Yes | No | IDH1 Mutant | Non-codel |
| CGGA_1161 | 2791 | Alive | Primary | LGG | WHO III | Male | < 50 | Yes | No | IDH1 Mutant | Non-codel |
| CGGA_1318 | 1497 | Alive | Primary | LGG | WHO II | Male | < 50 | Yes | No | IDH1 Mutant | Non-codel |
| CGGA_1345 | 2437 | Alive | Primary | LGG | WHO II | Male | 50-59 | Yes | No | IDH1 Mutant | Codel |
| CGGA_1368 | 2409 | Alive | Primary | LGG | WHO II | Male | < 50 | Yes | No | IDH1 Mutant | NA |
| CGGA_1404 | 2353 | Alive | Primary | LGG | WHO III | Female | 50-59 | Yes | No | IDH1 Mutant | Codel |
| CGGA_1427 | 339 | Alive | Primary | LGG | WHO II | Female | < 50 | Yes | No | NA | Non-codel |
| CGGA_1454 | 2274 | Alive | Primary | LGG | WHO II | Female | < 50 | Yes | No | IDH WT | Non-codel |
| CGGA_1463 | 2252 | Alive | Primary | LGG | WHO II | Female | 60-69 | Yes | No | IDH1 Mutant | Codel |
| CGGA_1517 | 2150 | Alive | Primary | LGG | WHO II | Male | < 50 | Yes | No | IDH1 Mutant | Codel |
| CGGA_1528 | 2117 | Alive | Primary | LGG | WHO II | Female | < 50 | Yes | No | IDH1 Mutant | Codel |
| CGGA_1562 | 2045 | Alive | Primary | LGG | WHO III | Male | 50-59 | Yes | No | IDH1 Mutant | Codel |
| CGGA_1563 | 2043 | Alive | Primary | LGG | WHO II | Female | < 50 | Yes | No | IDH WT | Non-codel |
| CGGA_1608 | 1953 | Alive | Recurrent | rLGG | WHO III | Male | < 50 | Yes | No | IDH1 Mutant | Non-codel |
| CGGA_1617 | 1935 | Alive | Primary | LGG | WHO II | Female | < 50 | Yes | No | NA | Codel |
| CGGA_1623 | 1927 | Alive | Primary | LGG | WHO III | Female | < 50 | Yes | No | IDH WT | Non-codel |
| CGGA_1642 | 1885 | Alive | Primary | LGG | WHO II | Male | < 50 | Yes | No | IDH1 Mutant | Non-codel |
| CGGA_165 | 183 | Alive | Primary | LGG | WHO II | Male | < 50 | Yes | No | NA | Non-codel |
| CGGA_1653 | 1837 | Alive | Primary | LGG | WHO II | Female | < 50 | Yes | No | NA | Non-codel |
| CGGA_1655 | 1209 | Alive | Primary | LGG | WHO II | Male | < 50 | Yes | No | IDH1 Mutant | Non-codel |
| CGGA_1662 | 1493 | Alive | Primary | LGG | WHO II | Female | 50-59 | Yes | No | IDH1 Mutant | Codel |
| CGGA_1689 | 1738 | Alive | Primary | LGG | WHO II | Male | < 50 | Yes | No | IDH1 Mutant | Non-codel |
| CGGA_1704 | 1684 | Alive | Primary | LGG | WHO II | Female | < 50 | Yes | No | IDH WT | Non-codel |
| CGGA_2081 | 571 | Alive | Recurrent | rLGG | WHO III | Female | < 50 | Yes | No | IDH1 Mutant | Codel |
| CGGA_241 | 4478 | Alive | Primary | LGG | WHO II | Male | < 50 | Yes | No | IDH1 Mutant | Non-codel |
| CGGA_243 | 2977 | Alive | Primary | LGG | WHO II | Female | < 50 | Yes | No | IDH1 Mutant | Codel |
| CGGA_251 | 1299 | Alive | Primary | LGG | WHO II | Male | < 50 | Yes | No | IDH1 Mutant | Non-codel |
| CGGA_252 | 165 | Alive | Primary | LGG | WHO II | Female | < 50 | Yes | No | IDH1 Mutant | Non-codel |
| CGGA_261 | 4130 | Alive | Primary | LGG | WHO II | Male | < 50 | Yes | No | IDH1 Mutant | Non-codel |
| CGGA_269 | 2932 | Alive | Primary | LGG | WHO II | Female | < 50 | Yes | No | IDH WT | Non-codel |
| CGGA_290 | 4390 | Alive | Primary | LGG | WHO II | Female | < 50 | Yes | No | IDH1 Mutant | Codel |
| CGGA_333 | 1783 | Alive | Primary | LGG | WHO II | Male | < 50 | Yes | No | IDH1 Mutant | Non-codel |
| CGGA_369 | 4271 | Alive | Recurrent | rLGG | WHO II | Male | < 50 | Yes | No | IDH1 Mutant | Non-codel |
| CGGA_446 | 4173 | Alive | Primary | LGG | WHO II | Female | < 50 | Yes | No | IDH1 Mutant | Codel |
| CGGA_479 | 4119 | Alive | Primary | LGG | WHO II | Female | < 50 | Yes | No | IDH1 Mutant | Codel |
| CGGA_485 | 2437 | Alive | Primary | LGG | WHO II | Male | < 50 | Yes | No | IDH1 Mutant | Codel |
| CGGA_501 | 4101 | Alive | Primary | LGG | WHO II | Male | < 50 | Yes | No | IDH1 Mutant | Codel |
| CGGA_502 | 4088 | Alive | Primary | LGG | WHO II | Male | < 50 | Yes | No | IDH1 Mutant | Non-codel |
| CGGA_543 | 4042 | Alive | Primary | LGG | WHO II | Male | < 50 | Yes | No | IDH1 Mutant | Codel |
| CGGA_551 | 4034 | Alive | Primary | LGG | WHO II | Male | < 50 | Yes | No | IDH1 Mutant | Codel |
| CGGA_560 | 4025 | Alive | Primary | LGG | WHO III | Male | < 50 | Yes | No | IDH1 Mutant | Non-codel |
| CGGA_607 | 1389 | Alive | Primary | LGG | WHO II | Male | < 50 | Yes | No | IDH1 Mutant | Non-codel |
| CGGA_630 | 3971 | Alive | Recurrent | rLGG | WHO III | Male | < 50 | Yes | No | IDH1 Mutant | Codel |
| CGGA_638 | 2386 | Alive | Primary | LGG | WHO II | Male | < 50 | Yes | No | IDH1 Mutant | Codel |
| CGGA_642 | 3920 | Alive | Primary | LGG | WHO II | Male | < 50 | Yes | No | IDH1 Mutant | Non-codel |
| CGGA_655 | 3901 | Alive | Primary | LGG | WHO II | Male | < 50 | Yes | No | IDH1 Mutant | Codel |
| CGGA_663 | 3880 | Alive | Primary | LGG | WHO II | Female | < 50 | Yes | No | IDH1 Mutant | Non-codel |
| CGGA_671 | 3885 | Alive | Primary | LGG | WHO II | Female | < 50 | Yes | No | IDH1 Mutant | Codel |
| CGGA_675 | 3880 | Alive | Primary | LGG | WHO II | Female | < 50 | Yes | No | IDH1 Mutant | Codel |
| CGGA_689 | 3864 | Alive | Primary | LGG | WHO II | Female | < 50 | Yes | No | IDH1 Mutant | Codel |
| CGGA_715 | 3429 | Alive | Primary | LGG | WHO II | Male | < 50 | Yes | No | IDH1 Mutant | Codel |
| CGGA_725 | 3817 | Alive | Primary | LGG | WHO II | Male | 50-59 | Yes | No | IDH1 Mutant | Codel |
| CGGA_738 | 3802 | Alive | Primary | LGG | WHO II | Male | < 50 | Yes | No | IDH1 Mutant | Non-codel |
| CGGA_752 | 3680 | Alive | Primary | LGG | WHO II | Male | < 50 | Yes | No | IDH1 Mutant | Codel |
| CGGA_760 | 3780 | Alive | Primary | LGG | WHO II | Female | < 50 | Yes | No | IDH1 Mutant | Codel |
| CGGA_762 | 3775 | Alive | Primary | LGG | WHO II | Female | < 50 | Yes | No | IDH1 Mutant | Codel |
| CGGA_763 | 319 | Alive | Recurrent | rLGG | WHO III | Male | 50-59 | Yes | No | IDH WT | Non-codel |
| CGGA_766 | 3773 | Alive | Primary | LGG | WHO II | Male | < 50 | Yes | No | IDH1 Mutant | Codel |
| CGGA_812 | 3705 | Alive | Primary | LGG | WHO II | Male | < 50 | Yes | No | IDH1 Mutant | Codel |
| CGGA_818 | 3697 | Alive | Primary | LGG | WHO II | Male | < 50 | Yes | No | IDH1 Mutant | Non-codel |
| CGGA_834 | 3683 | Alive | Primary | LGG | WHO II | Male | < 50 | Yes | No | IDH WT | Non-codel |
| CGGA_843 | 3675 | Alive | Primary | LGG | WHO II | Male | < 50 | Yes | No | IDH1 Mutant | Codel |
| CGGA_848 | 239 | Alive | Primary | GBM | WHO IV | Female | 50-59 | Yes | No | IDH WT | Non-codel |
| CGGA_858 | 3654 | Alive | Primary | LGG | WHO II | Male | < 50 | Yes | No | IDH1 Mutant | Codel |
| CGGA_864 | 3647 | Alive | Primary | LGG | WHO II | Male | < 50 | Yes | No | IDH1 Mutant | Codel |
| CGGA_871 | 3634 | Alive | Primary | LGG | WHO II | Male | < 50 | Yes | No | IDH1 Mutant | Codel |
| CGGA_882 | 1008 | Alive | Recurrent | rLGG | WHO III | Male | < 50 | Yes | No | IDH1 Mutant | Non-codel |
| CGGA_D07 | 3353 | Alive | Primary | LGG | WHO II | Male | < 50 | Yes | No | IDH1 Mutant | Codel |
| CGGA_D15 | 3331 | Alive | Primary | LGG | WHO II | Female | < 50 | Yes | No | IDH1 Mutant | Codel |
| CGGA_D29 | 3313 | Alive | Primary | LGG | WHO II | Male | < 50 | Yes | No | IDH1 Mutant | Codel |
| CGGA_J030 | 2775 | Alive | Primary | LGG | WHO II | Male | < 50 | Yes | No | IDH1 Mutant | Codel |
| CGGA_J130 | 2806 | Alive | Recurrent | rLGG | WHO II | Male | < 50 | Yes | No | IDH WT | Non-codel |
| CGGA_P103 | 1283 | Alive | Primary | LGG | WHO II | Male | < 50 | Yes | No | IDH1 Mutant | Codel |
| CGGA_P111 | 1270 | Alive | Primary | LGG | WHO II | Female | < 50 | Yes | No | IDH1 Mutant | Codel |
| CGGA_P113 | 1340 | Alive | Primary | LGG | WHO II | Male | < 50 | Yes | No | IDH WT | Non-codel |
| CGGA_P115 | 1262 | Alive | Primary | LGG | WHO II | Male | < 50 | Yes | No | IDH WT | NA |
| CGGA_P121 | 1244 | Alive | Primary | LGG | WHO II | Male | < 50 | Yes | No | IDH WT | Non-codel |
| CGGA_P128 | 1223 | Alive | Primary | LGG | WHO II | Male | < 50 | Yes | No | IDH1 Mutant | Non-codel |
| CGGA_P132 | 1240 | Alive | Primary | LGG | WHO II | Male | < 50 | Yes | No | IDH1 Mutant | Codel |
| CGGA_P150 | 349 | Alive | Primary | LGG | WHO II | Male | < 50 | Yes | No | IDH1 Mutant | Non-codel |
| CGGA_P155 | 1121 | Alive | Primary | LGG | WHO II | Male | 50-59 | Yes | No | IDH WT | Non-codel |
| CGGA_P158 | 1148 | Alive | Primary | LGG | WHO II | Male | < 50 | Yes | No | IDH1 Mutant | NA |
| CGGA_P173 | 1086 | Alive | Primary | LGG | WHO II | Male | < 50 | Yes | No | IDH1 Mutant | Non-codel |
| CGGA_P176 | 804 | Alive | Primary | LGG | WHO II | Female | < 50 | Yes | No | IDH1 Mutant | Codel |
| CGGA_P177 | 1080 | Alive | Primary | LGG | WHO II | Male | < 50 | Yes | No | IDH WT | Non-codel |
| CGGA_P19 | 1555 | Alive | Primary | LGG | WHO II | Male | < 50 | Yes | No | IDH1 Mutant | Non-codel |
| CGGA_P20 | 1554 | Alive | Primary | LGG | WHO II | Female | 50-59 | Yes | No | IDH1 Mutant | Non-codel |
| CGGA_P27 | 413 | Alive | Primary | LGG | WHO II | Female | < 50 | Yes | No | IDH1 Mutant | Non-codel |
| CGGA_P271 | 1014 | Alive | Primary | LGG | WHO II | Male | < 50 | Yes | No | IDH1 Mutant | NA |
| CGGA_P298 | 634 | Alive | Recurrent | rLGG | WHO III | Female | 50-59 | Yes | No | IDH1 Mutant | Codel |
| CGGA_P3 | 1757 | Alive | Recurrent | rLGG | WHO III | Male | < 50 | Yes | No | IDH1 Mutant | NA |
| CGGA_P316 | 567 | Alive | Recurrent | rLGG | WHO II | Male | < 50 | Yes | No | NA | Non-codel |
| CGGA_P84 | 1326 | Alive | Primary | LGG | WHO II | Female | < 50 | Yes | No | IDH1 Mutant | Non-codel |
| CGGA_P86 | 1326 | Alive | Primary | LGG | WHO II | Female | < 50 | Yes | No | IDH1 Mutant | Codel |
| CGGA_1004 | 899 | Dead | Primary | LGG | WHO II | Female | 60-69 | Yes | No | IDH WT | Non-codel |
| CGGA_1011 | 109 | Dead | Primary | GBM | WHO IV | Female | < 50 | Yes | No | IDH WT | Non-codel |
| CGGA_1015 | 164 | Dead | Primary | GBM | WHO IV | Male | 60-69 | Yes | No | IDH WT | Non-codel |
| CGGA_1017 | 768 | Dead | Primary | GBM | WHO IV | Female | < 50 | Yes | No | IDH WT | Non-codel |
| CGGA_1020 | 1120 | Dead | Primary | LGG | WHO II | Female | < 50 | Yes | No | IDH1 Mutant | Non-codel |
| CGGA_103 | 1188 | Dead | Primary | LGG | WHO III | Male | 50-59 | Yes | No | IDH1 Mutant | Non-codel |
| CGGA_1034 | 753 | Dead | Primary | LGG | WHO III | Male | < 50 | Yes | No | IDH WT | Non-codel |
| CGGA_1059 | 21 | Dead | Recurrent | rLGG | WHO II | Female | 50-59 | Yes | No | IDH1 Mutant | Non-codel |
| CGGA_1066 | 723 | Dead | Primary | LGG | WHO II | Female | < 50 | Yes | No | IDH1 Mutant | Non-codel |
| CGGA_1070 | 333 | Dead | Primary | GBM | WHO IV | Male | < 50 | Yes | No | IDH WT | Non-codel |
| CGGA_1073 | 270 | Dead | Primary | GBM | WHO IV | Female | < 50 | Yes | No | IDH WT | Non-codel |
| CGGA_1077 | 591 | Dead | Primary | GBM | WHO IV | Male | < 50 | Yes | No | IDH WT | Non-codel |
| CGGA_1081 | 216 | Dead | Recurrent | rLGG | WHO III | Female | < 50 | Yes | No | IDH WT | Non-codel |
| CGGA_1129 | 168 | Dead | Secondary | sGBM | WHO IV | Male | < 50 | Yes | No | IDH1 Mutant | Non-codel |
| CGGA_1130 | 402 | Dead | Recurrent | rGBM | WHO IV | Female | < 50 | Yes | No | IDH1 Mutant | Non-codel |
| CGGA_1227 | 1197 | Dead | Recurrent | rGBM | WHO IV | Female | < 50 | Yes | No | IDH WT | Non-codel |
| CGGA_1262 | 166 | Dead | Recurrent | rGBM | WHO IV | Female | 60-69 | Yes | No | IDH WT | NA |
| CGGA_1263 | 772 | Dead | Primary | LGG | WHO III | Male | 50-59 | Yes | No | IDH WT | Non-codel |
| CGGA_1326 | 322 | Dead | Primary | GBM | WHO IV | Male | < 50 | Yes | No | IDH1 Mutant | Non-codel |
| CGGA_135 | 1924 | Dead | Primary | LGG | WHO III | Female | < 50 | Yes | No | IDH WT | Non-codel |
| CGGA_1389 | 1669 | Dead | Primary | LGG | WHO III | Female | 60-69 | Yes | No | IDH1 Mutant | Codel |
| CGGA_1435 | 1657 | Dead | Primary | LGG | WHO III | Female | < 50 | Yes | No | IDH1 Mutant | Non-codel |
| CGGA_1467 | 866 | Dead | Primary | GBM | WHO IV | Male | 50-59 | Yes | No | IDH1 Mutant | NA |
| CGGA_1543 | 723 | Dead | Primary | GBM | WHO IV | Male | 50-59 | Yes | No | IDH1 Mutant | Non-codel |
| CGGA_1575 | 129 | Dead | Recurrent | rLGG | WHO III | Male | < 50 | Yes | No | IDH1 Mutant | Non-codel |
| CGGA_1631 | 221 | Dead | Recurrent | rGBM | WHO IV | Female | 50-59 | Yes | No | IDH WT | Non-codel |
| CGGA_1664 | 1528 | Dead | Primary | LGG | WHO II | Female | < 50 | Yes | No | IDH1 Mutant | Non-codel |
| CGGA_194 | 2304 | Dead | Recurrent | rLGG | WHO III | Female | < 50 | Yes | No | IDH1 Mutant | Codel |
| CGGA_272 | 217 | Dead | Secondary | sGBM | WHO IV | Male | 50-59 | Yes | No | IDH WT | Non-codel |
| CGGA_273 | 2635 | Dead | Primary | LGG | WHO II | Female | < 50 | Yes | No | IDH1 Mutant | Non-codel |
| CGGA_28 | 1103 | Dead | Primary | LGG | WHO II | Male | < 50 | Yes | No | IDH1 Mutant | Codel |
| CGGA_288 | 1057 | Dead | Recurrent | rLGG | WHO II | Male | < 50 | Yes | No | IDH1 Mutant | Codel |
| CGGA_400 | 1321 | Dead | Recurrent | rLGG | WHO III | Female | < 50 | Yes | No | IDH1 Mutant | Non-codel |
| CGGA_499 | 122 | Dead | Primary | GBM | WHO IV | Male | 50-59 | Yes | No | IDH WT | Non-codel |
| CGGA_518 | 212 | Dead | Secondary | sGBM | WHO IV | Male | < 50 | Yes | No | IDH1 Mutant | NA |
| CGGA_525 | 138 | Dead | Primary | GBM | WHO IV | Male | 60-69 | Yes | No | IDH WT | Non-codel |
| CGGA_554 | 134 | Dead | Recurrent | rLGG | WHO III | Female | < 50 | Yes | No | NA | Non-codel |
| CGGA_580 | 1394 | Dead | Primary | LGG | WHO II | Male | < 50 | Yes | No | IDH1 Mutant | Non-codel |
| CGGA_583 | 3107 | Dead | Primary | LGG | WHO II | Female | < 50 | Yes | No | IDH1 Mutant | NA |
| CGGA_599 | 418 | Dead | Primary | LGG | WHO III | Female | 60-69 | Yes | No | IDH WT | Non-codel |
| CGGA_601 | 1133 | Dead | Primary | LGG | WHO II | Male | < 50 | Yes | No | IDH1 Mutant | Non-codel |
| CGGA_619 | 550 | Dead | Recurrent | rLGG | WHO II | Male | < 50 | Yes | No | IDH WT | Non-codel |
| CGGA_658 | 372 | Dead | Primary | GBM | WHO IV | Male | 50-59 | Yes | No | IDH WT | Non-codel |
| CGGA_666 | 2499 | Dead | Primary | LGG | WHO II | Female | < 50 | Yes | No | IDH1 Mutant | Codel |
| CGGA_676 | 376 | Dead | Primary | GBM | WHO IV | Male | 60-69 | Yes | No | IDH WT | Non-codel |
| CGGA_679 | 263 | Dead | Primary | GBM | WHO IV | Female | 60-69 | Yes | No | IDH WT | Non-codel |
| CGGA_705 | 1507 | Dead | Primary | LGG | WHO III | Male | < 50 | Yes | No | IDH1 Mutant | Non-codel |
| CGGA_720 | 1258 | Dead | Recurrent | rLGG | WHO II | Female | < 50 | Yes | No | IDH1 Mutant | Non-codel |
| CGGA_730 | 2118 | Dead | Recurrent | rLGG | WHO II | Female | 50-59 | Yes | No | IDH1 Mutant | Codel |
| CGGA_731 | 503 | Dead | Primary | GBM | WHO IV | Male | 50-59 | Yes | No | IDH WT | Non-codel |
| CGGA_747 | 970 | Dead | Primary | GBM | WHO IV | Male | < 50 | Yes | No | IDH1 Mutant | Non-codel |
| CGGA_761 | 1840 | Dead | Primary | GBM | WHO IV | Male | < 50 | Yes | No | IDH1 Mutant | Non-codel |
| CGGA_777 | 146 | Dead | Recurrent | rGBM | WHO IV | Male | < 50 | Yes | No | IDH WT | Non-codel |
| CGGA_782 | 289 | Dead | Primary | GBM | WHO IV | Male | 60-69 | Yes | No | IDH WT | Non-codel |
| CGGA_785 | 3517 | Dead | Primary | LGG | WHO II | Male | < 50 | Yes | No | IDH1 Mutant | Codel |
| CGGA_789 | 345 | Dead | Primary | GBM | WHO IV | Male | 50-59 | Yes | No | IDH WT | Non-codel |
| CGGA_791 | 252 | Dead | Primary | LGG | WHO III | Male | 50-59 | Yes | No | IDH WT | Non-codel |
| CGGA_815 | 2219 | Dead | Primary | LGG | WHO II | Female | 50-59 | Yes | No | IDH1 Mutant | Non-codel |
| CGGA_852 | 169 | Dead | Primary | LGG | WHO III | Male | < 50 | Yes | No | IDH WT | Non-codel |
| CGGA_859 | 387 | Dead | Primary | GBM | WHO IV | Male | 50-59 | Yes | No | IDH WT | Non-codel |
| CGGA_863 | 2073 | Dead | Primary | LGG | WHO II | Female | < 50 | Yes | No | IDH WT | Non-codel |
| CGGA_893 | 3160 | Dead | Primary | LGG | WHO II | Female | < 50 | Yes | No | IDH1 Mutant | Non-codel |
| CGGA_903 | 1455 | Dead | Primary | LGG | WHO III | Female | < 50 | Yes | No | IDH1 Mutant | Non-codel |
| CGGA_904 | 774 | Dead | Primary | LGG | WHO II | Male | < 50 | Yes | No | IDH1 Mutant | Non-codel |
| CGGA_905 | 3174 | Dead | Primary | LGG | WHO II | Male | < 50 | Yes | No | IDH1 Mutant | Non-codel |
| CGGA_D03 | 423 | Dead | Primary | GBM | WHO IV | Male | < 50 | Yes | No | IDH WT | Non-codel |
| CGGA_D09 | 233 | Dead | Primary | GBM | WHO IV | Male | 50-59 | Yes | No | IDH WT | Non-codel |
| CGGA_D16 | 261 | Dead | Primary | LGG | WHO III | Female | 50-59 | Yes | No | IDH WT | Non-codel |
| CGGA_D21 | 1208 | Dead | Primary | LGG | WHO III | Female | < 50 | Yes | No | IDH1 Mutant | Non-codel |
| CGGA_D24 | 837 | Dead | Primary | LGG | WHO III | Female | < 50 | Yes | No | IDH1 Mutant | Non-codel |
| CGGA_D26 | 263 | Dead | Recurrent | rGBM | WHO IV | Male | 50-59 | Yes | No | IDH WT | Non-codel |
| CGGA_D28 | 860 | Dead | Primary | LGG | WHO III | Male | < 50 | Yes | No | IDH1 Mutant | Non-codel |
| CGGA_D30 | 215 | Dead | Primary | GBM | WHO IV | Male | 70-79 | Yes | No | IDH WT | Non-codel |
| CGGA_D35 | 661 | Dead | Primary | GBM | WHO IV | Female | 60-69 | Yes | No | IDH WT | Non-codel |
| CGGA_D38 | 689 | Dead | Recurrent | rGBM | WHO IV | Female | < 50 | Yes | No | IDH1 Mutant | Codel |
| CGGA_D44 | 313 | Dead | Primary | LGG | WHO III | Female | < 50 | Yes | No | IDH WT | Non-codel |
| CGGA_D48 | 221 | Dead | Recurrent | rLGG | WHO III | Female | < 50 | Yes | No | IDH1 Mutant | Non-codel |
| CGGA_D51 | 135 | Dead | Recurrent | rGBM | WHO IV | Female | 60-69 | Yes | No | IDH WT | Non-codel |
| CGGA_D56 | 64 | Dead | Recurrent | rLGG | WHO III | Male | < 50 | Yes | No | IDH1 Mutant | Non-codel |
| CGGA_P145 | 308 | Dead | Primary | LGG | WHO II | Male | 50-59 | Yes | No | IDH1 Mutant | Non-codel |
| CGGA_P147 | 863 | Dead | Primary | LGG | WHO II | Male | 50-59 | Yes | No | IDH1 Mutant | Non-codel |
| CGGA_P315 | 451 | Dead | Recurrent | rLGG | WHO II | Female | < 50 | Yes | No | IDH1 Mutant | Non-codel |
| CGGA_P7 | 110 | Dead | Recurrent | rGBM | WHO IV | Male | < 50 | Yes | No | IDH WT | Non-codel |
| CGGA_P358 | 808 | Alive | Recurrent | rLGG | WHO II | Male | < 50 | NA | Yes | IDH1 Mutant | Non-codel |
| CGGA_2047 | 598 | Dead | Recurrent | rGBM | WHO IV | Female | 60-69 | NA | Yes | IDH WT | Non-codel |
| CGGA_901 | 929 | Dead | Primary | LGG | WHO III | Male | < 50 | NA | Yes | IDH1 Mutant | Non-codel |
| CGGA_1001 | 3428 | Alive | Primary | GBM | WHO IV | Male | < 50 | No | Yes | IDH WT | Non-codel |
| CGGA_1003 | 3428 | Alive | Primary | LGG | WHO II | Female | < 50 | No | Yes | IDH1 Mutant | Codel |
| CGGA_1022 | 518 | Alive | Recurrent | rLGG | WHO II | Female | 60-69 | No | Yes | IDH WT | Non-codel |
| CGGA_1033 | 1322 | Alive | Primary | LGG | WHO II | Male | < 50 | No | Yes | NA | Non-codel |
| CGGA_1046 | 2829 | Alive | Primary | LGG | WHO II | Male | < 50 | No | Yes | NA | NA |
| CGGA_1048 | 703 | Alive | Primary | LGG | WHO II | Female | < 50 | No | Yes | IDH WT | Non-codel |
| CGGA_106 | 4697 | Alive | Recurrent | rLGG | WHO II | Male | < 50 | No | Yes | IDH WT | Non-codel |
| CGGA_1087 | 701 | Alive | Primary | LGG | WHO II | Female | < 50 | No | Yes | IDH1 Mutant | Codel |
| CGGA_1147 | 2826 | Alive | Primary | LGG | WHO II | Male | < 50 | No | Yes | IDH1 Mutant | Non-codel |
| CGGA_1159 | 2792 | Alive | Primary | LGG | WHO II | Male | 50-59 | No | Yes | IDH1 Mutant | Non-codel |
| CGGA_1198 | 2679 | Alive | Primary | LGG | WHO II | Male | < 50 | No | Yes | IDH1 Mutant | NA |
| CGGA_1204 | 2668 | Alive | Primary | LGG | WHO II | Male | 50-59 | No | Yes | IDH1 Mutant | Codel |
| CGGA_1207 | 2661 | Alive | Primary | LGG | WHO III | Female | < 50 | No | Yes | NA | Codel |
| CGGA_1228 | 2626 | Alive | Primary | LGG | WHO II | Male | < 50 | No | Yes | IDH WT | Non-codel |
| CGGA_1235 | 2616 | Alive | Primary | LGG | WHO III | Female | < 50 | No | Yes | IDH1 Mutant | Non-codel |
| CGGA_1264 | 2541 | Alive | Primary | LGG | WHO III | Female | < 50 | No | Yes | IDH1 Mutant | Codel |
| CGGA_1269 | 2535 | Alive | Primary | LGG | WHO III | Female | < 50 | No | Yes | IDH1 Mutant | Codel |
| CGGA_1317 | 2476 | Alive | Primary | LGG | WHO II | Male | < 50 | No | Yes | IDH1 Mutant | NA |
| CGGA_1332 | 510 | Alive | Primary | GBM | WHO IV | Female | 50-59 | No | Yes | IDH WT | Non-codel |
| CGGA_1335 | 2451 | Alive | Primary | LGG | WHO II | Female | < 50 | No | Yes | IDH1 Mutant | Codel |
| CGGA_1339 | 2444 | Alive | Primary | LGG | WHO II | Male | < 50 | No | Yes | IDH1 Mutant | Codel |
| CGGA_1350 | 2431 | Alive | Primary | LGG | WHO II | Male | < 50 | No | Yes | IDH1 Mutant | NA |
| CGGA_1370 | 325 | Alive | Recurrent | rGBM | WHO IV | Male | < 50 | No | Yes | IDH WT | Non-codel |
| CGGA_1407 | 2340 | Alive | Primary | LGG | WHO III | Female | < 50 | No | Yes | IDH1 Mutant | Codel |
| CGGA_1440 | 2289 | Alive | Primary | LGG | WHO II | Female | < 50 | No | Yes | IDH1 Mutant | Codel |
| CGGA_1446 | 2283 | Alive | Primary | LGG | WHO II | Male | < 50 | No | Yes | IDH1 Mutant | NA |
| CGGA_1594 | 480 | Alive | Recurrent | rLGG | WHO II | Male | < 50 | No | Yes | NA | Non-codel |
| CGGA_1850 | 750 | Alive | Primary | LGG | WHO II | Male | < 50 | No | Yes | IDH1 Mutant | Non-codel |
| CGGA_2053 | 640 | Alive | Recurrent | rGBM | WHO IV | Male | 50-59 | No | Yes | IDH WT | Codel |
| CGGA_846 | 2991 | Alive | Recurrent | rLGG | WHO II | Female | < 50 | No | Yes | IDH WT | Non-codel |
| CGGA_P31 | 1514 | Alive | Primary | LGG | WHO II | Male | < 50 | No | Yes | IDH WT | Non-codel |
| CGGA_P392 | 741 | Alive | Recurrent | rLGG | WHO II | Male | < 50 | No | Yes | IDH1 Mutant | Codel |
| CGGA_1014 | 263 | Dead | Primary | LGG | WHO II | Male | < 50 | No | Yes | IDH WT | Non-codel |
| CGGA_1019 | 212 | Dead | Recurrent | rGBM | WHO IV | Male | 60-69 | No | Yes | IDH WT | Non-codel |
| CGGA_1031 | 2457 | Dead | Primary | LGG | WHO II | Female | < 50 | No | Yes | IDH1 Mutant | Non-codel |
| CGGA_1039 | 727 | Dead | Primary | GBM | WHO IV | Male | < 50 | No | Yes | IDH WT | Non-codel |
| CGGA_1060 | 227 | Dead | Recurrent | rGBM | WHO IV | Male | < 50 | No | Yes | IDH1 Mutant | Non-codel |
| CGGA_1094 | 252 | Dead | Recurrent | rLGG | WHO III | Female | < 50 | No | Yes | IDH1 Mutant | Non-codel |
| CGGA_1099 | 122 | Dead | Secondary | sGBM | WHO IV | Male | < 50 | No | Yes | IDH WT | Non-codel |
| CGGA_1113 | 1265 | Dead | Primary | LGG | WHO III | Male | < 50 | No | Yes | IDH1 Mutant | Codel |
| CGGA_1134 | 59 | Dead | Primary | GBM | WHO IV | Female | 50-59 | No | Yes | IDH WT | Non-codel |
| CGGA_1136 | 236 | Dead | Secondary | sGBM | WHO IV | Female | < 50 | No | Yes | IDH1 Mutant | Non-codel |
| CGGA_1140 | 921 | Dead | Recurrent | rLGG | WHO III | Female | < 50 | No | Yes | IDH WT | Non-codel |
| CGGA_1164 | 1022 | Dead | Recurrent | rGBM | WHO IV | Female | < 50 | No | Yes | IDH1 Mutant | Codel |
| CGGA_1175 | 441 | Dead | Secondary | sGBM | WHO IV | Female | < 50 | No | Yes | IDH1 Mutant | Non-codel |
| CGGA_1218 | 286 | Dead | Secondary | sGBM | WHO IV | Male | 50-59 | No | Yes | IDH WT | Non-codel |
| CGGA_1272 | 124 | Dead | Secondary | sGBM | WHO IV | Male | < 50 | No | Yes | IDH1 Mutant | Non-codel |
| CGGA_1284 | 97 | Dead | Primary | LGG | WHO III | Male | 70-79 | No | Yes | IDH WT | Non-codel |
| CGGA_1320 | 59 | Dead | Primary | GBM | WHO IV | Male | < 50 | No | Yes | IDH WT | Non-codel |
| CGGA_1356 | 948 | Dead | Recurrent | rLGG | WHO III | Male | 50-59 | No | Yes | IDH1 Mutant | Non-codel |
| CGGA_1546 | 223 | Dead | Primary | GBM | WHO IV | Male | 50-59 | No | Yes | IDH WT | Non-codel |
| CGGA_1559 | 603 | Dead | Primary | GBM | WHO IV | Male | 60-69 | No | Yes | IDH1 Mutant | Codel |
| CGGA_1686 | 745 | Dead | Primary | LGG | WHO III | Male | < 50 | No | Yes | IDH WT | Non-codel |
| CGGA_1770 | 267 | Dead | Recurrent | rGBM | WHO IV | Female | < 50 | No | Yes | IDH1 Mutant | Non-codel |
| CGGA_507 | 302 | Dead | Recurrent | rLGG | WHO III | Female | 50-59 | No | Yes | IDH1 Mutant | Non-codel |
| CGGA_530 | 506 | Dead | Recurrent | rGBM | WHO IV | Female | < 50 | No | Yes | IDH WT | Non-codel |
| CGGA_568 | 285 | Dead | Recurrent | rGBM | WHO IV | Male | < 50 | No | Yes | IDH WT | Non-codel |
| CGGA_710 | 660 | Dead | Primary | GBM | WHO IV | Male | < 50 | No | Yes | IDH1 Mutant | Non-codel |
| CGGA_780 | 923 | Dead | Recurrent | rLGG | WHO III | Female | 60-69 | No | Yes | IDH WT | Non-codel |
| CGGA_878 | 863 | Dead | Primary | GBM | WHO IV | Male | < 50 | No | Yes | IDH WT | Non-codel |
| CGGA_899 | 231 | Dead | Recurrent | rGBM | WHO IV | Male | < 50 | No | Yes | IDH1 Mutant | Non-codel |
| CGGA_D32 | 358 | Dead | Recurrent | rGBM | WHO IV | Male | 60-69 | No | Yes | IDH WT | Non-codel |
| CGGA_D46 | 300 | Dead | Recurrent | rLGG | WHO III | Female | < 50 | No | Yes | IDH1 Mutant | Non-codel |
| CGGA_P18 | 387 | Dead | Primary | LGG | WHO III | Male | < 50 | No | Yes | IDH1 Mutant | Non-codel |
| CGGA_P29 | 103 | Dead | Recurrent | rLGG | WHO III | Female | < 50 | No | Yes | IDH1 Mutant | NA |
| CGGA_1012 | 3407 | Alive | Recurrent | rLGG | WHO II | Male | < 50 | Yes | Yes | IDH1 Mutant | Non-codel |
| CGGA_1027 | 2688 | Alive | Primary | LGG | WHO III | Male | < 50 | Yes | Yes | IDH1 Mutant | Codel |
| CGGA_1030 | 3275 | Alive | Primary | LGG | WHO II | Female | < 50 | Yes | Yes | IDH1 Mutant | Non-codel |
| CGGA_1041 | 3204 | Alive | Primary | GBM | WHO IV | Male | 50-59 | Yes | Yes | IDH WT | Non-codel |
| CGGA_1065 | 3023 | Alive | Primary | LGG | WHO II | Female | < 50 | Yes | Yes | IDH1 Mutant | Codel |
| CGGA_1100 | 2925 | Alive | Primary | LGG | WHO II | Female | 60-69 | Yes | Yes | IDH1 Mutant | NA |
| CGGA_1121 | 2889 | Alive | Recurrent | rLGG | WHO III | Female | < 50 | Yes | Yes | IDH1 Mutant | Non-codel |
| CGGA_1131 | 2862 | Alive | Primary | LGG | WHO III | Female | < 50 | Yes | Yes | IDH1 Mutant | Non-codel |
| CGGA_1132 | 2862 | Alive | Recurrent | rLGG | WHO III | Female | < 50 | Yes | Yes | IDH1 Mutant | Codel |
| CGGA_1137 | 2848 | Alive | Primary | LGG | WHO III | Male | 50-59 | Yes | Yes | IDH WT | NA |
| CGGA_1144 | 2505 | Alive | Primary | LGG | WHO III | Female | < 50 | Yes | Yes | IDH WT | Non-codel |
| CGGA_1146 | 2498 | Alive | Primary | LGG | WHO III | Male | < 50 | Yes | Yes | IDH1 Mutant | Non-codel |
| CGGA_1152 | 2812 | Alive | Primary | LGG | WHO II | Male | < 50 | Yes | Yes | IDH WT | Non-codel |
| CGGA_1158 | 2799 | Alive | Primary | LGG | WHO II | Male | < 50 | Yes | Yes | IDH1 Mutant | Codel |
| CGGA_1162 | 2785 | Alive | Primary | LGG | WHO III | Male | < 50 | Yes | Yes | NA | Codel |
| CGGA_1172 | 2744 | Alive | Primary | GBM | WHO IV | Female | < 50 | Yes | Yes | IDH1 Mutant | Non-codel |
| CGGA_1180 | 1226 | Alive | Primary | GBM | WHO IV | Female | < 50 | Yes | Yes | IDH WT | Non-codel |
| CGGA_1181 | 318 | Alive | Primary | LGG | WHO II | Male | < 50 | Yes | Yes | IDH1 Mutant | Non-codel |
| CGGA_1189 | 2695 | Alive | Primary | LGG | WHO III | Female | < 50 | Yes | Yes | IDH1 Mutant | Codel |
| CGGA_1190 | 2695 | Alive | Primary | LGG | WHO III | Female | < 50 | Yes | Yes | IDH1 Mutant | Non-codel |
| CGGA_1192 | 2693 | Alive | Primary | LGG | WHO II | Female | < 50 | Yes | Yes | IDH1 Mutant | Non-codel |
| CGGA_1239 | 2609 | Alive | Primary | LGG | WHO II | Female | < 50 | Yes | Yes | IDH1 Mutant | Codel |
| CGGA_1246 | 2584 | Alive | Primary | LGG | WHO III | Male | 50-59 | Yes | Yes | IDH1 Mutant | Codel |
| CGGA_1256 | 2557 | Alive | Primary | GBM | WHO IV | Female | 50-59 | Yes | Yes | NA | Non-codel |
| CGGA_1273 | 2438 | Alive | Recurrent | rLGG | WHO III | Male | 60-69 | Yes | Yes | IDH1 Mutant | Non-codel |
| CGGA_1281 | 2521 | Alive | Primary | LGG | WHO III | Male | < 50 | Yes | Yes | IDH WT | Codel |
| CGGA_1286 | 2518 | Alive | Primary | LGG | WHO II | Female | 50-59 | Yes | Yes | IDH1 Mutant | Codel |
| CGGA_1291 | 2422 | Alive | Primary | LGG | WHO III | Male | < 50 | Yes | Yes | NA | Codel |
| CGGA_1292 | 2513 | Alive | Primary | LGG | WHO III | Male | < 50 | Yes | Yes | IDH WT | Non-codel |
| CGGA_1303 | 2473 | Alive | Primary | LGG | WHO III | Female | < 50 | Yes | Yes | IDH1 Mutant | NA |
| CGGA_1305 | 2493 | Alive | Primary | LGG | WHO III | Male | < 50 | Yes | Yes | IDH1 Mutant | Non-codel |
| CGGA_1307 | 965 | Alive | Primary | LGG | WHO III | Female | < 50 | Yes | Yes | IDH1 Mutant | Non-codel |
| CGGA_1309 | 964 | Alive | Primary | LGG | WHO III | Male | 60-69 | Yes | Yes | NA | Codel |
| CGGA_1319 | 2473 | Alive | Primary | LGG | WHO III | Female | < 50 | Yes | Yes | IDH WT | Non-codel |
| CGGA_1330 | 2457 | Alive | Primary | LGG | WHO II | Male | < 50 | Yes | Yes | IDH1 Mutant | Non-codel |
| CGGA_1361 | 2416 | Alive | Primary | LGG | WHO II | Male | < 50 | Yes | Yes | IDH1 Mutant | Non-codel |
| CGGA_1362 | 2416 | Alive | Primary | LGG | WHO III | Female | 60-69 | Yes | Yes | IDH WT | Non-codel |
| CGGA_1369 | 360 | Alive | Primary | LGG | WHO III | Female | < 50 | Yes | Yes | IDH1 Mutant | Non-codel |
| CGGA_1371 | 2403 | Alive | Primary | GBM | WHO IV | Male | 60-69 | Yes | Yes | IDH WT | Non-codel |
| CGGA_1377 | 2392 | Alive | Primary | LGG | WHO III | Male | < 50 | Yes | Yes | IDH1 Mutant | Non-codel |
| CGGA_1386 | 2382 | Alive | Primary | LGG | WHO III | Male | < 50 | Yes | Yes | NA | Codel |
| CGGA_1390 | 2050 | Alive | Recurrent | rLGG | WHO III | Male | 50-59 | Yes | Yes | IDH1 Mutant | Non-codel |
| CGGA_1391 | 426 | Alive | Primary | GBM | WHO IV | Male | 60-69 | Yes | Yes | IDH WT | Non-codel |
| CGGA_1400 | 2354 | Alive | Recurrent | rLGG | WHO III | Female | 50-59 | Yes | Yes | NA | Codel |
| CGGA_1402 | 2354 | Alive | Primary | GBM | WHO IV | Male | < 50 | Yes | Yes | IDH WT | Non-codel |
| CGGA_1409 | 2337 | Alive | Primary | GBM | WHO IV | Female | < 50 | Yes | Yes | IDH1 Mutant | Non-codel |
| CGGA_1413 | 2339 | Alive | Primary | LGG | WHO III | Male | < 50 | Yes | Yes | IDH1 Mutant | Codel |
| CGGA_1415 | 2338 | Alive | Recurrent | rGBM | WHO IV | Female | < 50 | Yes | Yes | IDH WT | Non-codel |
| CGGA_1417 | 2332 | Alive | Primary | LGG | WHO III | Male | 50-59 | Yes | Yes | IDH1 Mutant | Codel |
| CGGA_1421 | 2318 | Alive | Primary | LGG | WHO III | Male | < 50 | Yes | Yes | NA | Non-codel |
| CGGA_1424 | 2311 | Alive | Primary | LGG | WHO III | Female | < 50 | Yes | Yes | IDH1 Mutant | NA |
| CGGA_1429 | 2304 | Alive | Recurrent | rGBM | WHO IV | Female | < 50 | Yes | Yes | IDH1 Mutant | Codel |
| CGGA_1445 | 2283 | Alive | Recurrent | rLGG | WHO III | Male | < 50 | Yes | Yes | IDH WT | NA |
| CGGA_1469 | 2245 | Alive | Primary | LGG | WHO III | Male | < 50 | Yes | Yes | IDH1 Mutant | NA |
| CGGA_1471 | 2249 | Alive | Primary | LGG | WHO II | Male | < 50 | Yes | Yes | IDH1 Mutant | Non-codel |
| CGGA_1474 | 333 | Alive | Primary | LGG | WHO III | Female | 50-59 | Yes | Yes | IDH1 Mutant | NA |
| CGGA_1482 | 2212 | Alive | Primary | LGG | WHO III | Male | < 50 | Yes | Yes | NA | Codel |
| CGGA_1504 | 2178 | Alive | Primary | LGG | WHO III | Female | < 50 | Yes | Yes | IDH1 Mutant | Non-codel |
| CGGA_1513 | 2151 | Alive | Recurrent | rLGG | WHO III | Female | < 50 | Yes | Yes | IDH1 Mutant | Codel |
| CGGA_1516 | 2150 | Alive | Primary | LGG | WHO II | Male | < 50 | Yes | Yes | IDH1 Mutant | Codel |
| CGGA_1523 | 2141 | Alive | Primary | LGG | WHO II | Male | 50-59 | Yes | Yes | IDH1 Mutant | Non-codel |
| CGGA_1526 | 2122 | Alive | Primary | LGG | WHO III | Male | < 50 | Yes | Yes | IDH1 Mutant | Codel |
| CGGA_1530 | 2117 | Alive | Primary | LGG | WHO III | Male | < 50 | Yes | Yes | NA | Non-codel |
| CGGA_1539 | 2088 | Alive | Primary | GBM | WHO IV | Male | 60-69 | Yes | Yes | IDH1 Mutant | Non-codel |
| CGGA_1542 | 184 | Alive | Primary | GBM | WHO IV | Male | < 50 | Yes | Yes | IDH1 Mutant | NA |
| CGGA_1552 | 2052 | Alive | Primary | LGG | WHO III | Male | < 50 | Yes | Yes | IDH1 Mutant | Codel |
| CGGA_1557 | 2047 | Alive | Primary | LGG | WHO III | Male | < 50 | Yes | Yes | IDH1 Mutant | Codel |
| CGGA_1566 | 2040 | Alive | Primary | LGG | WHO III | Female | < 50 | Yes | Yes | IDH1 Mutant | Codel |
| CGGA_1571 | 412 | Alive | Primary | GBM | WHO IV | Female | < 50 | Yes | Yes | IDH1 Mutant | Non-codel |
| CGGA_1595 | 1983 | Alive | Primary | GBM | WHO IV | Female | < 50 | Yes | Yes | NA | Non-codel |
| CGGA_1613 | 250 | Alive | Primary | GBM | WHO IV | Male | 50-59 | Yes | Yes | IDH WT | Non-codel |
| CGGA_1618 | 1939 | Alive | Primary | LGG | WHO III | Male | < 50 | Yes | Yes | IDH1 Mutant | Non-codel |
| CGGA_1620 | 1932 | Alive | Primary | LGG | WHO III | Male | 50-59 | Yes | Yes | IDH1 Mutant | Codel |
| CGGA_1627 | 1921 | Alive | Primary | LGG | WHO III | Male | < 50 | Yes | Yes | IDH1 Mutant | Non-codel |
| CGGA_1640 | 1886 | Alive | Primary | LGG | WHO III | Male | 50-59 | Yes | Yes | NA | Non-codel |
| CGGA_1645 | 1857 | Alive | Primary | LGG | WHO III | Male | 50-59 | Yes | Yes | IDH1 Mutant | Non-codel |
| CGGA_1647 | 1848 | Alive | Primary | LGG | WHO II | Male | < 50 | Yes | Yes | NA | Codel |
| CGGA_1648 | 1747 | Alive | Primary | LGG | WHO III | Female | < 50 | Yes | Yes | NA | Codel |
| CGGA_1649 | 1844 | Alive | Primary | LGG | WHO II | Male | < 50 | Yes | Yes | IDH1 Mutant | Codel |
| CGGA_1660 | 433 | Alive | Primary | LGG | WHO II | Male | < 50 | Yes | Yes | IDH WT | Codel |
| CGGA_1661 | 1829 | Alive | Primary | LGG | WHO III | Female | < 50 | Yes | Yes | NA | Non-codel |
| CGGA_1667 | 1800 | Alive | Primary | LGG | WHO III | Female | < 50 | Yes | Yes | IDH1 Mutant | Codel |
| CGGA_1671 | 1788 | Alive | Primary | LGG | WHO III | Female | < 50 | Yes | Yes | IDH1 Mutant | Non-codel |
| CGGA_1675 | 1774 | Alive | Primary | LGG | WHO III | Male | < 50 | Yes | Yes | IDH1 Mutant | Non-codel |
| CGGA_1679 | 1766 | Alive | Primary | LGG | WHO III | Female | 50-59 | Yes | Yes | IDH1 Mutant | Codel |
| CGGA_1687 | 1738 | Alive | Primary | GBM | WHO IV | Male | < 50 | Yes | Yes | IDH WT | Non-codel |
| CGGA_1695 | 1714 | Alive | Recurrent | rLGG | WHO III | Male | < 50 | Yes | Yes | IDH1 Mutant | Non-codel |
| CGGA_1696 | 1205 | Alive | Primary | LGG | WHO III | Male | 70-79 | Yes | Yes | IDH WT | NA |
| CGGA_1699 | 1708 | Alive | Primary | GBM | WHO IV | Female | < 50 | Yes | Yes | IDH1 Mutant | NA |
| CGGA_1700 | 1696 | Alive | Primary | LGG | WHO III | Male | < 50 | Yes | Yes | IDH1 Mutant | Codel |
| CGGA_1701 | 1696 | Alive | Primary | LGG | WHO III | Male | < 50 | Yes | Yes | IDH1 Mutant | Codel |
| CGGA_1703 | 1592 | Alive | Primary | LGG | WHO II | Female | < 50 | Yes | Yes | IDH1 Mutant | Non-codel |
| CGGA_1706 | 1688 | Alive | Primary | GBM | WHO IV | Male | 60-69 | Yes | Yes | IDH WT | Non-codel |
| CGGA_1714 | 1654 | Alive | Primary | LGG | WHO III | Female | < 50 | Yes | Yes | NA | Codel |
| CGGA_1718 | 1616 | Alive | Primary | LGG | WHO III | Female | < 50 | Yes | Yes | IDH1 Mutant | Non-codel |
| CGGA_1720 | 1614 | Alive | Primary | LGG | WHO II | Male | < 50 | Yes | Yes | IDH1 Mutant | Non-codel |
| CGGA_1721 | 1614 | Alive | Primary | LGG | WHO III | Female | < 50 | Yes | Yes | NA | Codel |
| CGGA_1723 | 1606 | Alive | Primary | LGG | WHO III | Female | < 50 | Yes | Yes | IDH1 Mutant | Codel |
| CGGA_1725 | 1606 | Alive | Primary | LGG | WHO III | Female | < 50 | Yes | Yes | IDH1 Mutant | Codel |
| CGGA_1727 | 1600 | Alive | Primary | GBM | WHO IV | Male | < 50 | Yes | Yes | IDH1 Mutant | Non-codel |
| CGGA_1731 | 1596 | Alive | Primary | LGG | WHO III | Female | < 50 | Yes | Yes | IDH1 Mutant | Non-codel |
| CGGA_1737 | 1572 | Alive | Primary | LGG | WHO III | Male | < 50 | Yes | Yes | IDH1 Mutant | Codel |
| CGGA_1744 | 1556 | Alive | Primary | GBM | WHO IV | Male | 50-59 | Yes | Yes | NA | Non-codel |
| CGGA_1745 | 1549 | Alive | Primary | LGG | WHO III | Female | < 50 | Yes | Yes | IDH1 Mutant | NA |
| CGGA_1769 | 1474 | Alive | Primary | GBM | WHO IV | Female | < 50 | Yes | Yes | IDH WT | Non-codel |
| CGGA_1780 | 1446 | Alive | Primary | GBM | WHO IV | Female | 50-59 | Yes | Yes | IDH WT | NA |
| CGGA_1786 | 1437 | Alive | Recurrent | rLGG | WHO III | Female | 50-59 | Yes | Yes | IDH1 Mutant | Codel |
| CGGA_1819 | 1323 | Alive | Primary | GBM | WHO IV | Male | 50-59 | Yes | Yes | IDH WT | Non-codel |
| CGGA_1840 | 1179 | Alive | Primary | GBM | WHO IV | Female | 50-59 | Yes | Yes | IDH WT | Non-codel |
| CGGA_1854 | 1246 | Alive | Primary | LGG | WHO III | Male | < 50 | Yes | Yes | IDH1 Mutant | Non-codel |
| CGGA_1863 | 1214 | Alive | Recurrent | rLGG | WHO III | Female | 50-59 | Yes | Yes | IDH1 Mutant | Codel |
| CGGA_1870 | 1176 | Alive | Primary | GBM | WHO IV | Male | 60-69 | Yes | Yes | IDH1 Mutant | Codel |
| CGGA_1875 | 1178 | Alive | Primary | LGG | WHO II | Female | < 50 | Yes | Yes | IDH WT | Non-codel |
| CGGA_1903 | 1098 | Alive | Primary | LGG | WHO III | Female | < 50 | Yes | Yes | NA | Codel |
| CGGA_1911 | 1075 | Alive | Recurrent | rGBM | WHO IV | Male | < 50 | Yes | Yes | IDH WT | Non-codel |
| CGGA_2003 | 827 | Alive | Recurrent | rGBM | WHO IV | Male | < 50 | Yes | Yes | IDH1 Mutant | Non-codel |
| CGGA_2056 | 628 | Alive | Recurrent | rGBM | WHO IV | Female | < 50 | Yes | Yes | IDH1 Mutant | Non-codel |
| CGGA_2079 | 573 | Alive | Recurrent | rLGG | WHO III | Male | < 50 | Yes | Yes | IDH1 Mutant | Codel |
| CGGA_236 | 706 | Alive | Primary | LGG | WHO II | Male | < 50 | Yes | Yes | IDH1 Mutant | Codel |
| CGGA_254 | 4458 | Alive | Primary | LGG | WHO II | Male | < 50 | Yes | Yes | IDH1 Mutant | Codel |
| CGGA_256 | 158 | Alive | Primary | LGG | WHO II | Male | < 50 | Yes | Yes | IDH1 Mutant | Non-codel |
| CGGA_265 | 3959 | Alive | Recurrent | rLGG | WHO II | Male | < 50 | Yes | Yes | IDH1 Mutant | Non-codel |
| CGGA_274 | 610 | Alive | Primary | GBM | WHO IV | Female | 50-59 | Yes | Yes | IDH WT | Non-codel |
| CGGA_277 | 4089 | Alive | Primary | LGG | WHO III | Male | < 50 | Yes | Yes | IDH1 Mutant | NA |
| CGGA_413 | 183 | Alive | Primary | GBM | WHO IV | Male | 50-59 | Yes | Yes | IDH WT | Non-codel |
| CGGA_420 | 3514 | Alive | Recurrent | rLGG | WHO III | Female | < 50 | Yes | Yes | IDH1 Mutant | Codel |
| CGGA_431 | 4180 | Alive | Recurrent | rLGG | WHO III | Male | < 50 | Yes | Yes | IDH1 Mutant | Codel |
| CGGA_474 | 2029 | Alive | Primary | LGG | WHO III | Male | NA | Yes | Yes | IDH WT | Non-codel |
| CGGA_490 | 3804 | Alive | Primary | LGG | WHO III | Female | < 50 | Yes | Yes | IDH1 Mutant | Codel |
| CGGA_492 | 652 | Alive | Recurrent | rLGG | WHO III | Male | < 50 | Yes | Yes | IDH WT | Non-codel |
| CGGA_494 | 4109 | Alive | Primary | GBM | WHO IV | Female | 50-59 | Yes | Yes | IDH WT | Non-codel |
| CGGA_503 | 4084 | Alive | Primary | LGG | WHO II | Male | 50-59 | Yes | Yes | IDH1 Mutant | Non-codel |
| CGGA_510 | 4077 | Alive | Primary | LGG | WHO III | Male | < 50 | Yes | Yes | IDH1 Mutant | Codel |
| CGGA_541 | 4047 | Alive | Primary | LGG | WHO II | Female | < 50 | Yes | Yes | IDH WT | Non-codel |
| CGGA_565 | 3605 | Alive | Primary | LGG | WHO III | Male | 50-59 | Yes | Yes | IDH WT | Non-codel |
| CGGA_603 | 168 | Alive | Recurrent | rLGG | WHO III | Male | < 50 | Yes | Yes | IDH1 Mutant | Non-codel |
| CGGA_605 | 3971 | Alive | Recurrent | rLGG | WHO III | Male | < 50 | Yes | Yes | IDH1 Mutant | Non-codel |
| CGGA_621 | 1347 | Alive | Recurrent | rLGG | WHO III | Male | < 50 | Yes | Yes | IDH1 Mutant | Non-codel |
| CGGA_635 | 3934 | Alive | Primary | LGG | WHO II | Male | < 50 | Yes | Yes | IDH1 Mutant | Codel |
| CGGA_659 | 730 | Alive | Primary | LGG | WHO II | Female | 50-59 | Yes | Yes | IDH WT | Non-codel |
| CGGA_700 | 170 | Alive | Primary | GBM | WHO IV | Female | < 50 | Yes | Yes | IDH1 Mutant | Non-codel |
| CGGA_796 | 559 | Alive | Primary | LGG | WHO II | Male | 60-69 | Yes | Yes | IDH WT | Non-codel |
| CGGA_804 | 614 | Alive | Primary | GBM | WHO IV | Female | 50-59 | Yes | Yes | IDH WT | Non-codel |
| CGGA_807 | 3710 | Alive | Primary | LGG | WHO II | Male | < 50 | Yes | Yes | IDH WT | Non-codel |
| CGGA_808 | 1167 | Alive | Primary | GBM | WHO IV | Male | < 50 | Yes | Yes | IDH WT | Non-codel |
| CGGA_838 | 3681 | Alive | Primary | LGG | WHO II | Female | < 50 | Yes | Yes | IDH1 Mutant | Codel |
| CGGA_889 | 3613 | Alive | Primary | LGG | WHO II | Male | < 50 | Yes | Yes | IDH1 Mutant | Non-codel |
| CGGA_D27 | 3312 | Alive | Recurrent | rLGG | WHO III | Male | < 50 | Yes | Yes | IDH1 Mutant | Codel |
| CGGA_D45 | 856 | Alive | Recurrent | rLGG | WHO III | Male | < 50 | Yes | Yes | IDH1 Mutant | Non-codel |
| CGGA_D52 | 3191 | Alive | Primary | LGG | WHO III | Male | < 50 | Yes | Yes | IDH1 Mutant | Codel |
| CGGA_D59 | 2778 | Alive | Secondary | sGBM | WHO IV | Female | < 50 | Yes | Yes | IDH1 Mutant | Non-codel |
| CGGA_P108 | 1276 | Alive | Primary | LGG | WHO III | Female | < 50 | Yes | Yes | IDH WT | Non-codel |
| CGGA_P122 | 1274 | Alive | Recurrent | rLGG | WHO II | Male | < 50 | Yes | Yes | IDH1 Mutant | Codel |
| CGGA_P131 | 1233 | Alive | Primary | LGG | WHO III | Female | < 50 | Yes | Yes | IDH1 Mutant | Codel |
| CGGA_P137 | 1164 | Alive | Primary | LGG | WHO II | Male | < 50 | Yes | Yes | IDH1 Mutant | Non-codel |
| CGGA_P142 | 1195 | Alive | Primary | LGG | WHO III | Male | < 50 | Yes | Yes | IDH1 Mutant | Codel |
| CGGA_P144 | 1196 | Alive | Primary | LGG | WHO III | Male | < 50 | Yes | Yes | IDH1 Mutant | Non-codel |
| CGGA_P146 | 344 | Alive | Recurrent | rLGG | WHO II | Male | < 50 | Yes | Yes | IDH1 Mutant | Non-codel |
| CGGA_P151 | 1219 | Alive | Primary | LGG | WHO II | Female | < 50 | Yes | Yes | IDH1 Mutant | Non-codel |
| CGGA_P164 | 1190 | Alive | Primary | GBM | WHO IV | Male | < 50 | Yes | Yes | IDH WT | NA |
| CGGA_P17 | 1561 | Alive | Primary | LGG | WHO III | Male | < 50 | Yes | Yes | IDH1 Mutant | Codel |
| CGGA_P172 | 1107 | Alive | Primary | LGG | WHO III | Male | < 50 | Yes | Yes | IDH1 Mutant | NA |
| CGGA_P174 | 1120 | Alive | Primary | LGG | WHO II | Male | < 50 | Yes | Yes | IDH1 Mutant | Codel |
| CGGA_P178 | 1080 | Alive | Primary | GBM | WHO IV | Female | 50-59 | Yes | Yes | IDH WT | NA |
| CGGA_P179 | 1092 | Alive | Primary | LGG | WHO II | Female | 50-59 | Yes | Yes | IDH1 Mutant | Non-codel |
| CGGA_P182 | 1150 | Alive | Recurrent | rGBM | WHO IV | Female | 50-59 | Yes | Yes | IDH WT | Non-codel |
| CGGA_P183 | 1150 | Alive | Primary | LGG | WHO III | Male | 50-59 | Yes | Yes | IDH1 Mutant | Codel |
| CGGA_P21 | 1553 | Alive | Primary | LGG | WHO II | Male | < 50 | Yes | Yes | IDH1 Mutant | Non-codel |
| CGGA_P279 | 972 | Alive | Recurrent | rLGG | WHO III | Female | < 50 | Yes | Yes | IDH1 Mutant | Codel |
| CGGA_P280 | 979 | Alive | Recurrent | rGBM | WHO IV | Female | 50-59 | Yes | Yes | IDH WT | Non-codel |
| CGGA_P283 | 968 | Alive | Recurrent | rGBM | WHO IV | Male | < 50 | Yes | Yes | IDH WT | Non-codel |
| CGGA_P439 | 661 | Alive | Recurrent | rLGG | WHO II | Male | < 50 | Yes | Yes | IDH1 Mutant | Non-codel |
| CGGA_P446 | 646 | Alive | Recurrent | rLGG | WHO III | Female | < 50 | Yes | Yes | IDH1 Mutant | Codel |
| CGGA_P500 | 567 | Alive | Recurrent | rLGG | WHO II | Female | < 50 | Yes | Yes | IDH1 Mutant | Codel |
| CGGA_P633 | 327 | Alive | Recurrent | rLGG | WHO III | Female | < 50 | Yes | Yes | IDH1 Mutant | Non-codel |
| CGGA_P83 | 1330 | Alive | Primary | LGG | WHO II | Female | < 50 | Yes | Yes | IDH1 Mutant | Non-codel |
| CGGA_P99 | 679 | Alive | Recurrent | rGBM | WHO IV | Male | 50-59 | Yes | Yes | IDH1 Mutant | Non-codel |
| CGGA_1002 | 305 | Dead | Primary | LGG | WHO III | Female | < 50 | Yes | Yes | IDH WT | Non-codel |
| CGGA_1006 | 254 | Dead | Primary | LGG | WHO III | Male | < 50 | Yes | Yes | IDH WT | Non-codel |
| CGGA_1007 | 345 | Dead | Primary | GBM | WHO IV | Female | 50-59 | Yes | Yes | IDH WT | Non-codel |
| CGGA_1008 | 315 | Dead | Primary | GBM | WHO IV | Female | 50-59 | Yes | Yes | IDH WT | Non-codel |
| CGGA_1010 | 246 | Dead | Primary | LGG | WHO II | Male | < 50 | Yes | Yes | IDH1 Mutant | NA |
| CGGA_1013 | 1725 | Dead | Primary | LGG | WHO II | Female | < 50 | Yes | Yes | IDH1 Mutant | Non-codel |
| CGGA_1018 | 2527 | Dead | Recurrent | rLGG | WHO II | Male | 50-59 | Yes | Yes | IDH1 Mutant | Codel |
| CGGA_1023 | 681 | Dead | Primary | GBM | WHO IV | Female | 50-59 | Yes | Yes | IDH WT | Non-codel |
| CGGA_1024 | 3074 | Dead | Primary | GBM | WHO IV | Male | 60-69 | Yes | Yes | IDH WT | Non-codel |
| CGGA_1026 | 1570 | Dead | Primary | GBM | WHO IV | Male | 50-59 | Yes | Yes | IDH WT | Non-codel |
| CGGA_1035 | 567 | Dead | Primary | GBM | WHO IV | Female | 60-69 | Yes | Yes | IDH WT | Non-codel |
| CGGA_1036 | 806 | Dead | Primary | GBM | WHO IV | Male | < 50 | Yes | Yes | IDH WT | Non-codel |
| CGGA_1045 | 348 | Dead | Primary | GBM | WHO IV | Male | 70-79 | Yes | Yes | IDH WT | Non-codel |
| CGGA_1049 | 809 | Dead | Primary | GBM | WHO IV | Male | 50-59 | Yes | Yes | IDH WT | Non-codel |
| CGGA_1053 | 370 | Dead | Primary | GBM | WHO IV | Female | 60-69 | Yes | Yes | IDH WT | Non-codel |
| CGGA_1055 | 1611 | Dead | Primary | LGG | WHO III | Male | < 50 | Yes | Yes | IDH1 Mutant | Non-codel |
| CGGA_1057 | 1549 | Dead | Primary | LGG | WHO II | Female | 50-59 | Yes | Yes | IDH WT | Non-codel |
| CGGA_1058 | 995 | Dead | Recurrent | rLGG | WHO II | Male | < 50 | Yes | Yes | IDH1 Mutant | Non-codel |
| CGGA_1061 | 532 | Dead | Recurrent | rLGG | WHO III | Female | < 50 | Yes | Yes | IDH1 Mutant | Non-codel |
| CGGA_1072 | 1090 | Dead | Primary | GBM | WHO IV | Male | < 50 | Yes | Yes | IDH WT | Non-codel |
| CGGA_1074 | 86 | Dead | Primary | GBM | WHO IV | Male | 50-59 | Yes | Yes | IDH WT | Non-codel |
| CGGA_1075 | 398 | Dead | Primary | GBM | WHO IV | Male | 70-79 | Yes | Yes | IDH WT | Non-codel |
| CGGA_1078 | 315 | Dead | Recurrent | rGBM | WHO IV | Female | 60-69 | Yes | Yes | IDH WT | Non-codel |
| CGGA_108 | 366 | Dead | Recurrent | rLGG | WHO III | Male | < 50 | Yes | Yes | IDH WT | Non-codel |
| CGGA_1083 | 450 | Dead | Primary | GBM | WHO IV | Male | 50-59 | Yes | Yes | IDH WT | Non-codel |
| CGGA_1086 | 1977 | Dead | Primary | GBM | WHO IV | Female | 60-69 | Yes | Yes | IDH WT | NA |
| CGGA_1091 | 1116 | Dead | Primary | GBM | WHO IV | Female | 50-59 | Yes | Yes | IDH WT | Non-codel |
| CGGA_1095 | 2778 | Dead | NA | NA | NA | Male | < 50 | Yes | Yes | IDH1 Mutant | Non-codel |
| CGGA_1097 | 1378 | Dead | Primary | LGG | WHO II | Male | < 50 | Yes | Yes | IDH WT | Non-codel |
| CGGA_1103 | 585 | Dead | Primary | GBM | WHO IV | Female | < 50 | Yes | Yes | IDH1 Mutant | Non-codel |
| CGGA_1105 | 609 | Dead | Secondary | sGBM | WHO IV | Male | < 50 | Yes | Yes | IDH1 Mutant | Non-codel |
| CGGA_1106 | 420 | Dead | Primary | GBM | WHO IV | Male | < 50 | Yes | Yes | IDH WT | Non-codel |
| CGGA_1108 | 684 | Dead | Primary | LGG | WHO III | Female | < 50 | Yes | Yes | IDH WT | Non-codel |
| CGGA_1111 | 2696 | Dead | Primary | LGG | WHO III | Male | < 50 | Yes | Yes | IDH1 Mutant | Non-codel |
| CGGA_1114 | 460 | Dead | Primary | GBM | WHO IV | Male | 50-59 | Yes | Yes | IDH WT | Non-codel |
| CGGA_1116 | 248 | Dead | Recurrent | rGBM | WHO IV | Female | < 50 | Yes | Yes | IDH1 Mutant | Non-codel |
| CGGA_1119 | 422 | Dead | Recurrent | rGBM | WHO IV | Male | 50-59 | Yes | Yes | IDH1 Mutant | Codel |
| CGGA_112 | 1534 | Dead | Primary | LGG | WHO II | Male | < 50 | Yes | Yes | IDH1 Mutant | Non-codel |
| CGGA_1120 | 422 | Dead | Primary | LGG | WHO III | Female | 50-59 | Yes | Yes | IDH1 Mutant | NA |
| CGGA_1124 | 484 | Dead | Primary | GBM | WHO IV | Male | < 50 | Yes | Yes | IDH WT | Non-codel |
| CGGA_1126 | 784 | Dead | Primary | LGG | WHO III | Male | < 50 | Yes | Yes | IDH1 Mutant | Non-codel |
| CGGA_1127 | 2630 | Dead | Primary | LGG | WHO II | Male | < 50 | Yes | Yes | NA | Codel |
| CGGA_1135 | 1244 | Dead | Primary | GBM | WHO IV | Male | < 50 | Yes | Yes | IDH WT | Non-codel |
| CGGA_1138 | 411 | Dead | Primary | GBM | WHO IV | Male | 50-59 | Yes | Yes | IDH WT | Non-codel |
| CGGA_1139 | 1196 | Dead | Primary | GBM | WHO IV | Female | 50-59 | Yes | Yes | IDH WT | Non-codel |
| CGGA_1142 | 1005 | Dead | Primary | GBM | WHO IV | Male | 60-69 | Yes | Yes | IDH WT | Non-codel |
| CGGA_1145 | 239 | Dead | Recurrent | rLGG | WHO III | Male | < 50 | Yes | Yes | IDH WT | Non-codel |
| CGGA_1148 | 596 | Dead | Primary | LGG | WHO II | Male | < 50 | Yes | Yes | IDH1 Mutant | Non-codel |
| CGGA_1155 | 1373 | Dead | Primary | LGG | WHO III | Male | < 50 | Yes | Yes | IDH1 Mutant | Codel |
| CGGA_1157 | 727 | Dead | Primary | LGG | WHO II | Male | 60-69 | Yes | Yes | IDH WT | Non-codel |
| CGGA_1169 | 1765 | Dead | Primary | LGG | WHO III | Male | < 50 | Yes | Yes | IDH1 Mutant | Non-codel |
| CGGA_1170 | 346 | Dead | Secondary | sGBM | WHO IV | Male | < 50 | Yes | Yes | IDH1 Mutant | Non-codel |
| CGGA_1171 | 412 | Dead | Primary | GBM | WHO IV | Male | < 50 | Yes | Yes | IDH WT | Non-codel |
| CGGA_1177 | 449 | Dead | Recurrent | rGBM | WHO IV | Male | < 50 | Yes | Yes | IDH WT | Non-codel |
| CGGA_1178 | 422 | Dead | Recurrent | rLGG | WHO III | Male | < 50 | Yes | Yes | IDH1 Mutant | Non-codel |
| CGGA_1183 | 408 | Dead | Primary | LGG | WHO III | Female | < 50 | Yes | Yes | IDH WT | Non-codel |
| CGGA_1184 | 156 | Dead | Recurrent | rLGG | WHO III | Male | < 50 | Yes | Yes | IDH1 Mutant | Non-codel |
| CGGA_1185 | 731 | Dead | Recurrent | rLGG | WHO III | Female | 50-59 | Yes | Yes | IDH WT | Non-codel |
| CGGA_1188 | 256 | Dead | Secondary | sGBM | WHO IV | Female | < 50 | Yes | Yes | IDH1 Mutant | Non-codel |
| CGGA_1197 | 942 | Dead | Secondary | sGBM | WHO IV | Male | < 50 | Yes | Yes | IDH1 Mutant | Codel |
| CGGA_120 | 288 | Dead | Recurrent | rGBM | WHO IV | Male | < 50 | Yes | Yes | IDH WT | Non-codel |
| CGGA_1205 | 929 | Dead | Primary | LGG | WHO III | Female | 50-59 | Yes | Yes | IDH WT | NA |
| CGGA_1208 | 161 | Dead | Recurrent | rGBM | WHO IV | Male | < 50 | Yes | Yes | IDH WT | Non-codel |
| CGGA_1212 | 2292 | Dead | Primary | LGG | WHO III | Male | < 50 | Yes | Yes | IDH1 Mutant | Non-codel |
| CGGA_1214 | 604 | Dead | Primary | GBM | WHO IV | Female | < 50 | Yes | Yes | IDH WT | Non-codel |
| CGGA_1215 | 29 | Dead | Recurrent | rLGG | WHO III | Male | < 50 | Yes | Yes | IDH1 Mutant | Non-codel |
| CGGA_1216 | 312 | Dead | Primary | GBM | WHO IV | Male | < 50 | Yes | Yes | IDH WT | Non-codel |
| CGGA_1219 | 2120 | Dead | Primary | GBM | WHO IV | Female | 50-59 | Yes | Yes | IDH1 Mutant | Non-codel |
| CGGA_1223 | 682 | Dead | Primary | LGG | WHO III | Male | < 50 | Yes | Yes | IDH WT | Non-codel |
| CGGA_1224 | 271 | Dead | Primary | GBM | WHO IV | Male | 50-59 | Yes | Yes | IDH WT | Non-codel |
| CGGA_1226 | 670 | Dead | Primary | LGG | WHO II | Female | < 50 | Yes | Yes | IDH WT | Non-codel |
| CGGA_1231 | 2279 | Dead | Recurrent | rLGG | WHO III | Female | < 50 | Yes | Yes | IDH WT | Non-codel |
| CGGA_1232 | 2067 | Dead | Primary | LGG | WHO III | Male | < 50 | Yes | Yes | IDH1 Mutant | Non-codel |
| CGGA_1234 | 705 | Dead | Primary | GBM | WHO IV | Male | < 50 | Yes | Yes | IDH WT | Non-codel |
| CGGA_1237 | 296 | Dead | Primary | GBM | WHO IV | Male | 60-69 | Yes | Yes | IDH WT | Non-codel |
| CGGA_1248 | 138 | Dead | Recurrent | rGBM | WHO IV | Male | 50-59 | Yes | Yes | IDH WT | Non-codel |
| CGGA_1251 | 272 | Dead | Primary | GBM | WHO IV | Female | < 50 | Yes | Yes | IDH WT | Non-codel |
| CGGA_1255 | 118 | Dead | Recurrent | rGBM | WHO IV | Male | < 50 | Yes | Yes | IDH WT | Non-codel |
| CGGA_1257 | 192 | Dead | Recurrent | rGBM | WHO IV | Male | 50-59 | Yes | Yes | IDH WT | Non-codel |
| CGGA_1260 | 177 | Dead | Recurrent | rGBM | WHO IV | Male | < 50 | Yes | Yes | IDH WT | Non-codel |
| CGGA_1270 | 188 | Dead | Primary | GBM | WHO IV | Female | 60-69 | Yes | Yes | IDH WT | Non-codel |
| CGGA_1271 | 523 | Dead | Recurrent | rGBM | WHO IV | Male | < 50 | Yes | Yes | IDH WT | Non-codel |
| CGGA_1275 | 183 | Dead | Primary | GBM | WHO IV | Male | 70-79 | Yes | Yes | IDH WT | Non-codel |
| CGGA_1280 | 447 | Dead | Primary | LGG | WHO III | Male | 70-79 | Yes | Yes | IDH WT | Non-codel |
| CGGA_1282 | 1116 | Dead | Primary | GBM | WHO IV | Female | < 50 | Yes | Yes | IDH WT | Non-codel |
| CGGA_1283 | 20 | Dead | Secondary | sGBM | WHO IV | Male | < 50 | Yes | Yes | IDH WT | Non-codel |
| CGGA_1287 | 533 | Dead | Primary | GBM | WHO IV | Male | < 50 | Yes | Yes | IDH1 Mutant | Non-codel |
| CGGA_1295 | 310 | Dead | Recurrent | rLGG | WHO III | Male | < 50 | Yes | Yes | IDH1 Mutant | Non-codel |
| CGGA_1299 | 550 | Dead | Primary | GBM | WHO IV | Male | 50-59 | Yes | Yes | IDH WT | Non-codel |
| CGGA_1300 | 1793 | Dead | Primary | LGG | WHO III | Male | < 50 | Yes | Yes | IDH1 Mutant | Non-codel |
| CGGA_1301 | 239 | Dead | Secondary | sGBM | WHO IV | Male | < 50 | Yes | Yes | IDH WT | Non-codel |
| CGGA_1311 | 1080 | Dead | Recurrent | rLGG | WHO III | Female | < 50 | Yes | Yes | IDH1 Mutant | Codel |
| CGGA_1313 | 379 | Dead | Primary | GBM | WHO IV | Male | < 50 | Yes | Yes | IDH WT | Non-codel |
| CGGA_1321 | 702 | Dead | Primary | LGG | WHO III | Male | < 50 | Yes | Yes | IDH1 Mutant | Non-codel |
| CGGA_1322 | 253 | Dead | Primary | LGG | WHO III | Male | < 50 | Yes | Yes | IDH1 Mutant | Non-codel |
| CGGA_1324 | 848 | Dead | Secondary | sGBM | WHO IV | Male | < 50 | Yes | Yes | IDH1 Mutant | Codel |
| CGGA_1329 | 326 | Dead | Recurrent | rLGG | WHO III | Female | < 50 | Yes | Yes | IDH1 Mutant | Non-codel |
| CGGA_1334 | 146 | Dead | Primary | LGG | WHO II | Female | < 50 | Yes | Yes | IDH WT | Non-codel |
| CGGA_1337 | 2172 | Dead | Recurrent | rGBM | WHO IV | Male | 60-69 | Yes | Yes | NA | Non-codel |
| CGGA_1338 | 1122 | Dead | Primary | GBM | WHO IV | Female | 60-69 | Yes | Yes | IDH WT | Non-codel |
| CGGA_1340 | 1109 | Dead | Primary | LGG | WHO III | Male | < 50 | Yes | Yes | IDH WT | Non-codel |
| CGGA_1342 | 186 | Dead | Primary | GBM | WHO IV | Male | < 50 | Yes | Yes | IDH WT | Non-codel |
| CGGA_1343 | 94 | Dead | Recurrent | rGBM | WHO IV | Male | < 50 | Yes | Yes | IDH WT | Non-codel |
| CGGA_1346 | 284 | Dead | Secondary | sGBM | WHO IV | Female | 50-59 | Yes | Yes | IDH WT | Non-codel |
| CGGA_1353 | 1022 | Dead | Primary | GBM | WHO IV | Male | 60-69 | Yes | Yes | IDH WT | Non-codel |
| CGGA_1354 | 530 | Dead | Primary | GBM | WHO IV | Female | < 50 | Yes | Yes | IDH WT | Non-codel |
| CGGA_1359 | 343 | Dead | Recurrent | rLGG | WHO III | Male | < 50 | Yes | Yes | IDH WT | Non-codel |
| CGGA_1365 | 253 | Dead | Primary | GBM | WHO IV | Male | 50-59 | Yes | Yes | IDH WT | NA |
| CGGA_1378 | 378 | Dead | Primary | GBM | WHO IV | Male | < 50 | Yes | Yes | IDH WT | NA |
| CGGA_1380 | 291 | Dead | Primary | GBM | WHO IV | Male | < 50 | Yes | Yes | IDH WT | Non-codel |
| CGGA_1381 | 46 | Dead | Recurrent | rGBM | WHO IV | Female | < 50 | Yes | Yes | IDH1 Mutant | Non-codel |
| CGGA_1382 | 284 | Dead | Primary | GBM | WHO IV | Male | 50-59 | Yes | Yes | IDH WT | Non-codel |
| CGGA_1384 | 191 | Dead | Primary | GBM | WHO IV | Female | < 50 | Yes | Yes | IDH1 Mutant | Non-codel |
| CGGA_1387 | 90 | Dead | Recurrent | rGBM | WHO IV | Male | < 50 | Yes | Yes | IDH WT | Non-codel |
| CGGA_1388 | 485 | Dead | Recurrent | rLGG | WHO III | Male | < 50 | Yes | Yes | IDH1 Mutant | Non-codel |
| CGGA_139 | 694 | Dead | Primary | GBM | WHO IV | Male | 50-59 | Yes | Yes | IDH1 Mutant | Non-codel |
| CGGA_1392 | 473 | Dead | Primary | GBM | WHO IV | Male | 60-69 | Yes | Yes | IDH WT | Non-codel |
| CGGA_1393 | 275 | Dead | Secondary | sGBM | WHO IV | Male | < 50 | Yes | Yes | IDH WT | Non-codel |
| CGGA_1394 | 139 | Dead | Recurrent | rGBM | WHO IV | Male | < 50 | Yes | Yes | IDH1 Mutant | Non-codel |
| CGGA_1398 | 554 | Dead | Recurrent | rLGG | WHO III | Male | < 50 | Yes | Yes | IDH1 Mutant | Non-codel |
| CGGA_1401 | 1680 | Dead | Primary | LGG | WHO III | Male | < 50 | Yes | Yes | IDH1 Mutant | Non-codel |
| CGGA_1403 | 679 | Dead | Primary | GBM | WHO IV | Female | < 50 | Yes | Yes | IDH WT | Non-codel |
| CGGA_1406 | 2154 | Dead | Primary | LGG | WHO II | Male | < 50 | Yes | Yes | IDH1 Mutant | Non-codel |
| CGGA_1408 | 1362 | Dead | Primary | LGG | WHO III | Female | 60-69 | Yes | Yes | IDH WT | Non-codel |
| CGGA_1410 | 825 | Dead | Primary | GBM | WHO IV | Female | < 50 | Yes | Yes | IDH WT | Non-codel |
| CGGA_1411 | 329 | Dead | Recurrent | rLGG | WHO III | Female | < 50 | Yes | Yes | IDH WT | Non-codel |
| CGGA_1412 | 296 | Dead | Secondary | sGBM | WHO IV | Female | < 50 | Yes | Yes | IDH1 Mutant | Codel |
| CGGA_1418 | 287 | Dead | Primary | GBM | WHO IV | Female | 70-79 | Yes | Yes | IDH WT | NA |
| CGGA_1419 | 249 | Dead | Recurrent | rGBM | WHO IV | Male | 50-59 | Yes | Yes | IDH WT | Non-codel |
| CGGA_1420 | 364 | Dead | Primary | GBM | WHO IV | Male | 60-69 | Yes | Yes | IDH WT | Non-codel |
| CGGA_1422 | 204 | Dead | Primary | GBM | WHO IV | Male | 70-79 | Yes | Yes | IDH WT | NA |
| CGGA_1425 | 640 | Dead | Primary | GBM | WHO IV | Female | < 50 | Yes | Yes | IDH WT | Codel |
| CGGA_1426 | 133 | Dead | Primary | GBM | WHO IV | Female | 50-59 | Yes | Yes | IDH WT | Non-codel |
| CGGA_1430 | 162 | Dead | Recurrent | rGBM | WHO IV | Female | < 50 | Yes | Yes | IDH WT | Non-codel |
| CGGA_1431 | 2048 | Dead | Primary | LGG | WHO III | Male | < 50 | Yes | Yes | IDH1 Mutant | Non-codel |
| CGGA_1433 | 394 | Dead | Primary | GBM | WHO IV | Female | 70-79 | Yes | Yes | IDH WT | Non-codel |
| CGGA_1434 | 67 | Dead | Recurrent | rLGG | WHO III | Male | 60-69 | Yes | Yes | IDH WT | Non-codel |
| CGGA_1437 | 1037 | Dead | Recurrent | rLGG | WHO III | Male | < 50 | Yes | Yes | IDH WT | Non-codel |
| CGGA_1441 | 1882 | Dead | Primary | GBM | WHO IV | Male | 70-79 | Yes | Yes | IDH WT | Non-codel |
| CGGA_1444 | 378 | Dead | Primary | GBM | WHO IV | Female | 60-69 | Yes | Yes | IDH WT | Non-codel |
| CGGA_1447 | 308 | Dead | Recurrent | rLGG | WHO III | Male | < 50 | Yes | Yes | IDH WT | NA |
| CGGA_1450 | 957 | Dead | Secondary | sGBM | WHO IV | Male | < 50 | Yes | Yes | IDH1 Mutant | Codel |
| CGGA_1451 | 438 | Dead | Primary | GBM | WHO IV | Female | < 50 | Yes | Yes | IDH WT | Non-codel |
| CGGA_1452 | 468 | Dead | Primary | GBM | WHO IV | Male | 50-59 | Yes | Yes | IDH WT | Non-codel |
| CGGA_1457 | 312 | Dead | Primary | GBM | WHO IV | Male | 60-69 | Yes | Yes | IDH WT | Non-codel |
| CGGA_1458 | 127 | Dead | Recurrent | rLGG | WHO III | Female | 50-59 | Yes | Yes | IDH1 Mutant | Codel |
| CGGA_1459 | 503 | Dead | Recurrent | rLGG | WHO III | Male | 50-59 | Yes | Yes | IDH WT | Non-codel |
| CGGA_1460 | 209 | Dead | Secondary | sGBM | WHO IV | Male | 50-59 | Yes | Yes | IDH WT | Non-codel |
| CGGA_1461 | 226 | Dead | Primary | GBM | WHO IV | Female | 60-69 | Yes | Yes | IDH WT | Non-codel |
| CGGA_1462 | 174 | Dead | Primary | GBM | WHO IV | Male | < 50 | Yes | Yes | IDH WT | Non-codel |
| CGGA_1473 | 265 | Dead | Recurrent | rLGG | WHO III | Female | < 50 | Yes | Yes | NA | Non-codel |
| CGGA_1477 | 1194 | Dead | Primary | LGG | WHO II | Male | < 50 | Yes | Yes | NA | Non-codel |
| CGGA_1478 | 542 | Dead | Primary | GBM | WHO IV | Female | 70-79 | Yes | Yes | IDH WT | Non-codel |
| CGGA_1480 | 405 | Dead | Recurrent | rGBM | WHO IV | Male | < 50 | Yes | Yes | IDH WT | Non-codel |
| CGGA_1486 | 184 | Dead | Primary | GBM | WHO IV | Male | < 50 | Yes | Yes | IDH WT | NA |
| CGGA_1487 | 520 | Dead | Recurrent | rLGG | WHO III | Female | < 50 | Yes | Yes | IDH WT | Non-codel |
| CGGA_1491 | 246 | Dead | Primary | GBM | WHO IV | Male | < 50 | Yes | Yes | IDH1 Mutant | Non-codel |
| CGGA_1492 | 268 | Dead | Recurrent | rGBM | WHO IV | Female | < 50 | Yes | Yes | IDH WT | Non-codel |
| CGGA_1494 | 269 | Dead | Primary | GBM | WHO IV | Male | < 50 | Yes | Yes | IDH WT | NA |
| CGGA_1496 | 330 | Dead | Recurrent | rGBM | WHO IV | Female | < 50 | Yes | Yes | IDH1 Mutant | Codel |
| CGGA_1497 | 1178 | Dead | Primary | LGG | WHO III | Female | < 50 | Yes | Yes | IDH1 Mutant | Non-codel |
| CGGA_1500 | 108 | Dead | Primary | GBM | WHO IV | Female | < 50 | Yes | Yes | IDH WT | Non-codel |
| CGGA_1501 | 222 | Dead | Primary | GBM | WHO IV | Male | 50-59 | Yes | Yes | NA | Non-codel |
| CGGA_1503 | 777 | Dead | Primary | GBM | WHO IV | Male | < 50 | Yes | Yes | IDH WT | NA |
| CGGA_1505 | 172 | Dead | Recurrent | rGBM | WHO IV | Male | < 50 | Yes | Yes | IDH1 Mutant | Non-codel |
| CGGA_1507 | 715 | Dead | Recurrent | rGBM | WHO IV | Female | < 50 | Yes | Yes | IDH WT | Non-codel |
| CGGA_1510 | 271 | Dead | Recurrent | rLGG | WHO III | Male | < 50 | Yes | Yes | IDH1 Mutant | Non-codel |
| CGGA_1518 | 411 | Dead | Primary | LGG | WHO III | Female | < 50 | Yes | Yes | IDH WT | Non-codel |
| CGGA_1520 | 297 | Dead | Recurrent | rGBM | WHO IV | Female | 60-69 | Yes | Yes | IDH WT | Non-codel |
| CGGA_1521 | 205 | Dead | Primary | GBM | WHO IV | Female | 60-69 | Yes | Yes | IDH WT | Non-codel |
| CGGA_1524 | 1004 | Dead | Recurrent | rLGG | WHO III | Male | < 50 | Yes | Yes | IDH1 Mutant | Codel |
| CGGA_1525 | 1686 | Dead | Primary | LGG | WHO III | Male | < 50 | Yes | Yes | NA | Non-codel |
| CGGA_1527 | 1714 | Dead | Primary | LGG | WHO III | Male | < 50 | Yes | Yes | IDH1 Mutant | Non-codel |
| CGGA_1533 | 1217 | Dead | Recurrent | rLGG | WHO III | Female | 60-69 | Yes | Yes | IDH WT | Non-codel |
| CGGA_1535 | 232 | Dead | Recurrent | rGBM | WHO IV | Male | < 50 | Yes | Yes | IDH1 Mutant | Non-codel |
| CGGA_1538 | 247 | Dead | Recurrent | rGBM | WHO IV | Female | 50-59 | Yes | Yes | IDH WT | Non-codel |
| CGGA_1541 | 67 | Dead | Recurrent | rGBM | WHO IV | Female | 50-59 | Yes | Yes | IDH WT | Non-codel |
| CGGA_1548 | 1054 | Dead | Primary | GBM | WHO IV | Male | 50-59 | Yes | Yes | IDH WT | Non-codel |
| CGGA_1551 | 347 | Dead | Primary | GBM | WHO IV | Female | 50-59 | Yes | Yes | NA | Non-codel |
| CGGA_1553 | 540 | Dead | Recurrent | rLGG | WHO III | Male | < 50 | Yes | Yes | IDH1 Mutant | Non-codel |
| CGGA_1554 | 824 | Dead | Recurrent | rLGG | WHO III | Male | < 50 | Yes | Yes | IDH1 Mutant | Codel |
| CGGA_1558 | 130 | Dead | Recurrent | rGBM | WHO IV | Male | 70-79 | Yes | Yes | IDH WT | Non-codel |
| CGGA_1560 | 459 | Dead | Primary | GBM | WHO IV | Female | < 50 | Yes | Yes | IDH1 Mutant | Non-codel |
| CGGA_1564 | 190 | Dead | Primary | GBM | WHO IV | Male | < 50 | Yes | Yes | IDH WT | Non-codel |
| CGGA_1567 | 840 | Dead | Primary | LGG | WHO III | Female | < 50 | Yes | Yes | IDH1 Mutant | Non-codel |
| CGGA_157 | 914 | Dead | Recurrent | rLGG | WHO II | Male | < 50 | Yes | Yes | NA | Non-codel |
| CGGA_1572 | 160 | Dead | Recurrent | rGBM | WHO IV | Female | 60-69 | Yes | Yes | IDH WT | Non-codel |
| CGGA_1580 | 1581 | Dead | Primary | LGG | WHO III | Female | < 50 | Yes | Yes | IDH1 Mutant | Non-codel |
| CGGA_1583 | 1606 | Dead | Primary | LGG | WHO II | Female | 50-59 | Yes | Yes | NA | Non-codel |
| CGGA_1586 | 232 | Dead | Primary | GBM | WHO IV | Female | 50-59 | Yes | Yes | IDH WT | Non-codel |
| CGGA_1589 | 342 | Dead | Recurrent | rLGG | WHO II | Male | < 50 | Yes | Yes | IDH WT | Non-codel |
| CGGA_1591 | 1133 | Dead | Recurrent | rLGG | WHO III | Male | 50-59 | Yes | Yes | IDH1 Mutant | Non-codel |
| CGGA_1592 | 1588 | Dead | Recurrent | rLGG | WHO III | Male | < 50 | Yes | Yes | IDH1 Mutant | Codel |
| CGGA_1596 | 205 | Dead | Primary | GBM | WHO IV | Male | 60-69 | Yes | Yes | IDH WT | Non-codel |
| CGGA_1597 | 174 | Dead | Primary | GBM | WHO IV | Male | 50-59 | Yes | Yes | IDH WT | Non-codel |
| CGGA_1598 | 564 | Dead | Primary | LGG | WHO III | Male | < 50 | Yes | Yes | IDH WT | Non-codel |
| CGGA_1601 | 710 | Dead | Primary | GBM | WHO IV | Male | 60-69 | Yes | Yes | IDH WT | Non-codel |
| CGGA_1603 | 356 | Dead | Recurrent | rGBM | WHO IV | Male | < 50 | Yes | Yes | IDH WT | Non-codel |
| CGGA_1604 | 740 | Dead | Recurrent | rGBM | WHO IV | Male | < 50 | Yes | Yes | IDH1 Mutant | Non-codel |
| CGGA_1605 | 321 | Dead | Recurrent | rGBM | WHO IV | Male | < 50 | Yes | Yes | IDH WT | Non-codel |
| CGGA_1606 | 471 | Dead | Primary | LGG | WHO III | Female | < 50 | Yes | Yes | IDH WT | Non-codel |
| CGGA_1607 | 1333 | Dead | Recurrent | rLGG | WHO II | Female | < 50 | Yes | Yes | IDH1 Mutant | Codel |
| CGGA_1610 | 1058 | Dead | Recurrent | rLGG | WHO III | Male | < 50 | Yes | Yes | NA | Codel |
| CGGA_1611 | 1158 | Dead | Recurrent | rGBM | WHO IV | Female | < 50 | Yes | Yes | IDH1 Mutant | Non-codel |
| CGGA_1612 | 718 | Dead | Primary | GBM | WHO IV | Male | 60-69 | Yes | Yes | IDH WT | Non-codel |
| CGGA_1614 | 1079 | Dead | Recurrent | rLGG | WHO III | Male | 50-59 | Yes | Yes | IDH1 Mutant | Non-codel |
| CGGA_1615 | 586 | Dead | Recurrent | rGBM | WHO IV | Male | < 50 | Yes | Yes | IDH WT | Codel |
| CGGA_1619 | 766 | Dead | Primary | LGG | WHO III | Male | < 50 | Yes | Yes | IDH1 Mutant | Non-codel |
| CGGA_1624 | 87 | Dead | Recurrent | rGBM | WHO IV | Female | < 50 | Yes | Yes | IDH1 Mutant | Non-codel |
| CGGA_1626 | 696 | Dead | Primary | GBM | WHO IV | Male | 60-69 | Yes | Yes | IDH WT | Non-codel |
| CGGA_1635 | 332 | Dead | Primary | GBM | WHO IV | Female | < 50 | Yes | Yes | IDH WT | NA |
| CGGA_1641 | 291 | Dead | Recurrent | rGBM | WHO IV | Female | 50-59 | Yes | Yes | IDH WT | Non-codel |
| CGGA_1644 | 173 | Dead | Primary | GBM | WHO IV | Male | < 50 | Yes | Yes | IDH WT | Non-codel |
| CGGA_1650 | 1283 | Dead | Primary | GBM | WHO IV | Male | < 50 | Yes | Yes | IDH1 Mutant | Non-codel |
| CGGA_1651 | 842 | Dead | Recurrent | rLGG | WHO II | Female | < 50 | Yes | Yes | IDH1 Mutant | Non-codel |
| CGGA_1656 | 149 | Dead | Recurrent | rGBM | WHO IV | Male | 50-59 | Yes | Yes | IDH WT | Non-codel |
| CGGA_1658 | 1574 | Dead | Recurrent | rGBM | WHO IV | Female | 60-69 | Yes | Yes | IDH WT | Non-codel |
| CGGA_1659 | 387 | Dead | Recurrent | rGBM | WHO IV | Male | < 50 | Yes | Yes | IDH WT | Non-codel |
| CGGA_1663 | 290 | Dead | Recurrent | rGBM | WHO IV | Male | 60-69 | Yes | Yes | IDH WT | Non-codel |
| CGGA_1666 | 249 | Dead | Primary | GBM | WHO IV | Male | 60-69 | Yes | Yes | IDH WT | NA |
| CGGA_1670 | 365 | Dead | Recurrent | rLGG | WHO III | Female | < 50 | Yes | Yes | IDH WT | Non-codel |
| CGGA_1678 | 657 | Dead | Primary | GBM | WHO IV | Male | 50-59 | Yes | Yes | IDH WT | NA |
| CGGA_1680 | 596 | Dead | Primary | LGG | WHO III | Male | < 50 | Yes | Yes | IDH1 Mutant | Non-codel |
| CGGA_1681 | 346 | Dead | Primary | GBM | WHO IV | Female | 50-59 | Yes | Yes | IDH WT | Codel |
| CGGA_1682 | 345 | Dead | Recurrent | rGBM | WHO IV | Male | 60-69 | Yes | Yes | IDH WT | Non-codel |
| CGGA_1685 | 225 | Dead | Recurrent | rLGG | WHO III | Male | < 50 | Yes | Yes | IDH1 Mutant | Non-codel |
| CGGA_1688 | 51 | Dead | Recurrent | rLGG | WHO III | Female | 50-59 | Yes | Yes | IDH WT | Non-codel |
| CGGA_1690 | 592 | Dead | Primary | GBM | WHO IV | Male | 60-69 | Yes | Yes | IDH WT | Non-codel |
| CGGA_1693 | 517 | Dead | Primary | LGG | WHO III | Male | 60-69 | Yes | Yes | NA | Non-codel |
| CGGA_1694 | 624 | Dead | Primary | GBM | WHO IV | Male | 50-59 | Yes | Yes | IDH WT | Non-codel |
| CGGA_1702 | 168 | Dead | Recurrent | rGBM | WHO IV | Female | < 50 | Yes | Yes | IDH WT | Non-codel |
| CGGA_1708 | 1122 | Dead | Primary | GBM | WHO IV | Female | 50-59 | Yes | Yes | IDH WT | Non-codel |
| CGGA_1709 | 415 | Dead | Primary | GBM | WHO IV | Male | < 50 | Yes | Yes | IDH WT | Non-codel |
| CGGA_1713 | 332 | Dead | Primary | GBM | WHO IV | Male | 60-69 | Yes | Yes | IDH WT | Non-codel |
| CGGA_1716 | 951 | Dead | Recurrent | rLGG | WHO III | Male | < 50 | Yes | Yes | IDH1 Mutant | Non-codel |
| CGGA_1722 | 349 | Dead | Primary | GBM | WHO IV | Female | 60-69 | Yes | Yes | IDH WT | NA |
| CGGA_1728 | 917 | Dead | Primary | GBM | WHO IV | Male | < 50 | Yes | Yes | IDH1 Mutant | Non-codel |
| CGGA_1729 | 244 | Dead | Recurrent | rGBM | WHO IV | Male | 50-59 | Yes | Yes | IDH WT | Non-codel |
| CGGA_1735 | 813 | Dead | Primary | GBM | WHO IV | Male | 50-59 | Yes | Yes | IDH WT | Non-codel |
| CGGA_1736 | 938 | Dead | Primary | GBM | WHO IV | Female | 50-59 | Yes | Yes | IDH WT | Non-codel |
| CGGA_1738 | 469 | Dead | Primary | LGG | WHO III | Male | < 50 | Yes | Yes | IDH WT | Non-codel |
| CGGA_1739 | 515 | Dead | Recurrent | rLGG | WHO III | Male | < 50 | Yes | Yes | IDH1 Mutant | Non-codel |
| CGGA_1747 | 293 | Dead | Primary | LGG | WHO III | Female | < 50 | Yes | Yes | NA | Non-codel |
| CGGA_1749 | 401 | Dead | Primary | GBM | WHO IV | Female | < 50 | Yes | Yes | IDH WT | Non-codel |
| CGGA_1750 | 250 | Dead | Primary | GBM | WHO IV | Female | 50-59 | Yes | Yes | IDH WT | Non-codel |
| CGGA_1758 | 414 | Dead | Primary | GBM | WHO IV | Female | < 50 | Yes | Yes | IDH WT | Non-codel |
| CGGA_1760 | 459 | Dead | Recurrent | rLGG | WHO II | Male | 50-59 | Yes | Yes | IDH WT | Non-codel |
| CGGA_1764 | 710 | Dead | Primary | GBM | WHO IV | Male | < 50 | Yes | Yes | IDH1 Mutant | Non-codel |
| CGGA_1771 | 1068 | Dead | Recurrent | rLGG | WHO III | Male | < 50 | Yes | Yes | IDH1 Mutant | Non-codel |
| CGGA_1773 | 104 | Dead | Recurrent | rGBM | WHO IV | Female | < 50 | Yes | Yes | IDH WT | Non-codel |
| CGGA_1776 | 259 | Dead | Recurrent | rGBM | WHO IV | Male | < 50 | Yes | Yes | IDH WT | Non-codel |
| CGGA_1785 | 329 | Dead | Recurrent | rGBM | WHO IV | Male | < 50 | Yes | Yes | IDH1 Mutant | Non-codel |
| CGGA_1791 | 855 | Dead | Primary | LGG | WHO III | Male | 50-59 | Yes | Yes | IDH WT | NA |
| CGGA_1809 | 343 | Dead | Primary | LGG | WHO III | Male | < 50 | Yes | Yes | IDH WT | Non-codel |
| CGGA_1811 | 304 | Dead | Recurrent | rGBM | WHO IV | Male | 60-69 | Yes | Yes | IDH WT | Non-codel |
| CGGA_1814 | 221 | Dead | Recurrent | rGBM | WHO IV | Female | < 50 | Yes | Yes | IDH WT | Non-codel |
| CGGA_1815 | 185 | Dead | Recurrent | rGBM | WHO IV | Female | < 50 | Yes | Yes | IDH WT | Non-codel |
| CGGA_1817 | 399 | Dead | Primary | GBM | WHO IV | Female | 70-79 | Yes | Yes | IDH WT | Non-codel |
| CGGA_1820 | 77 | Dead | Recurrent | rGBM | WHO IV | Female | 50-59 | Yes | Yes | IDH WT | Non-codel |
| CGGA_1833 | 494 | Dead | Primary | GBM | WHO IV | Female | 60-69 | Yes | Yes | IDH WT | Non-codel |
| CGGA_1857 | 157 | Dead | Recurrent | rGBM | WHO IV | Male | < 50 | Yes | Yes | IDH WT | Non-codel |
| CGGA_1862 | 110 | Dead | Primary | LGG | WHO III | Male | < 50 | Yes | Yes | IDH WT | Non-codel |
| CGGA_1866 | 127 | Dead | Primary | GBM | WHO IV | Male | 60-69 | Yes | Yes | IDH WT | Non-codel |
| CGGA_1886 | 338 | Dead | Recurrent | rGBM | WHO IV | Male | < 50 | Yes | Yes | IDH WT | Non-codel |
| CGGA_1899 | 176 | Dead | Recurrent | rGBM | WHO IV | Male | < 50 | Yes | Yes | IDH WT | Non-codel |
| CGGA_1901 | 540 | Dead | Primary | GBM | WHO IV | Male | 60-69 | Yes | Yes | IDH WT | Non-codel |
| CGGA_1906 | 1054 | Dead | Recurrent | rLGG | WHO III | Male | 50-59 | Yes | Yes | IDH1 Mutant | Non-codel |
| CGGA_1908 | 298 | Dead | Recurrent | rGBM | WHO IV | Female | < 50 | Yes | Yes | IDH WT | Non-codel |
| CGGA_1912 | 507 | Dead | Recurrent | rGBM | WHO IV | Male | < 50 | Yes | Yes | NA | Non-codel |
| CGGA_1946 | 142 | Dead | Recurrent | rGBM | WHO IV | Female | 50-59 | Yes | Yes | IDH WT | Non-codel |
| CGGA_1953 | 73 | Dead | Recurrent | rGBM | WHO IV | Male | < 50 | Yes | Yes | IDH WT | Non-codel |
| CGGA_1955 | 457 | Dead | Recurrent | rGBM | WHO IV | Male | < 50 | Yes | Yes | NA | Non-codel |
| CGGA_1976 | 73 | Dead | Recurrent | rGBM | WHO IV | Female | 50-59 | Yes | Yes | IDH WT | Non-codel |
| CGGA_2002 | 572 | Dead | Recurrent | rLGG | WHO II | Male | < 50 | Yes | Yes | IDH1 Mutant | Non-codel |
| CGGA_2006 | 626 | Dead | Recurrent | rLGG | WHO III | Male | < 50 | Yes | Yes | IDH1 Mutant | Non-codel |
| CGGA_2046 | 251 | Dead | Recurrent | rLGG | WHO III | Male | < 50 | Yes | Yes | IDH1 Mutant | Non-codel |
| CGGA_2062 | 41 | Dead | Recurrent | rGBM | WHO IV | Female | 60-69 | Yes | Yes | IDH1 Mutant | Non-codel |
| CGGA_2075 | 266 | Dead | Recurrent | rGBM | WHO IV | Male | 60-69 | Yes | Yes | IDH WT | Non-codel |
| CGGA_2078 | 280 | Dead | Recurrent | rGBM | WHO IV | Female | 60-69 | Yes | Yes | IDH WT | Non-codel |
| CGGA_2115 | 171 | Dead | Recurrent | rGBM | WHO IV | Female | < 50 | Yes | Yes | IDH WT | Non-codel |
| CGGA_2121 | 346 | Dead | Recurrent | rLGG | WHO II | Female | < 50 | Yes | Yes | IDH1 Mutant | Non-codel |
| CGGA_235 | 2340 | Dead | Primary | LGG | WHO II | Male | < 50 | Yes | Yes | IDH1 Mutant | Codel |
| CGGA_245 | 318 | Dead | Recurrent | rLGG | WHO III | Female | < 50 | Yes | Yes | IDH1 Mutant | Non-codel |
| CGGA_247 | 609 | Dead | Primary | LGG | WHO III | Male | 60-69 | Yes | Yes | IDH1 Mutant | Non-codel |
| CGGA_279 | 1314 | Dead | Recurrent | rLGG | WHO III | Female | < 50 | Yes | Yes | IDH1 Mutant | Non-codel |
| CGGA_283 | 3470 | Dead | Primary | LGG | WHO II | Female | < 50 | Yes | Yes | IDH1 Mutant | Codel |
| CGGA_300 | 2199 | Dead | Primary | LGG | WHO II | Female | < 50 | Yes | Yes | IDH1 Mutant | Non-codel |
| CGGA_309 | 139 | Dead | NA | NA | NA | Male | < 50 | Yes | Yes | IDH WT | NA |
| CGGA_314 | 2568 | Dead | Recurrent | rLGG | WHO III | Male | < 50 | Yes | Yes | IDH1 Mutant | Codel |
| CGGA_320 | 3005 | Dead | Primary | LGG | WHO II | Male | < 50 | Yes | Yes | IDH1 Mutant | Codel |
| CGGA_330 | 1121 | Dead | Primary | LGG | WHO III | Male | < 50 | Yes | Yes | IDH WT | Non-codel |
| CGGA_336 | 2549 | Dead | Recurrent | rLGG | WHO III | Female | 50-59 | Yes | Yes | IDH1 Mutant | Codel |
| CGGA_342 | 500 | Dead | Primary | GBM | WHO IV | Female | < 50 | Yes | Yes | IDH WT | NA |
| CGGA_374 | 493 | Dead | Secondary | sGBM | WHO IV | Male | < 50 | Yes | Yes | IDH1 Mutant | Non-codel |
| CGGA_42 | 2832 | Dead | Primary | LGG | WHO II | Male | < 50 | Yes | Yes | IDH1 Mutant | Codel |
| CGGA_426 | 1560 | Dead | Primary | LGG | WHO III | Male | 50-59 | Yes | Yes | IDH WT | Non-codel |
| CGGA_448 | 354 | Dead | Primary | LGG | WHO III | Male | 60-69 | Yes | Yes | IDH WT | Non-codel |
| CGGA_458 | 419 | Dead | Recurrent | rLGG | WHO II | Male | 50-59 | Yes | Yes | IDH1 Mutant | Non-codel |
| CGGA_482 | 2511 | Dead | Primary | LGG | WHO II | Male | < 50 | Yes | Yes | IDH1 Mutant | Codel |
| CGGA_483 | 277 | Dead | Primary | GBM | WHO IV | Female | 50-59 | Yes | Yes | IDH WT | Non-codel |
| CGGA_487 | 156 | Dead | Recurrent | rGBM | WHO IV | Male | < 50 | Yes | Yes | IDH WT | Non-codel |
| CGGA_488 | 435 | Dead | Primary | LGG | WHO III | Male | < 50 | Yes | Yes | IDH WT | Non-codel |
| CGGA_491 | 1074 | Dead | Primary | GBM | WHO IV | Female | < 50 | Yes | Yes | IDH1 Mutant | Non-codel |
| CGGA_493 | 2382 | Dead | Primary | LGG | WHO II | Female | < 50 | Yes | Yes | IDH1 Mutant | Non-codel |
| CGGA_509 | 623 | Dead | Primary | GBM | WHO IV | Male | < 50 | Yes | Yes | IDH WT | Non-codel |
| CGGA_521 | 514 | Dead | Primary | LGG | WHO III | Male | < 50 | Yes | Yes | IDH WT | Non-codel |
| CGGA_545 | 555 | Dead | Recurrent | rGBM | WHO IV | Female | < 50 | Yes | Yes | IDH1 Mutant | Non-codel |
| CGGA_564 | 679 | Dead | Primary | LGG | WHO III | Male | < 50 | Yes | Yes | IDH WT | Non-codel |
| CGGA_578 | 362 | Dead | Primary | LGG | WHO III | Female | 60-69 | Yes | Yes | IDH WT | Non-codel |
| CGGA_591 | 1654 | Dead | Primary | LGG | WHO III | Female | 50-59 | Yes | Yes | IDH1 Mutant | Non-codel |
| CGGA_598 | 3677 | Dead | Primary | LGG | WHO III | Female | 60-69 | Yes | Yes | IDH1 Mutant | Codel |
| CGGA_604 | 381 | Dead | Primary | GBM | WHO IV | Male | < 50 | Yes | Yes | IDH WT | Non-codel |
| CGGA_616 | 952 | Dead | Primary | LGG | WHO III | Male | < 50 | Yes | Yes | IDH WT | Non-codel |
| CGGA_632 | 576 | Dead | Primary | LGG | WHO II | Female | < 50 | Yes | Yes | IDH1 Mutant | Non-codel |
| CGGA_633 | 1812 | Dead | Primary | LGG | WHO II | Female | 50-59 | Yes | Yes | IDH WT | Non-codel |
| CGGA_634 | 2633 | Dead | Primary | LGG | WHO III | Female | < 50 | Yes | Yes | IDH1 Mutant | Non-codel |
| CGGA_641 | 3411 | Dead | Primary | LGG | WHO III | Female | 50-59 | Yes | Yes | IDH1 Mutant | Codel |
| CGGA_643 | 331 | Dead | Primary | LGG | WHO III | Male | 50-59 | Yes | Yes | IDH WT | Non-codel |
| CGGA_652 | 476 | Dead | Recurrent | rLGG | WHO III | Male | < 50 | Yes | Yes | IDH1 Mutant | Non-codel |
| CGGA_661 | NA | Dead | Primary | LGG | WHO III | Male | < 50 | Yes | Yes | IDH WT | Non-codel |
| CGGA_669 | 607 | Dead | Recurrent | rGBM | WHO IV | Female | 50-59 | Yes | Yes | IDH WT | Codel |
| CGGA_678 | 386 | Dead | Recurrent | rLGG | WHO III | Male | 50-59 | Yes | Yes | IDH WT | Non-codel |
| CGGA_680 | 2373 | Dead | Primary | GBM | WHO IV | Male | < 50 | Yes | Yes | IDH WT | Non-codel |
| CGGA_685 | 3239 | Dead | Primary | LGG | WHO II | Female | < 50 | Yes | Yes | IDH1 Mutant | Non-codel |
| CGGA_694 | 1194 | Dead | Recurrent | rLGG | WHO II | Male | < 50 | Yes | Yes | IDH1 Mutant | Non-codel |
| CGGA_698 | 1851 | Dead | Primary | LGG | WHO II | Female | 60-69 | Yes | Yes | IDH1 Mutant | Non-codel |
| CGGA_703 | 1355 | Dead | Recurrent | rLGG | WHO III | Female | < 50 | Yes | Yes | IDH1 Mutant | Non-codel |
| CGGA_704 | 2572 | Dead | Primary | LGG | WHO II | Male | < 50 | Yes | Yes | IDH1 Mutant | Non-codel |
| CGGA_707 | 3299 | Dead | Primary | LGG | WHO II | Female | < 50 | Yes | Yes | IDH1 Mutant | Codel |
| CGGA_721 | 2552 | Dead | Primary | LGG | WHO II | Male | < 50 | Yes | Yes | IDH1 Mutant | Non-codel |
| CGGA_727 | 1023 | Dead | Primary | LGG | WHO III | Male | < 50 | Yes | Yes | IDH WT | Non-codel |
| CGGA_757 | 1047 | Dead | Primary | LGG | WHO II | Male | < 50 | Yes | Yes | IDH1 Mutant | Non-codel |
| CGGA_759 | 1263 | Dead | Primary | GBM | WHO IV | Male | < 50 | Yes | Yes | IDH1 Mutant | Non-codel |
| CGGA_799 | 1868 | Dead | Recurrent | rLGG | WHO III | Male | 50-59 | Yes | Yes | IDH1 Mutant | Codel |
| CGGA_802 | 681 | Dead | Primary | GBM | WHO IV | Male | < 50 | Yes | Yes | IDH WT | Non-codel |
| CGGA_809 | 1252 | Dead | Primary | LGG | WHO II | Male | < 50 | Yes | Yes | IDH1 Mutant | Non-codel |
| CGGA_810 | 551 | Dead | Recurrent | rLGG | WHO III | Male | < 50 | Yes | Yes | IDH WT | Non-codel |
| CGGA_822 | 2237 | Dead | Secondary | sGBM | WHO IV | Male | < 50 | Yes | Yes | IDH1 Mutant | Non-codel |
| CGGA_825 | 836 | Dead | Recurrent | rLGG | WHO III | Male | < 50 | Yes | Yes | IDH1 Mutant | Non-codel |
| CGGA_831 | 546 | Dead | Primary | GBM | WHO IV | Female | 50-59 | Yes | Yes | IDH WT | Non-codel |
| CGGA_837 | 432 | Dead | Primary | GBM | WHO IV | Male | 50-59 | Yes | Yes | IDH WT | Non-codel |
| CGGA_842 | 204 | Dead | Primary | GBM | WHO IV | Female | 60-69 | Yes | Yes | IDH WT | Non-codel |
| CGGA_881 | 252 | Dead | Recurrent | rLGG | WHO III | Female | < 50 | Yes | Yes | IDH WT | Non-codel |
| CGGA_884 | 1131 | Dead | Primary | LGG | WHO II | Female | < 50 | Yes | Yes | IDH1 Mutant | Non-codel |
| CGGA_888 | 669 | Dead | Recurrent | rLGG | WHO III | Male | < 50 | Yes | Yes | IDH1 Mutant | Codel |
| CGGA_890 | 2982 | Dead | Primary | LGG | WHO III | Male | < 50 | Yes | Yes | IDH1 Mutant | Non-codel |
| CGGA_D02 | NA | Dead | Recurrent | rGBM | WHO IV | Male | < 50 | Yes | Yes | IDH WT | Non-codel |
| CGGA_D11 | 827 | Dead | Primary | LGG | WHO III | Female | 50-59 | Yes | Yes | IDH WT | Non-codel |
| CGGA_D19 | 297 | Dead | Recurrent | rLGG | WHO II | Female | 60-69 | Yes | Yes | IDH1 Mutant | Codel |
| CGGA_D37 | 965 | Dead | Primary | GBM | WHO IV | Male | 50-59 | Yes | Yes | IDH WT | Non-codel |
| CGGA_D47 | 1048 | Dead | Primary | LGG | WHO II | Male | < 50 | Yes | Yes | IDH1 Mutant | Codel |
| CGGA_D57 | 766 | Dead | Primary | GBM | WHO IV | Male | < 50 | Yes | Yes | IDH WT | Non-codel |
| CGGA_J023 | 1028 | Dead | Primary | LGG | WHO III | Male | < 50 | Yes | Yes | IDH1 Mutant | Non-codel |
| CGGA_J50 | 1693 | Dead | Recurrent | rLGG | WHO II | Male | < 50 | Yes | Yes | IDH1 Mutant | NA |
| CGGA_J73 | 1134 | Dead | Primary | LGG | WHO III | Female | < 50 | Yes | Yes | IDH1 Mutant | NA |
| CGGA_P102 | 1269 | Dead | Primary | GBM | WHO IV | Male | < 50 | Yes | Yes | IDH1 Mutant | Non-codel |
| CGGA_P104 | 167 | Dead | Recurrent | rLGG | WHO III | Female | < 50 | Yes | Yes | IDH1 Mutant | NA |
| CGGA_P106 | 71 | Dead | Recurrent | rGBM | WHO IV | Male | < 50 | Yes | Yes | IDH WT | Non-codel |
| CGGA_P109 | 407 | Dead | Recurrent | rGBM | WHO IV | Male | < 50 | Yes | Yes | IDH1 Mutant | Non-codel |
| CGGA_P110 | 800 | Dead | Primary | LGG | WHO III | Female | 50-59 | Yes | Yes | IDH WT | Non-codel |
| CGGA_P112 | 834 | Dead | Primary | GBM | WHO IV | Male | 60-69 | Yes | Yes | IDH WT | Non-codel |
| CGGA_P136 | 842 | Dead | Primary | GBM | WHO IV | Female | < 50 | Yes | Yes | IDH WT | NA |
| CGGA_P143 | 261 | Dead | Primary | GBM | WHO IV | Female | 60-69 | Yes | Yes | IDH WT | Non-codel |
| CGGA_P15 | 723 | Dead | Primary | GBM | WHO IV | Male | < 50 | Yes | Yes | NA | Non-codel |
| CGGA_P157 | 470 | Dead | Primary | LGG | WHO III | Female | 50-59 | Yes | Yes | IDH WT | Non-codel |
| CGGA_P160 | 219 | Dead | Primary | GBM | WHO IV | Female | 70-79 | Yes | Yes | IDH WT | Non-codel |
| CGGA_P163 | 855 | Dead | Recurrent | rLGG | WHO III | Female | < 50 | Yes | Yes | IDH1 Mutant | Non-codel |
| CGGA_P165 | 598 | Dead | Primary | LGG | WHO III | Female | 50-59 | Yes | Yes | IDH1 Mutant | NA |
| CGGA_P175 | 183 | Dead | Recurrent | rGBM | WHO IV | Male | < 50 | Yes | Yes | IDH WT | NA |
| CGGA_P180 | 260 | Dead | Primary | GBM | WHO IV | Male | < 50 | Yes | Yes | IDH1 Mutant | NA |
| CGGA_P185 | 502 | Dead | Recurrent | rLGG | WHO III | Female | < 50 | Yes | Yes | IDH1 Mutant | Non-codel |
| CGGA_P205 | 583 | Dead | Primary | GBM | WHO IV | Male | 60-69 | Yes | Yes | IDH WT | NA |
| CGGA_P22 | 406 | Dead | Primary | GBM | WHO IV | Male | 60-69 | Yes | Yes | IDH WT | Non-codel |
| CGGA_P23 | 392 | Dead | Primary | LGG | WHO III | Female | 60-69 | Yes | Yes | IDH WT | NA |
| CGGA_P25 | 147 | Dead | Primary | GBM | WHO IV | Male | 60-69 | Yes | Yes | IDH WT | NA |
| CGGA_P265 | 779 | Dead | Primary | LGG | WHO III | Male | < 50 | Yes | Yes | IDH WT | NA |
| CGGA_P266 | 253 | Dead | Primary | LGG | WHO III | Female | < 50 | Yes | Yes | IDH WT | NA |
| CGGA_P269 | 511 | Dead | Recurrent | rLGG | WHO III | Female | < 50 | Yes | Yes | IDH WT | Codel |
| CGGA_P270 | 322 | Dead | Recurrent | rLGG | WHO II | Male | < 50 | Yes | Yes | IDH1 Mutant | Non-codel |
| CGGA_P295 | 273 | Dead | Recurrent | rGBM | WHO IV | Male | < 50 | Yes | Yes | IDH WT | Non-codel |
| CGGA_P30 | 404 | Dead | Primary | LGG | WHO II | Male | < 50 | Yes | Yes | IDH WT | Non-codel |
| CGGA_P308 | 563 | Dead | Recurrent | rLGG | WHO III | Male | < 50 | Yes | Yes | IDH1 Mutant | Non-codel |
| CGGA_P411 | 440 | Dead | Recurrent | rGBM | WHO IV | Female | < 50 | Yes | Yes | IDH1 Mutant | Non-codel |
| CGGA_P5 | 378 | Dead | Recurrent | rLGG | WHO III | Female | < 50 | Yes | Yes | IDH1 Mutant | Non-codel |
| CGGA_P87 | 240 | Dead | Recurrent | rGBM | WHO IV | Male | < 50 | Yes | Yes | IDH1 Mutant | Non-codel |
| CGGA_P89 | 294 | Dead | Recurrent | rGBM | WHO IV | Male | < 50 | Yes | Yes | IDH WT | Non-codel |
| CGGA_P98 | 317 | Dead | Recurrent | rLGG | WHO III | Male | < 50 | Yes | Yes | IDH1 Mutant | Non-codel |
| CGGA_1939 | NA | NA | Recurrent | rLGG | WHO II | Male | < 50 | Yes | Yes | NA | Non-codel |
| CGGA_2082 | NA | NA | Recurrent | rGBM | WHO IV | Male | < 50 | Yes | Yes | IDH WT | Non-codel |
| CGGA_2088 | NA | NA | Recurrent | rGBM | WHO IV | Female | < 50 | Yes | Yes | IDH WT | Non-codel |
